# Effectiveness of the hope groups intervention on mental health, parenting practices, and violence against children among Ukrainian parents and caregivers affected by war and displacement: a cluster randomised controlled trial

**DOI:** 10.1016/j.lanepe.2026.101783

**Published:** 2026-08-25

**Authors:** Sydney Tucker, Nicole Baldonado, Olha H. Ruina, G.J. Melendez-Torres, Seth Flaxman, Lyudmila Bryn, Laura Rawlings, Philip Goldman, Kateryna Louchnikov, Joshua Baldonado, Tetiana Machabeli, the Hope Groups Implementation Team, Alina Ivanina, Oksana Boichenko, Dariia Halych, Yuliia Kravchuk, Olena Romanenko, Yana Derzhak, Natalia Lyagul, Natalia Stupak, Svitlana Yehorova, Iryna Tokarchuk, Maryna Masiuk, Anna Yavorska, Antonina Havrish, Liubov Tishchenko, Marianna Bondarenko, Nataliia Lytvynenko, Oleg Lyagul, Olena Kharchenko, Tatiana Yastrebova, Oksana Kleymyonova, Olena Krutii, Hanna Dvoryanova, Iryna Makarieieva, Liudmyla Antoniuk, Zinaida Antikalo, Yulia Shevchenko-Szela, Oleksandr Derkach, Svitlana Derkach, Victoria Tur, Mykola Mohilevskiy, Inge Vallance, Svitlana Kharchenko, Lorraine Sherr, Oliver Ratmann, Jamie Lachman, Lucie Cluver, Susan Hillis

**Affiliations:** a Department of Social Policy and Intervention, University of Oxford, Oxford, UK; b World Without Orphans, Mount Joy, PA, USA; c NGO Neemia/NGO Nehemiah (Громадська організація “Неємія”), Uzhhorod, Ukraine; d Department of Public Health and Sport Sciences, University of Exeter, Exeter, UK; e Department of Computer Science, University of Oxford, Oxford, UK; f Children Mission (Дитяча Місія), Kyiv, Ukraine; g School of Foreign Service, Georgetown University, Washington, DC, USA; h Maestral International, Minneapolis, MN, USA; i Department of Epidemiology and Biostatistics, University of California, Berkeley, CA, USA; j The Alliance for Ukraine Without Orphans (Альянс “Україна без сиріт”), Kyiv, Ukraine; k Institute for Global Health, University College London, London, UK; l Department of Mathematics, Imperial College London, London, UK; m Centre for Social Science Research, University of Cape Town, Cape Town, South Africa; n Parenting for Lifelong Health, Oxford, United Kingdom; o Department of Psychiatry and Mental Health, University of Cape Town, Cape Town, South Africa; p Global Reference Group for Children Affected by Crisis, University of Oxford, Oxford, UK

**Keywords:** War, Displacement, Parenting, Mental health, Violence against children

## Abstract

**Background:**

Almost two-thirds of the world’s children live in conflict-affected countries, including Ukraine, where two-thirds of children were displaced after Russia’s 2022 invasion. To address the urgent need for effective approaches to support parents and protect children in war, we designed Hope Groups—a group-based mental health, parenting, and violence prevention intervention—and used a cluster randomised controlled trial (cRCT) to evaluate its effectiveness.

**Methods:**

We conducted a two-arm, parallel cRCT comprising K = 90 clusters and N = 510 parents/caregivers, who were internally displaced, externally displaced, or at home in war-affected Ukraine. Primary outcomes, assessed at baseline and after completing the 12-session intervention (endline), included caregiver mental health, parenting practices, and violence against children. Clusters were randomly assigned to immediate Hope Groups intervention (K = 45; N = 255) or waitlist control (K = 45; N = 255). Interim analyses were conducted after the first 20 clusters reached trial endline. The trial was prospectively registered with Open Science Framework (https://osf.io/uvj67) and subsequently registered on ClinicalTrials.gov to align with ICMJE guidance (NCT07470333).

**Findings:**

After completion of the 12-session intervention, the Hope Groups intervention demonstrated medium-to-large effects across all measured primary outcomes compared with the control arm. Caregivers in the intervention arm reported 57.4% lower levels of parental depression and anxiety (β = −2.85; 95% CI = −3.49, −2.22; d = −0.86); 52.7% lower levels of violence against children (β = −0.5; 95% CI = −0.64, −0.36; d = −0.43); 33% higher parenting practices (β = 1.32, 95% CI = 0.92, 1.71, d = 0.57), compared to the control arm. Response rate was high: 98.6%. There were no adverse events or harms.

**Interpretation:**

The results demonstrate that the Hope Groups intervention improves caregiver mental health, strengthens parenting practices, and reduces violence against children amidst war and displacement.

**Funding:**

This study is supported by the Global Parenting Initiative (funded by the 10.13039/100018325LEGO Foundation and Oak Foundation), the Moderna Foundation, and private donations to World Without Orphans.

**Research in context**

**Evidence before this study**

Two-thirds of the world’s children live in conflict-affected countries. In Ukraine, two-thirds of children were displaced after Russia’s 2022 invasion. Wartime conditions have acute negative impacts on parents and children, increasing the incidence of adverse childhood experiences such as violence and family dysfunction, with lifelong and intergenerational consequences. Substantial research has demonstrated the effectiveness of parental mental health and parenting skills-building interventions to improve family outcomes, but evidence is lacking in active war contexts. We searched PubMed, JSTOR, PsycINFO, and Google Scholar for randomised controlled trials published between December 1, 2010, and December 1, 2025 testing parenting interventions in humanitarian or active war contexts. Search terms included “war”, “conflict”, or “humanitarian” and “parenting” or “parent” or “caregiver”. We reviewed a meta-analysis of randomised controlled trials testing the effectiveness of parenting programmes across k = 23 RCTs in humanitarian settings, finding that parenting programmes reduced parent-perpetrated physical and psychological violence against children (standardised effect size [SES]: −0.36, 95% CI: −0.69, −0.04), while also improving positive parenting (SES: 0.48; 95% CI: 0.29, 0.67) and reducing negative parenting practices such as poor supervision (SES: −0.42; 95% CI: −0.67, −0.16).^1^ However, only five of the 23 studies occurred in active war settings, where wartime threats and emergencies present substantial challenges in both implementing and evaluating interventions, with the majority of parenting programmes tested in post-conflict settings. None of the five studies identified an intervention improving key domains of parental mental health, parenting practices, violence against children, and child well-being.

**Added value of this study**

This study is the first known randomised controlled trial evaluating a group-based mental health, parenting, and violence prevention intervention which can be flexibly adapted for participants’ needs in active war settings, incorporating in-person, digital, and hybrid delivery formats. It is the only known randomised controlled trial testing an intervention which yielded medium to large treatment effects across key domains of parental mental health, parenting practices, violence against children, and child well-being in an active war setting. In addition, this study provides a potential methodological blueprint for conducting rigorous, ethically responsible research amidst war and crisis, which prioritises participant needs and expedited delivery of evidence-based interventions.

**Implications of all the available evidence**

This study contributes to the growing body of literature demonstrating that parenting programmes are an effective approach to improve outcomes for children amidst war and displacement. It also adds to evidence supporting the integration of group-based mental health, parenting, and violence prevention content into one intervention to achieve holistic improvements for families. Additionally, this study reinforces the growing understanding that co-creating interventions with affected communities is essential for improving completion rates and participant outcomes. Co-created, group-based interventions targeting parental mental health, parenting practices, and child protection are emerging as a crucial tool to address the growing needs of families in crisis. These interventions can help advance the Sustainable Development Goals and calls for societal resilience by 2050 in an era of polycrisis.^2^

## Introduction

In 2024, the largest number of countries since World War II were in conflict.^3^ More than two-thirds of the world’s children lived in a conflict-affected country as of 2021, and one in six children lived within 50 km of a war zone in 2023.^4^^,^^5^ Russia’s 2022 invasion forced over 13 million Ukrainians to flee their homes during the first year of war.^6^ Nearly two-thirds of Ukrainian children were displaced,^7^ and 75% of Ukrainian parents reported their children showing symptoms of psychological traumatisation.^8^

Parents suffer acutely during war from stressors including psychological distress, increases in poverty, diminished ability to meet their children’s needs, and loss of critical support systems.^9^^,^^10^ In a cross-sectional survey among Ukrainian parents one-year after Russia’s 2022 invasion, 46.7% met criteria for depressive disorder, and 24.2% screened positive for anxiety disorder.^9^ War stressors have direct and substantial consequences on caregiver mental health, parenting practices, and parent-child relationships, affecting children’s health, well-being, and development.^10^, ^11^, ^12^ Furthermore, children’s risk of trauma—including sexual abuse, violent discipline, exploitation and trafficking—increase in war.^11^^,^^12^ These adverse childhood experiences have detrimental effects across the life course, including impacts on education, employment, violence perpetration and victimisation, incarceration, disease acquisition, and early mortality.^13^

Substantial evidence demonstrates the effectiveness of parental mental health and parenting skills-building interventions to reduce violence against children and improve children’s health, well-being, and development in humanitarian settings.^1^^,^^10^^,^^14^ A meta-analysis of 23 RCTs found that parenting programmes reduced parent-perpetrated physical and psychological violence against children, improved positive parenting, and reduced negative practices such as poor supervision.^1^ Parenting programmes were also associated with small reductions on child internalising behaviours, but no statistically significant effects on child externalising behaviours, suggesting a need for further adaptations to address child behavioural outcomes in complex contexts.^1^ However, within humanitarian contexts, research is often conducted post-conflict, rather than during active war; only five of the 23 studies in the meta-analysis occurred in active conflict settings—in Syria, Palestine, the Democratic Republic of Congo, Ethiopia, and Burkina Faso^1^—highlighting the need for further evidence on effective approaches to strengthen families during active war.

Despite the importance of implementing and evaluating interventions in acute settings, active wartime threats, emergencies, and uncertainties present unique challenges for intervention implementation and rigorous evaluation.^15^ Repeated displacements and ongoing violent attacks impede service delivery, often resulting in high rates of intervention incompletion and loss-to-follow-up. Specifically among Ukrainians, a recent evaluation of a remotely delivered mental health programme during war observed low completion rates of only 45% and only improved psychological distress, not anxiety, depression, or well-being.^16^ Additionally, active war creates unique ethical challenges to rigorous evaluations, such as the urgency to deliver potentially beneficial interventions to war-affected communities without delay. Identifying and scaling holistic interventions that effectively strengthen families in crisis, sustain high levels of engagement and completion, and are paired with innovative research approaches to shorten time to evidence generation should be considered an urgent public health priority.

In response to needs expressed by Ukrainian parents affected by war, we co-created a 12-session psychosocial, mental health, parenting, and violence prevention support group programme (“Hope Groups”) with Ukrainian parents and subject-matter experts from within and outside Ukraine.^17^ Following promising pre-post results showing statistically significant improvements across parental mental health, parenting practices, violence against children, and child behavioural issues,^17^ we conducted a pragmatic cluster randomised controlled trial (cRCT) to evaluate the effectiveness of Hope Groups compared to a waitlist control group.^18^ A cRCT was selected to minimise spillover and support implementation by assigning participants with existing social ties to the same cluster.

The primary objective was to estimate the effect of Hope Groups on three primary outcomes after completion of the intervention: caregiver mental health, parenting practices, and violence against children. Consistent with a pragmatic trial design, effectiveness was not defined as requiring effects across all primary outcomes; each outcome was evaluated individually to assess effectiveness. The secondary objective was to evaluate the effect of Hope Groups on caregiver well-being, child behavioural issues, and prevention of violence against women. We hypothesised that the intervention would improve all primary and secondary outcomes.

## Methods

### Reporting framework, registration, and protocol

This study is reported using the cRCT CONSORT guidelines. The trial was prospectively registered on Open Science Framework (OSF) (https://osf.io/uvj67)^19^ and subsequently registered on ClinicalTrials.gov to align with ICMJE guidance (NCT07470333). The protocol was published,^18^ and the statistical analysis plan was prospectively registered on OSF (https://osf.io/2tb3g/overview).^19^ Minor revisions to the statistical analysis plan are documented in the Statistical Appendix.

### Ethics approval

Ethical approval was obtained from the University of Oxford (Reference: R90037/RE001) and the Ukrainian Institute on Public Health Policy (Reference #00007612).

### The hope groups intervention

The Hope Groups programme aimed to: 1) strengthen parent/caregiver mental health; 2) build parenting skills to improve parent-child relationships through activities for children of all ages; and 3) reduce violence against children, particularly violent discipline, which often increases in war.^9^^,^^10^ Our theory of change posits that strengthening caregiver mental health, improving parenting practices, and reducing violence against children also improves child well-being (Supplementary Material [SM] Supplementary Fig. S1). The development of the 10-session Hope Groups intervention, adapted from Parenting for Lifelong Health, with Ukrainians affected by war is described in our pre-post study.^17^ Based on pre-post study feedback, we added two sessions focussed on strengthening marriages/partnerships during war to improve caregiver well-being and prevent violence against women. This RCT evaluates the effectiveness of the 12-session intervention. All sessions are described in Supplementary Table S1.

We recruited 30 facilitators (90% female, 50% with prior mental health training) from non-governmental organisations (NGOs), refugee shelters, faith-based organisations (FBOs), and churches through the World Without Orphans Europe network. Facilitator inclusion criteria were: 1) Ukrainian, or war-affected and fluent in Ukrainian; 2) aged ≥ 18 years; 3) able to recruit participants through affiliated networks; and 4) experienced in leading small groups with the emotional capacity to support others. Formal mental health qualifications were not required, reflecting the programme’s design to mobilise a community-based workforce capable of delivering psychosocial support at-scale in a context of widespread war-related trauma.

Programme facilitators received 14 h of Hope Groups training delivered virtually in 2–3 h sessions, covering programme content, facilitation, safeguarding, referrals, and mandated reporting. To prevent burnout, the Programme Director supported four coordinators, and coordinators supported 30 facilitators, with biweekly debriefing of challenges and encouragements to practice self-care.

Facilitators recruited participants through their own community networks, consistent with prior pragmatic, community-based trials in real-world settings, designed to strengthen external validity.^20^^,^^21^ Participant inclusion criteria were: 1) Ukrainian, or war-affected and fluent in Ukrainian; 2) aged ≥18 years; 3) a parent or caregiver for one or more children aged < 18; and 4) able to provide informed consent.

Given high mobility during war, the intervention was designed with flexible delivery to maximise retention and minimise loss-to-follow-up. Based on partner guidance, Hope Groups could be delivered in person, remotely, or in hybrid format. After recruitment, facilitators consulted participants to determine delivery format, based on their needs, preferences, and safety. In-person groups were held in shelters, community centres, churches, and homes; remote sessions via Telegram video calls; and hybrid sessions consisted of the facilitator and at least one group member meeting in-person with others joining remotely, or the group meeting in-person for ≥3 sessions and remotely for others. Facilitators were advised to hold sessions two times weekly over 6 weeks, with flexible alternatives of once weekly for 12 weeks or three times weekly for four weeks when necessary for participant schedules. Facilitators were encouraged to remain sensitive to participant needs and adjust format and frequency as required, as war impacts could disrupt participation at any time. Therefore, this study evaluates the real-world effectiveness of delivering a group-based mental health and parenting intervention designed to maximise programme completion amidst active war.

### Study design

We conducted a two-armed parallel-group cRCT comprising K = 90 clusters consisting of N = 510 participants. To enable cluster randomisation of groups stratified by facilitator with a 1:1 allocation ratio, all 30 facilitators recruited either two, four, or six clusters (k) of 5–7 participants. Clusters (k) were defined as the group of 5–7 participants who would join a “Hope Group” together. Within each facilitator strata, clusters (k) were randomly allocated with a 1:1 ratio to the intervention arm, which involved participating in Hope Groups immediately, or a waitlist control arm. To minimise spillover, during recruitment, facilitators placed participants with existing social ties (e.g., family members or neighbours) into the same Hope Group cluster. Facilitators additionally recruited each cluster from a different source (e.g., different refugee shelters).

### Randomisation and masking

Cluster randomisation stratified by facilitator was conducted using the RCT package in R (v 1.1.2) by an analyst blinded to participant identity (ST). To reduce delays, randomisation occurred by facilitator on a rolling basis. To ensure allocation concealment, each facilitator was required to recruit all their clusters (k) before randomisation. After randomisation, the analyst provided each facilitator with a list of participant IDs grouped by cluster, indicating treatment assignment. Participant blinding was not feasible. Data analysts were blinded during analysis.

### Sample size

Sample size calculations were conducted for this cRCT using the clusterPower package in R (v 0.7.0), based on estimated pre-post study effect sizes and variance parameters.^17^ Assuming a two-sided alpha of 0.05 and 80% power, the trial was powered to detect a statistically significant absolute reduction in violence against children. Estimating an average cluster size between five and six, an intraclass correlation coefficient of 0.20, a cluster coefficient of variation of 0.30, and 20% loss-to-follow-up, our target sample size was K = 90 clusters (N = 510). Powering the trial for violence against children also provided power for caregiver mental health and parenting practices, which were expected to have larger effect sizes.^17^ No target effect sizes for clinical significance were prespecified in this pragmatic trial.

### Informed consent and data collection

Participants were invited to join a mental health and parenting intervention, starting immediately or after 2–4 months, depending on random allocation. Interested individuals received an information sheet and time to ask questions. Next, facilitators texted participants a link to a self-administered, pseudonymised online Open Data Kit (ODK) survey, which opened with an informed consent form and then progressed to a 25-min baseline survey. Self-administered surveys were used to enhance anonymity and minimise social desirability bias. The survey stated that all data were pseudonymous and inaccessible to facilitators. Self-administered surveys were feasible and reliable in the Ukrainian context, as literacy and digital literacy are both high; an estimated 100% of Ukrainian adults can read and write, and an estimated 80–87% use the internet daily.^22^, ^23^, ^24^

Programme delivery to the intervention arm began within one week of randomisation. One week after the intervention arm completed the intervention, facilitators texted participants from both study arms a link to the endline survey. To support retention, participants received 180 Ukrainian hryvnia (US$5.00) per endline survey, an amount local stakeholders advised was appropriate but not coercive.

### Trial monitoring

Programme fidelity was monitored using post-session forms completed by facilitators, documenting session completion and content delivered. Adverse events were monitored by four coordinators through biweekly meetings with facilitators to assess participant well-being and identify potential adverse events, including traumatic responses to programme content or disclosures requiring mandated reporting. Facilitators and coordinators received training in safeguarding and reporting protocols, including adverse event reporting. The study team met biweekly with coordinators and monthly with a Ukrainian advisory monitoring team. No harms or adverse events were identified, thus requiring no protocol amendments.

### Outcomes

Following guidance for research in war settings, we made every effort to create a sensitive, non-triggering, and brief survey.^25^ This was especially important as post-traumatic stress disorder lowers attention and increases risk of triggers from sensitive survey questions.^25^ Ukrainian stakeholders emphasised the importance of minimising the number of questions to maximise response rates amidst active war to minimise selection bias. Primary outcomes, with corresponding intraclass correlation coefficients, are detailed in Supplementary Table S2. ***Caregiver mental health*** was measured through the Patient Health Questionnaire–4 (PHQ-4), which includes two questions on anxiety symptoms and two questions on depression symptoms. For our analysis, we aggregated responses from the four questions into a single outcome. The PHQ-4 has demonstrated reliability in the adult general population and has been used to assess mental health among war-affected Ukrainians.^26^ ***Parenting practices*** were measured through six items from the Alabama Parenting Questionnaire (APQ), including two items each from the positive parenting, parental involvement, and supervision/monitoring subscales. The six items were combined to generate a composite score of beneficial parenting practices; internal consistency was high (Cronbach’s α = 0.853). The APQ has demonstrated reliability among Ukrainian samples.^27^ ***Violence against children*** was assessed using selected behavioural indicators from the International Society for the Prevention of Child Abuse and Neglect Screening Tool–Trial version (ICAST-T), caregiver version. In collaboration with Ukrainian stakeholders, indicators from the ICAST-T physical and emotional abuse subscales were adapted into two brief, non-triggering questions appropriate for research amidst active conflict, consistent with humanitarian research recommendations.^28^ Physical violence was assessed as, “How many days in the past week did you or anyone in your household discipline your child(ren) physically, such as shaking them or hitting with your hand, stick, belt, or another object?” Emotional violence was assessed as, “How many days in the past week did you shout, yell, or scream at your child(ren)?” This approach was previously used in the Evaluation of Parenting in Crisis study^29^ and our pre-post study in Ukraine.^17^ Consistent with these studies, violence against children and parenting practices were measured as the number of days each behaviour occurred in the past week.

Secondary outcomes included caregiver well-being, measured via hopefulness about the future and coping with grief, and self-care practices; child well-being, measured via three items indicating behavioural issues from the Child and Adolescent Behaviour Inventory (internalising and externalising behaviours); prevention of violence against women, measured based on respondents’ confidence saying ‘no’ to unwanted sexual behaviour and utilisation of healthy conflict resolution strategies in romantic relationships; and healthy relationship practices, measured by couples making moments to positively connect. In the absence of feasible validated measures for the prevention of violence against women and healthy relationship practices, these constructs were developed in collaboration with local leaders, following humanitarian guidance to measure constructs that community members themselves identify as important.^28^ Additionally, we analysed the parenting subscales separately: positive parenting, parental involvement, and parental monitoring. Lastly, as an exploratory outcome, we measured use of nonviolent discipline (Parenting Young Children Scale, 1-item). Secondary outcomes are detailed in Supplementary Table S3.

Outcomes were collected at baseline and endline (one-week after completing the 12-session intervention), except violence against women outcomes, which were assessed only at endline. We also collected demographic data including age, sex, education, income, displacement status, shelter status, and location. In the absence of available measures in Ukrainian, instruments underwent forward and back translations, with review by a panel of multilingual Ukrainians to ensure accuracy and cultural clarity.

### Statistical analysis

Analyses were intention-to-treat. The primary estimand was the difference in mean outcome at endline between intervention and control arms, adjusted for baseline outcome. We estimated this using a two-level linear mixed-effects model with individuals (i) nested within clusters (j):

$$Y_{ij}=\alpha +\beta_{1}T_{j}+\beta_{2}X_{j}+{\beta_{3}W}_{ij}+\beta_{4}Z_{ij}+u_{j}+\varepsilon_{ij}$$

$$u_{j}∼N\left(0,\sigma_{u}^{2}\right)$$

$$\varepsilon_{ij}∼N\left(0,\sigma_{\varepsilon }^{2}\right)$$

Where $T_{j}$ denotes the binary intervention indicator, $X_{j}$ represents fixed effects for facilitator strata to account for stratified randomisation, $W_{ij}$ is the baseline value of the outcome, $Z_{ij}$ indicates displacement status, $u_{j}$ is the cluster-level random intercept, and $\varepsilon_{ij}$ is the individual-level residual error term.

To account for limited range and non-normality in outcome distributions, we used multi-level cluster wild bootstrapping^30^ with an identity link to estimate confidence intervals from 10,000 repetitions. Inference was based on the empirical distribution of wild bootstrap replications, preserving the mixed-effects structure and accounting for within-cluster correlation without relying on asymptotic degrees-of-freedom approximations or parametric covariance structures. Wild bootstrapping was performed with the wild_bootstrap function (lmeresampler package version 0.2.4), which utilises a Mammen weight function with the HC3 consistent covariance estimator.

Treatment effects represent absolute mean changes at endline, conditional on covariates, which reflects movement on Likert scales for caregiver mental health and prevention of violence against women, and change in occurrence in days within the past week for all other outcomes. We used bootstrap repetitions to calculate percentage change based on predicted probabilities of endline outcomes across arms from our primary model, with confidence intervals estimated through bootstrapping. We estimated standardised mean differences to serve as standardised effect sizes (indicated as ‘d’) by dividing treatment effects at endline by standard deviations at baseline. Data cleaning and analysis were performed in *RStudio* (v. 2024.04.2 + 764). Adverse event risk differences were estimated using the Newcombe (Wilson-based hybrid) method. All derivations of outcomes and analyses were duplicated by two analysts (ST and GMT).

As prespecified, given the percentage of observations with missing data was <10% for all outcome models, we used listwise deletion.^18^^,^^19^ Consistent with the prespecified analysis plan, adjustments for multiple comparisons were not made, in accordance with epidemiologic theory.^31^ This results in a Type I error risk of 0.142.

### Interim analyses

Ethical standards for research in active conflict settings requires prioritising the maximisation of intervention benefits, including the timely delivery of interventions with demonstrated effectiveness.^32^ Accordingly, we prespecified a Bayesian interim analysis to inform whether the trial should conclude at endline or continue to a long-term follow-up timepoint, which would require further delaying intervention delivery to the waitlist control arm.

A Bayesian approach was selected to allow incorporation of sceptical priors, which is an established principled approach to enable timely, evidence-based decision-making in trials that is not possible within a frequentist framework. Interim analyses were conducted to inform the timing of intervention delivery to wait-list control clusters. If the intervention demonstrated interim effectiveness that remained robust after incorporation of sceptical priors, the intervention would be delivered to control clusters after the intervention arm’s post-intervention timepoint—rather than extending the trial to a longer-term experimental follow-up. Evidence of effectiveness on any single primary outcome was considered sufficient to justify delivery to the control arm, given the war context and its detrimental impacts on parents and children.

Early stopping for efficacy before trial endline was not considered, as facilitators lacked the capacity to begin delivering Hope Groups to control clusters prior to completing delivery to intervention clusters. Stopping for futility, defined as a low probability of demonstrating benefit, was not considered; the trial was prespecified to continue until the target sample size was reached. Had interim analyses indicated harm, defined as worsening outcomes in the intervention versus control arm, all trial activities would have been paused for investigation.^18^^,^^19^

The interim analysis was conducted by an analyst (ST) using rstanarm (v. 2.32.1) in R after the first 20 clusters completed endline assessments, using the same two-level model specified above, under neutral and sceptical prior specifications on treatment effects.^18^^,^^19^ Full details on the interim analysis procedures, including calculation of sceptical priors, are in the Statistical Appendix, published protocol, and OSF statistical analysis plan.^18^^,^^19^

Interim results were reviewed by the study leads (ST,NB,SH,LC), statistical advisors (OR,SF), and Ukrainian trial advisors. No harms or adverse events were identified. To minimise operational bias, facilitators continued delivering the intervention according to the manual and protocol after interim analyses. No alpha spending adjustments were applied for the interim analysis, as it was conducting using a Bayesian framework which does not use p-values for inference.

### Role of the funding source

No funders were involved in the study design, data collection, analysis, or interpretation. Authors were not precluded from accessing data, and all authors accept responsibility for this publication. GJMT acknowledges the National Institute for Health and Care Research as a Senior Investigator. SF acknowledges the Engineering and Physical Sciences Research Council (EP/V002910/2).

## Results

**Fig. 1:**
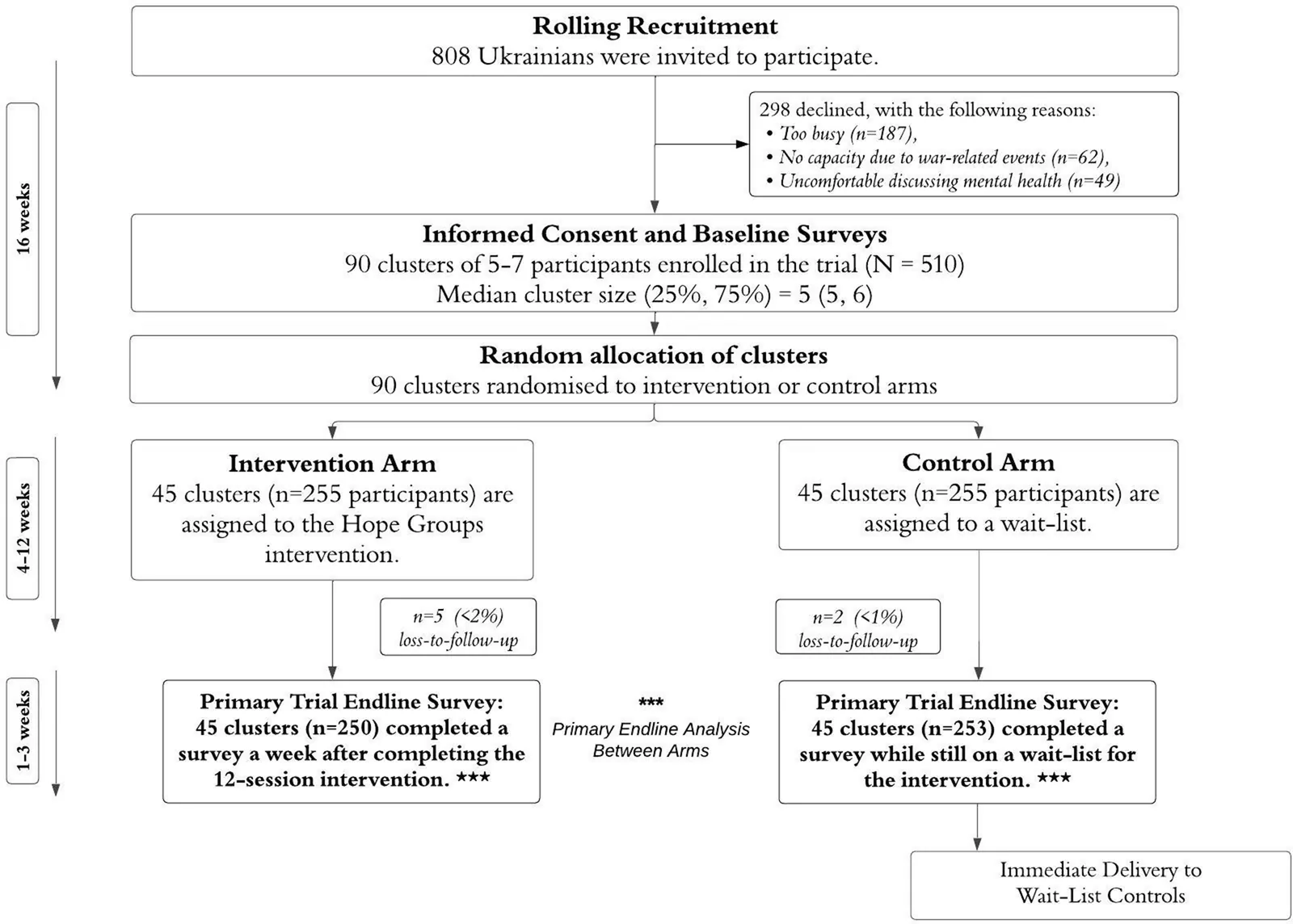
Randomised controlled trial flow chart.

**Fig. 2:**
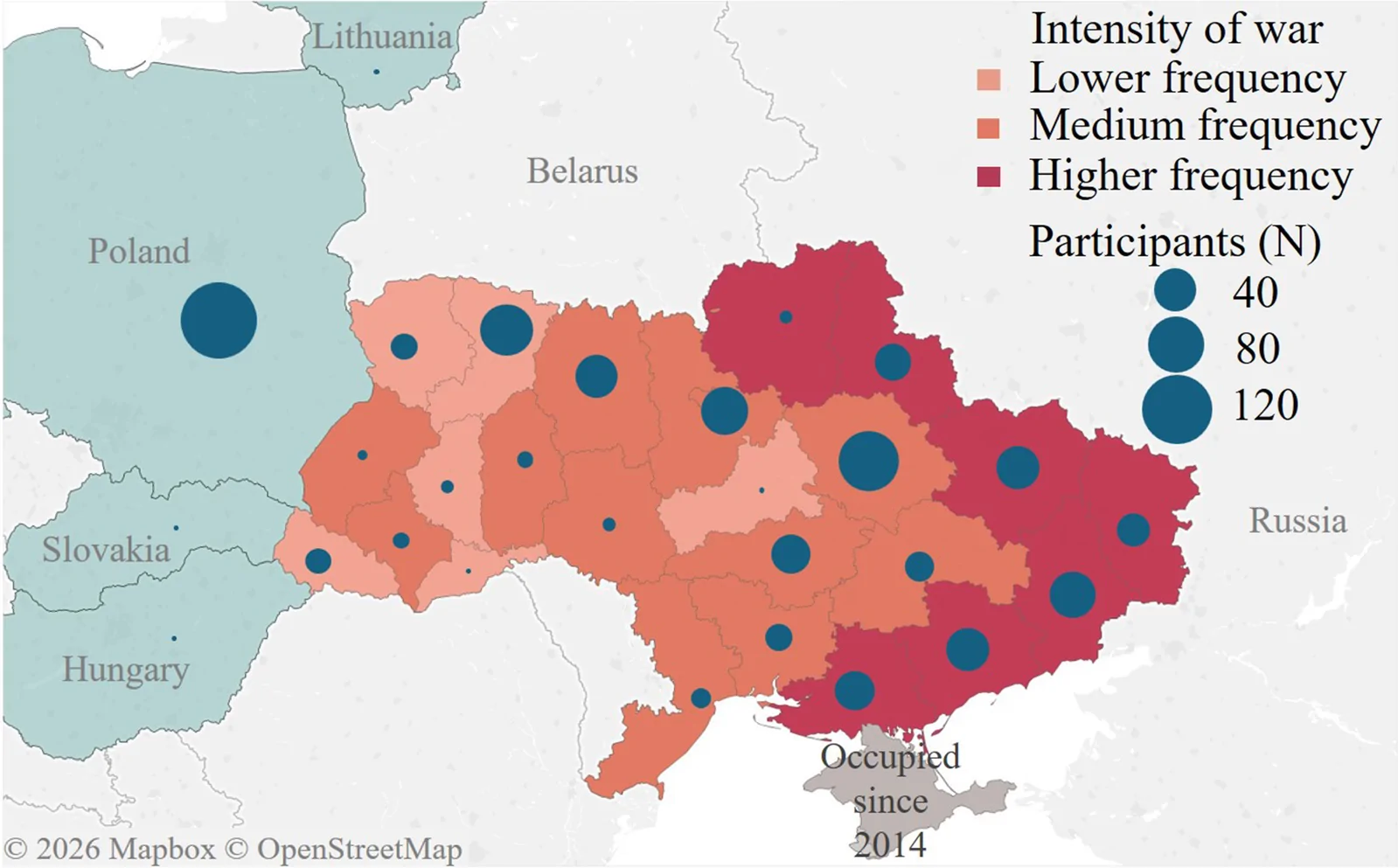
Descriptive location of randomised participants at enrolment, spanning within and outside Ukraine, with intensity of war measured via occupation and shelling by Oblast regions.

In total, 510 participants (91.2% female; 88.6% parents and 11.4% other caregivers) were recruited (Fig. 1), including participants who were internally displaced (27.1%), externally displaced (29.4%), and living at-home in war-affected Ukraine (43.3%) (Fig. 2, Table 1). All participants (100%) were Ukrainian. Participants were located across Ukraine at the time of Russia’s 2022 invasion, with 80.6% residing in areas of higher war-frequency, measured by frequency of shelling events, air raid sirens, and drones (Fig. 2). Participants were recruited from shelters (37%), NGOs/FBOs and churches (26%), and personal networks such as neighbours (23%), or recommendations from past pilot groups (15%). Participants’ children spanned a wide age range: 55.5% had ≥1 child aged ≥ 13; 46.9% aged 8–12; 31.6% aged 4–7; and 23.9% aged 0–3. The prevalence of depression and anxiety among participants was high, with 48.9% and 51.9% meeting thresholds for depressive and anxiety disorders, respectively.

**Table 1:** Baseline characteristics among randomised Ukrainian participants affected by war (2023–2024).

	Control	Intervention	Overall	p-value
	(N = 255)	(N = 255)	(N = 510)	
Displacement status, N (%)				
At-home in war-affected Ukraine	110 (43.1%)	111 (43.5%)	221 (43.3%)	0.64
Externally displaced	71 (27.8%)	79 (31.0%)	150 (29.4%)	
Internally displaced	73 (28.6%)	65 (25.5%)	138 (27.1%)	
Age, N (%)				
Under 20 years	0 (0%)	2 (0.8%)	2 (0.4%)	0.52
20–29 years	29 (11.4%)	24 (9.4%)	53 (10.4%)	
30–39 years	117 (45.9%)	109 (42.7%)	226 (44.3%)	
40–49 years	84 (32.9%)	87 (34.1%)	171 (33.5%)	
50–59 years	14 (5.5%)	21 (8.2%)	35 (6.9%)	
Over 60 years	11 (4.3%)	11 (4.3%)	22 (4.3%)	
Sex (N, % female)	236 (92.5%)	229 (89.8%)	465 (91.2%)	0.34
Marital status, N (%)				
Married	144 (56.5%)	160 (62.7%)	304 (59.6%)	0.51
Unmarried with partner	21 (8.2%)	22 (8.6%)	43 (8.4%)	
Single	32 (12.5%)	29 (11.4%)	61 (12.0%)	
Divorced	39 (15.3%)	32 (12.5%)	71 (13.9%)	
Widowed	19 (7.5%)	12 (4.7%)	31 (6.1%)	
Education, N (%)				
Graduate degree	52 (20.4%)	55 (21.6%)	107 (21.0%)	0.95
Specialist	53 (20.8%)	54 (21.2%)	107 (21.0%)	
Bachelor’s degree	49 (19.2%)	42 (16.5%)	91 (17.8%)	
Technical degree	74 (29.0%)	77 (30.2%)	151 (29.6%)	
Primary school	25 (9.8%)	26 (10.2%)	51 (10.0%)	
Income, N (%)				
High income	5 (2.0%)	7 (2.7%)	12 (2.4%)	0.64
Middle income	110 (43.1%)	101 (39.6%)	211 (41.4%)	
Low income	137 (53.7%)	144 (56.5%)	281 (55.1%)	
Number of children per household, M (SD)	2.00 (1.10)	1.96 (1.18)	1.98 (1.14)	0.67
Country location, N (%)				
Ukraine	184 (72.2%)	176 (69.0%)	360 (70.6%)	0.64
Poland	59 (23.1%)	63 (24.7%)	122 (23.9%)	
Other Country	12 (4.7%)	16 (6.3%)	28 (5.5%)	
Primary outcomes, median (25%, 75%)				
Caregiver mental health	6 (4, 8)	5 (4, 8)	5 (4, 8)	0.68
Parenting practices	3.67 (2.17, 5.33)	3.83 (2.33, 5.50)	3.83 (2.21, 5.46)	0.99
Violence against children	1 (0.5, 2)	1 (0.5, 2)	1 (0.5, 2)	0.34

All participants who agreed to participate were randomised (N = 510), and 98% of randomised participants completed an endline survey (N = 508) (Fig. 1). All outcomes were balanced between arms at baseline (Tables 1 and 2). The trial recruitment occurred from November 2023–February 2024, with trial data collection completing in July 2024 (Fig. 1).

**Table 2:** Intention-to-treat treatment effects of hope groups among ukrainians affected by war at trial endline: after the 12-session intervention (2023–2024).

Type	Outcome	Intervention		Control		N	Absolute treatment effect β (95% CI)	Percent change % (95% CI)	p-value	D
		Baseline M (SD)	Endline M (SD)	Baseline M (SD)	Endline M (SD)					
Primary										
	**Caregiver depression and anxiety**	5.84 (3.15)	2.26 (2.09)	5.99 (3.35)	5.16 (3.14)	496	−2.85 (−3.49, −2.22)^a^	−57.4% (−65.1%, −48.5%)	<0.0001	−0.87
	**Violence against children**	1.23 (1.22)	0.51 (0.66)	1.25 (1.12)	1.02 (1.00)	491	−0.5 (−0.64, −0.36)^b^	−52.6% (−64.7%, −36.5%)	<0.0001	−0.43
	**Parenting practices**	4.04 (2.14)	6.09 (1.23)	4.22 (2.08)	4.73 (1.76)	459	1.32 (0.92, 1.71)	33.07% (24%, 44%)	<0.0001	0.57
Secondary										
	**Caregiver well-being**									
	Hopefulness about the future and coping with grief	2.43 (1.84)	4.95 (1.69)	2.42 (1.89)	3.07 (1.82)	489	1.82 (1.38, 2.25)^b^	65.43% (46.9%, 87.34%)	<0.0001	0.97
	Caregiver self care	2.06 (2.04)	5.16 (1.93)	2.06 (2.22)	2.84 (2.10)	488	2.21 (1.71, 2.71)^b^	84.69% (60.3%, 114.35%)	<0.0001	1.04
	**Child well-being**									
	Child behavioural issues: internalising and externalising behaviours	2.29 (1.67)	0.98 (0.95)	2.25 (1.64)	2.20 (1.47)	477	−1.11 (−1.43, −0.79)^b^	−56.1% (−64.5%, −46.0%)	<0.0001	−0.66
	**Prevention of violence against women**	NA	0.87 (0.23)	NA	0.52 (0.36)	477	2.26 (1.7, 2.82)^a^	68.05% (47.9%, 93.14%)	<0.0001	0.93
(Married or partnered only)	**Healthy relation-ship building**	2.76 (2.48)	5.40 (1.83)	2.88 (2.60)	3.34 (2.26)	322	2.11 (1.59, 2.62)^b^	64.71% (40.4%, 96.21%)	<0.0001	0.83
	**Parenting (subscales decomposed)**									
	Nonviolent discipline	3.65 (2.46)	5.38 (1.92)	3.44 (2.39)	3.84 (2.01)	492	1.45 (1.02, 1.88)^b^	43.33% (28.6%, 60.3%)	<0.0001	0.6
	Positive parenting	3.72 (2.22)	5.67 (1.49)	3.65 (2.15)	4.39 (1.87)	486	1.2 (0.79, 1.61)^b^	31.77% (20.9%, 43.85%)	<0.0001	0.55
	Parental monitoring	4.77 (2.35)	6.33 (1.46)	4.79 (2.41)	5.09 (2.07)	482	1.18 (0.74, 1.62)^b^	25.42% (15.7%, 36.46%)	<0.0001	0.49
	Parental involvement	3.37 (1.96)	5.38 (1.55)	3.10 (1.87)	3.73 (1.72)	485	1.6 (1.21, 1.97)^b^	45.65% (32.1%, 60.49%)	<0.0001	0.87

a Treatment effect indicates change on Likert frequency scale (e.g., ‘not at all’ to ‘nearly every day’).

b Treatment effect indicates change of outcome measured via days of occurrence within the past week (0–7).

Programme fidelity was high: All facilitators delivered all 12 sessions. Across sessions, 91.2% of facilitators completed all content, with the remainder completing “most” or “over half”. Facilitators offered one-on-one catch-up sessions for participants who missed a session. Programme completion among participants was also high: 83% of the intervention arm completed 11 of 12 sessions (N = 211), and 76% completed all 12 sessions (N = 193). Participants who were displaced and living in shelters were more likely to complete all 12 sessions (Supplementary Table S7). In the intervention arm, 48% of participants (N = 122) received in-person delivery, 42% (N = 107) remote delivery, and 10% a hybrid-format (N = 25). Regarding duration, 38% of participants (N = 97) attended sessions twice weekly for six weeks, 34% (N = 87) once weekly for 12 weeks, and 10% three times weekly for four weeks (N = 26).

### Impact of the intervention on primary and secondary outcomes

After completion of the 12-session intervention, we found 57.4% reductions in caregiver depression and anxiety symptoms in the Hope Groups intervention arm compared to the waitlist control arm (β = −2.85; 95% CI = −3.49, −2.22; p < 0.0001; d = −0.87). Similarly, we observed 33.07% stronger parenting practices comparing the intervention arm to the control arm at endline, and 52.7% lower emotional and physical violence against children (β = −0.50; 95% CI = −0.64, −0.36; p < 0.0001; d = −0.43) (Table 2, Supplementary Table S6). With three primary outcomes, a Bonferroni-adjusted threshold would correspond to p < 0.017; statistical inference would be unchanged under this correction. Interim analysis results were consistent with final results (Supplementary Table S4) and informed the decision to deliver Hope Groups to control clusters immediately after trial endline, rather than continuing the trial for an experimental long-term follow-up. There were no adverse events or harms; the risk difference of adverse events in the intervention versus control arm was 0.0 (95% CI = −0.01, 0.01) (Supplementary Table S5).

Among secondary outcomes, caregivers who participated in Hope Groups had 65.4% higher levels of hopefulness about the future and coping with grief at endline (β = 1.82; 95% CI = 1.38, 2.25; p < 0.0001; d = 0.97) and 84.7% higher levels of self-care practices (β = 2.21; 95% CI = 1.71, 2.71; p < 0.0001; d = 1.04), compared to control participants. Parental involvement at endline was 45.7% higher in the intervention versus control arm (β = 1.6; 95% CI = 1.21, 1.97; p < 0.0001; d = 0.87), while parental monitoring was 25.4% higher (β = 1.18; 95% CI = 0.74, 1.62; p < 0.0001; d = 0.49) and positive parenting was 31.8% higher (β = 1.2; 95% CI = 0.79, 1.61; p < 0.0001; d = 0.55). Nonviolent discipline was 43.3% higher in the intervention versus control arm (β = 1.45; 95% CI = 1.02, 1.88; p < 0.0001; d = 0.60). Intervention participants’ children had 56% lower levels of child behavioural issues (β = −1.11; 95% CI = −1.43, −0.79; 56% less; p < 0.0001; d = −0.66) versus control participants’ children, including internalising and externalising symptoms. Prevention of violence against women was 68.05% higher in the intervention versus control arm (β = 2.26, 95% CI = 1.70, 2.82; p < 0.0001; d = 0.93) and healthy relationship practices were 68.05% higher (β = 2.11, 95% CI = 1.59, 2.62; p < 0.0001; d = 0.83) (Table 2). The percentage of observations with missing data was <10% for all outcome models (Supplementary Tables S8 and S9); thus, as pre-specified, we used listwise deletion.

## Discussion

This study is among the first RCTs to evaluate an integrated mental health and parenting intervention during active war, using a delivery format designed for Ukrainian families who were externally displaced, internally displaced, and living at-home in Ukraine. Following the 12-session intervention, medium-to-large intervention effects favoured *Hope Groups* across all primary and secondary outcomes. Caregiver mental health and violence against children were −57.43% and −52.6% lower in the intervention versus control arm, and protective parenting practices were 33.07% higher in the intervention versus control arm. There were positive intervention effects of over 65% for caregiver hopefulness about the future and coping with grief, child behavioural issues, and prevention of violence against women. The results were derived from a facilitator-led delivery model including in-person, hybrid, and virtual modalities to meet the needs of participants facing crisis, achieving high completion rates amidst active war. There were no harms or adverse events.

The observed intervention effects across all four key domains of parental mental health, parenting practices, violence against children, and child well-being contribute to the growing body of evidence suggesting the utility of combining mental health and parenting content within one intervention.^33^ In humanitarian settings, mental health and parenting interventions have shown mixed effects, often improving one or two domains only.^34^ In a systematic review across 10 parenting intervention studies among forcibly displaced families, none of the interventions influenced all measured domains of parental mental health, parenting practices, and child well-being.^34^ In light of this previous research, the positive effects of Hope Groups across all four domains are notable and warrant the prioritisation of similar integrated interventions in crisis settings. The findings are consistent with theoretical foundations of the Caregiver Support Intervention, developed for Syrians affected by war in 2019, emphasising the need for parenting programmes to sufficiently support caregiver mental health to maximise effectiveness.^9^

Importantly, findings from this cRCT also support calls to incorporate virtual and hybrid approaches to enhance programme accessibility and scalability. Although digital mental health interventions have demonstrated effectiveness in humanitarian contexts^35^ and digital parenting programmes in non-humanitarian settings,^36^ evidence on digital or virtual mental health and parenting programmes in conflict settings remains sparse. Results from this cRCT suggest that facilitator-led, video-based virtual/hybrid sessions may be an effective delivery model, particularly in active displacement contexts like Ukraine. Specifically among Ukrainians, our estimates of depressive disorder were consistent with those reported in a cross-sectional study among Ukrainian parents (48.9% versus 46.7%), whereas our estimates of anxiety disorder were higher (51.9% versus 24.2%).^9^ Overall, these cRCT results are consistent with literature beyond humanitarian settings, demonstrating the potential for parenting interventions to improve outcomes among both parents and children.^1^^,^^14^^,^^37^

Several key factors may have contributed to the effectiveness of Hope Groups and this cRCT. First, the intervention directly addressed wartime priorities identified by Ukrainian parents affected by war, including mental health, parenting practices, and social support. Second, the programme content was based on globally-derived evidence from the Global Reference Group for Children Affected by Crisis, particularly parenting content from Parenting for Lifelong Health and trauma-informed mental health content from psychologists with LAMb International. Evidence-based content was adapted in conjunction with Ukrainians for active war and displacement. Third, the community-based, decentralised recruitment model expedited recruitment and contributed to high completion rates rarely observed in other interventions for Ukrainians amidst war.^16^ Recruitment from trusted peer facilitators within their own networks may have overcome potential resistance to participating in a mental health, parenting, and violence prevention intervention. Additionally, this peer facilitator model expanded capacity for intervention delivery in crisis contexts, where mental health care systems are already overburdened—which may provide a blueprint for a promising, scalable, decentralised, locally-led model when centralised structures have weak capacity. Lastly, the trial design prioritised both internal validity and feasible implementation for local partners amidst an active war context, including utilisation of stratified cluster randomisation design to simultaneously address operational considerations raised by Ukrainian stakeholders and balance confounders within recruitment networks.

This study has limitations. First, outcomes relied on caregiver self-report, without corresponding child reports. Surveying children was not acceptable in the war context based on guidance from local partners. To mitigate social desirability bias, surveys were self-administered and pseudonymous. Future studies could additionally include direct observation or child report to strengthen findings. Second, abbreviated scales were used for two of three primary outcomes due to feasibility constraints in the active war setting, rather than full validated scales. Short survey instruments were used to minimise participant burden and support high completion rates in a traumatised population, consistent with guidance for research in humanitarian settings.^28^ While these likely contributed to high response rates (98%), we cannot exclude that abbreviated measures may have introduced measurement bias. Research is needed to develop short, validated scales for active crisis settings. Third, Hope Groups were offered to control clusters after trial endline when interim analyses demonstrated robust intervention effects, precluding an experimental long-term follow-up.^18^^,^^19^ Next steps include designing stepped wedge trials to assess long-term effectiveness. Fourth, participant and facilitator blinding were not possible, which could have introduced bias. Fifth, analyses were conducted without adjustment for multiplicity, inflating the Type I error risk to 0.142 for primary outcomes. However, a Bonferroni adjusted significance threshold for three primary outcomes would be p < 0.017, under which all primary outcomes would remain statistically significant. Lastly, these results are specific to Ukrainian caregivers (91.2% female); future research should explore if intervention effects are generalisable to male caregivers and other war settings.

This scalable delivery modality, along with the design of this cRCT, bring important insights for real-world implementation and research amidst war, displacement, and polycrisis. Using interim analyses may help close the well-documented time delay gap between research and practice.^38^ Only six months after the start of the cRCT, interim results resulted in: 1) securement of programme funding to scale Hope Groups to an additional 600 families within Ukraine; 2) initiation of utilising ADAPT,^39^ a programme adaptation framework, to locally contextualise Hope Groups for crisis settings in the Middle East, North Africa, and Colombia. Especially in crisis settings where delays in evidence-based action are acutely costly, these methods may be promising tools for expediting research-to-action.

In conclusion, this cRCT demonstrates that the Hope Groups intervention resulted in improved caregiver mental health, strengthened parenting practices, and reduced violence against children among Ukrainians, with no harms or adverse events identified. Our next steps include adapting, implementing, and evaluating the intervention in new regions to assess its generalisability across crisis contexts globally.

## Contributors

ST designed the RCT, with expertise from SH, LC, JL, and GJMT. ST led the writing of this manuscript, with revisions from all co-authors and strengthening of the trial design description by JL, LR, and OR. SH, LC, NB, LB, and OHR co-created the Hope Groups intervention and delivery model, with expert support from LS and PG. ST conducted the randomisation. GJMT designed the statistical analysis approach and served as the trial’s senior statistician. OR and SF designed the trial’s interim analysis approach. ST and GJMT both conducted all analyses presented in this manuscript. NB led the coordination of the Hope Groups programme, with key support on programme implementation from JB, OHR, TM, and SK. ST led the trial’s data collection, with monitoring and cleaning support from JB and outcome selection support from IV. KL created the map with war-affected regions.

## Data sharing statement

All session content is open-source, available at http://worldwithoutorphans.org/resources/hope-groups. De-identified data will be made available to researchers upon reasonable request, subject to appropriate data-use conditions. The research team will happily share survey forms and code upon request.

## Editor note

The Lancet Group takes a neutral position with respect to territorial claims in published maps and institutional affiliations.

## Declaration of interests

The authors declare the following financial interests/personal relationships which may be considered as potential competing interests: LC reports financial support was provided by UK Research and Innovation Global Challenges Research Fund and by Oak Foundation. JL is the CEO and LC is the Chairperson of Parenting for Lifelong Health (PLH), a charitable organisation based in the United Kingdom. The Hope Groups programme was developed based on content from PLH, which is open access and licenced under a Creative Commons 4.0 Attribution Share-Alike licence. JL and LC participate in a number of research studies involving PLH programmes as investigators; the University of Oxford and University of Cape Town receive research funding for these. ST is a Clarendon Scholar at the University of Oxford and consults for World Relief and Parenting for Lifelong Health. OR reports financial support provided by the Moderna Foundation. GJMT is a National Institute for Health and Care Research (NIHR) Senior Investigator. OHR serves with NGO Nehemiah in Ukraine, an implementing partner of Hope Groups. All other authors declare that they have no competing interests.

## Appendix A Supplementary data

Supplementary Tables S1–S9

Statistical Appendix

Published Study Protocol

Trial Monitoring

Hope Group RCT Full Statistical Analysis Plan

Collaborator Group

Translated Abstract
