# Supplementary material for: Effectiveness of the hope groups intervention on mental health, parenting practices, and violence against children among Ukrainian parents and caregivers affected by war and displacement: a cluster randomised controlled trial
Source: Lancet Reg Health Eur. 2026 Aug 25;68:101783. doi: 10.1016/j.lanepe.2026.101783 (PMC13559926; doi:10.1016/j.lanepe.2026.101783)
Supplement: Supplementary file 1 [file mmc3.pdf]

Tucker et al. Trials (2024) 25:486
https://doi.org/10.1186/s13063-024-08233-3

Trials

STUDY PROTOCOL Open Access

# Hope Groups: a protocol for a cluster randomized controlled trial of psychosocial, mental health, and parenting support groups for Ukrainian caregivers during war and conflict

Sydney Tucker[1,2*], Nicole Baldonado[3], Olha Ruina[3], Oliver Ratmann[2], Seth Flaxman[4], Lyudmyla Bryn[5], Jamie Lachman[1,6,7], Evgenia Taradaika[3,8], G. J. Melendez-Torres[9], Inge Vallance[1], Philip Goldman[10], Lucie Cluver[1,11†] and Susan Hillis[12†]

## Abstract

**Background** In 2021, more than two-thirds of the world's children lived in a conflict-affected country. In 2022, 13 million Ukrainians were forced to flee their homes after Russia's full-scale invasion. *Hope Groups* are a 12-session psychosocial, mental health, and parenting support intervention designed to strengthen parents, caregivers, and children affected by war and crisis. The primary objective of this study is to evaluate the effectiveness of *Hope Groups* among Ukrainians affected by war, compared to a wait-list control group. This protocol describes a promising decentralized intervention delivery model and an innovative research design, which estimates the causal effect of *Hope Groups* while prioritizing prompt delivery of beneficial services to war-affected participants.

**Methods** This protocol describes a pragmatic cluster randomized controlled trial (RCT) among Ukrainians externally displaced, internally displaced within Ukraine, and living at home in war-affected areas. This study consists of 90 clusters with 4–7 participants per cluster, totaling approximately $n = 450$ participants. Intervention clusters will receive 12-session *Hope Groups* led by peer facilitators, and control clusters will be wait-listed to receive the intervention after the RCT concludes. Clusters will be matched on the facilitator performing recruitment and intervention delivery. Primary outcomes are caregiver mental health, violence against children, and positive parenting practices. Secondary outcomes include prevention of violence against women and caregiver and child well-being. Outcomes will be based on caregiver report and collected at baseline and endline (1-week post-intervention). Follow-up data will be collected among the intervention group at 6–8 weeks post-intervention, with aims for quasi-experimental follow-ups after 6 and 12 months, pending war circumstances and funding. Analyses will utilize matching techniques, Bayesian interim analyses, and multi-level modeling to estimate the causal effect of *Hope Groups* in comparison to wait-list controls.

**Discussion** This study is the first known randomized trial of a psychosocial, mental health, and parenting intervention among Ukrainians affected by war. If results demonstrate effectiveness, *Hope Groups* hold the potential to be adapted and scaled to other populations affected by war and crisis worldwide. Additionally, methodologies described

[†]Lucie Cluver and Susan Hillis are joint senior authors.

*Correspondence:
Sydney Tucker
sydney_tucker@berkeley.edu
Full list of author information is available at the end of the article

© The Author(s) 2024. **Open Access** This article is licensed under a Creative Commons Attribution 4.0 International License, which permits use, sharing, adaptation, distribution and reproduction in any medium or format, as long as you give appropriate credit to the original author(s) and the source, provide a link to the Creative Commons licence, and indicate if changes were made. The images or other third party material in this article are included in the article's Creative Commons licence, unless indicated otherwise in a credit line to the material. If material is not included in the article's Creative Commons licence and your intended use is not permitted by statutory regulation or exceeds the permitted use, you will need to obtain permission directly from the copyright holder. To view a copy of this licence, visit http://creativecommons.org/licenses/by/4.0/. The Creative Commons Public Domain Dedication waiver (http://creativecommons.org/publicdomain/zero/1.0/) applies to the data made available in this article, unless otherwise stated in a credit line to the data.

in this protocol could be utilized in crisis-setting research to simultaneously prioritize the estimation of causal effects and prompt delivery of beneficial interventions to crisis-affected populations.

**Trial registration** This trial was registered on Open Science Framework on November 9, 2023. Registration: OSF.IO/UVJ67.

**Keywords** War, Refugee, Internally displaced person, Psychosocial, Parenting, Mental health, Violence against children, Violence against women, Community-based participatory research, Impact evaluation

## Introduction

### Background and rationale {6a}

Today, over two billion people live in countries affected by conflict, fragility, or violence [1]. Specifically among children, more than two-thirds of the world's children lived in a conflict-affected country in 2021, and more than one in six children lived within 50 km of conflict zones [2]. Globally, an estimated 110 million people are displaced—which is more than ever before in history [3]. Exposure to armed conflict and forced migration leads to short-term and long-term consequences on health and well-being, including mental health distress, post-traumatic stress disorder, complex grief, violence, and exploitation [4, 5]. Furthermore, children are often among the most vulnerable, as adverse childhood experiences are known to have compounding consequences even into adulthood, spanning across all aspects of life, including mental and physical health, educational attainment, stable employment and housing, future incarceration, and future family violence [6]. Research is urgently needed to identify effective, sustainable approaches to strengthen caregiver mental health and parenting practices to protect and nurture children, and prevent traumas such as violence against women and children; yet, research must be conducted utilizing innovative approaches that are flexible and feasible amidst crisis settings, which prioritize prompt delivery of beneficial services to affected populations while yielding robust results.

In Ukraine—the second largest country in Europe [7]—Russia's full-scale invasion forced over 13 million Ukrainians, most of whom are women and children, to flee from their homes during the first year of war [8]. The invasion resulted in mass casualties, separation of families, destruction of homes and schools, and economic upheaval. Almost two of every three Ukrainian children were displaced, creating a generation of children separated from their parents, siblings, homes, and schools [9]. As the war continues, 4.5 million displaced Ukrainian women and children have returned to Ukraine, where $100 billion in infrastructure destruction has led to loss of employment and access to basic needs such as water, gas, and electricity, and missile, rocket, and drone attacks continue as constant threats to survival [10, 11]. Overall, 75% of Ukrainian parents state their children show symptoms of psychological traumatization [12]—making Ukraine an urgent priority setting for identifying effective, sustainable approaches to strengthen caregivers and children amidst war.

Strengthening mental health, parenting skills, and relationships between caregivers and children in times of war is essential for building resilience in both caregivers and children [13, 14]. Caregivers in war zones and refugee communities face not only psychological impacts of exposure to war and loss, but also additional acute stressors from increases in poverty, housing instability, and difficulty protecting and providing for their children [15]; undoubtedly, acute stressors have harmful consequences on parenting practices and children's overall health and well-being [16, 17]. Furthermore, existing evidence has already demonstrated the benefit of mental health and parenting programs on children's development [18], which are of even greater priority amidst war settings as mental health and positive parenting practices decline. However, war-time threats and uncertainties present immense challenges in delivering flexible, contextualized, and scalable mental health, positive parenting, and violence prevention support programs to traumatized caregivers [19].

Therefore, utilizing a community-based participatory research (CBPR) approach [20], Ukrainians displaced by the war, World Without Orphans, Parenting for Lifelong Health (PLH), Oxford University, Children's Mission, Ukraine Without Orphans, and VIVA partnered to design a psychosocial, mental health, and parenting support group to strengthen caregivers and children affected by the war called *Hope Groups*, as well as a flexible, decentralized delivery system utilizing peer facilitators [21], which could be scaled to other crisis-affected contexts. Support group sessions were built based on the expressed needs of Ukrainians who were displaced or living in war-affected regions; all session content was built on evidence-based content from PLH and fully adapted by Ukrainians for a Ukrainian context.

In an initial pre-post study in 2022–2023 [21], *Hope Groups* were evaluated among 577 Ukrainians who were internally displaced, living at home in war-torn regions, or externally displaced across ten countries, who participated in *Hope Groups*implemented by non-governmental

organizations (NGOs), refugee shelters, faith-based organizations (FBOs), and churches; faith-based groups were involved in delivery as 88% of Ukrainians identify as Christians [22]. Compared to baseline, all mental health, parenting, and child health outcomes improved significantly at both midline and endline [21].

Therefore, as a next step after promising pre-post results, we designed an innovative pragmatic cluster randomized controlled trial (RCT), which evaluates the effectiveness of 12-session *Hope Groups* compared to wait-list controls amidst the on-going war in Ukraine. This RCT design, including utilization of matching and Bayesian interim analysis to enable prompt delivery of beneficial services to affected populations, could be adapted and implemented in other research settings amidst war and crisis.

Rolling recruitment began in November of 2023 and is ongoing. This RCT is described using the SPIRIT protocol template and the CONSORT checklist for cluster RCTs [23].

#### Objectives {7}

Our primary objective is to evaluate the causal effect of 12-session *Hope Groups* among Ukrainian parents and caregivers affected by war, compared to a waitlist control group. Primary outcomes include caregiver mental health, violence against children, and positive parenting. Secondary outcomes include additional caregiver and child overall well-being measures and prevention of violence against women. We hypothesize that *Hope Groups* will lead to improvements across all outcomes from baseline to endline.

Further secondary exploratory objectives will include (1) assessing the cost-effectiveness of Hope Groups, considering the system of delivery (in-person, virtual, hybrid); (2) exploring displacement status (internally displaced, externally displaced, at home in conflict zone) as an effect measure modifier of *Hope Groups'* effectiveness; and (3) exploring facilitator background (mental health professional or lay-trained facilitator) as an effect measure modifier of *Hope Groups'* effectiveness.

#### Trial design {8}

We designed a facilitator-matched, cluster RCT with a parallel design and 1:1 allocation ratio to evaluate the effectiveness of *Hope Groups* compared to a wait-list control group, among 90 clusters of 4–7 Ukrainian caregivers internally displaced, externally displaced, and living in war zones. In accordance with our commitment to community-based participatory research while estimating an unbiased causal effect of Hope Groups, clusters are matched on the facilitator who performs both recruitment and facilitation of the intervention, which simultaneously (1) controls for unmeasurable confounders within a facilitator's recruitment network; (2) respects the preferences of participants to join a *Hope Group* with the facilitator who conducts their recruitment; (3) maximizes the logistical constraints of facilitators who can only implement a limited number of *Hope Groups* simultaneously, enabling them to deliver the intervention to their intervention clusters then transition to delivery to control clusters as soon as feasible afterwards, pending findings of the interim analysis; and (4) respects the preferences of facilitators, who requested continuous involvement during delivery to both treatment groups, as they reported gaining personal strength and healing from involvement.

## Methods: participants, interventions, and outcomes

#### Study setting {9}

We will recruit 30 facilitators from NGOs, refugee shelters, FBOs, and churches who participated in the initial *Hope Groups* pre-post study [21], who are living in Ukraine, Poland, Spain, and the UK. Participants will be primarily located in these four countries—with the vast majority in Ukraine and Poland—while participants joining virtually may be displaced throughout Europe and beyond. In-person *Hope Groups* will be held at shelters, community centers, churches, and homes. We recognize that participants may move to different countries throughout the study, and there may also be weeks when it is unsafe to meet in person (e.g., during ongoing air strikes). We have prioritized a pragmatic and flexible delivery system which can transition between meeting virtually and in person as needed.

#### Eligibility criteria {10}

Participant inclusion criteria are as follows: participant (1) is Ukrainian, or another nationality and affected by the war in Ukraine; (2) understands the Ukrainian language; (3) is aged 18 or older; and (4) is a parent or caregiver for one or more children ages 0–17 years old. Participant exclusion criteria are as follows: (1) participants are not eligible if they have already participated in a *Hope Group*.

Facilitator inclusion criteria are as follows: facilitator (1) is Ukrainian, or another nationality and affected by the war in Ukraine; (2) speaks and understands Ukrainian; (3) is 18 or older; (4) is connected to an NGO, refugee shelter, FBO, church, or other networks of Ukrainians; (5) has had previous experience leading small group discussions; (6) self-defines as emotionally

stable with the capacity to support others; (7) is willing to participate in *Hope Groups* trainings; and (8) is willing to recruit and facilitate two or four *Hope Groups* through their network, with the understanding that their groups will be randomized to intervention or control with a 1:1 allocation ratio. There are no inclusion or exclusion criteria for facilitators' backgrounds; some facilitators are mental health professionals, while others have no previous mental health training.

#### Who will take informed consent? {26a}

Facilitators and participants will consent to participation in the study. Facilitators and participants will both complete self-administered informed consent using their phones via a link to an Open Data Kit (ODK) form.

#### Additional consent provisions for collection and use of participant data and biological specimens {26b}

In informed consent, participants consent to making publicly available anonymized data and results to contribute to research identifying ways to strengthen caregivers amidst war. No biological specimens will be collected.

### *Interventions*

#### Explanation for the choice of comparators {6b}

This study compares intervention clusters to waitlist control clusters. Facilitators recruit two or more *Hope Groups*. Logistically, facilitators could not implement all sessions simultaneously; thus, we relied on a random lottery system to select which clusters receive the intervention now and which are assigned to a waitlist to receive the intervention after the RCT concludes. Through utilizing a waitlist design, we expect that placebo effects will be effectively balanced between groups, as the control group's knowledge of soon receiving a supportive intervention may yield improvements in their outcomes [24].

#### Intervention description {11a}

Intervention clusters receive the 12-session psychosocial, mental health, and parenting intervention called *Hope Groups* through trained facilitators (Table 1). All content is based on evidence-based principles and adapted by Ukrainians for the Ukrainian context. Mental health content utilizes key principles for psychosocial support in armed conflict to build participants' skills in healthy grieving and coping, de-escalation and stress reduction, and self-care to address war-time challenges. Parenting and violence prevention content was based on content developed by Parenting for Lifelong Health (PLH) and endorsed by the World Health Organization (WHO) and the United Nations Children's Fund (UNICEF). Additionally, content was informed by the "Ukraine Parenting" resources developed by the Global Initiative to Support Parents, WHO, PLH, UNICEF, United Nations High Commissioner for Refugees (UNHCR), United Nations Office on Drugs and Crime, Global Partnership to End Violence Against Children, and Early Childhood Development Action Network.

Facilitators receive training on all 12 sessions, as well as overall training in supporting individuals in crisis, group facilitation, mental health referrals, mandatory child abuse reporting protocols, gender-based violence, violence against children, and research principles such as reducing spillover and loss-to-follow-up. Total facilitator training time is 14 h, with training sessions delivered in increments of 2 to 3 h at a time.

*Hope Groups* are generally implemented two times per week for 6 weeks; however, given the war crisis and based on the needs of participants, facilitators can elect to implement the *Hope Groups* three times per week for 4 weeks (e.g., particularly in shelter settings where participants are available for frequent meeting and may only remain in this setting for 4 weeks) or one time per week for 12 weeks (e.g., in settings where participants' schedules do not allow more frequent meeting). This pragmatic delivery, which flexibly caters to the needs of a population living amidst a war, was consideredessential [25].

#### Criteria for discontinuing or modifying allocated interventions {11b}

All participants are informed that they may stop participating in *Hope Groups* at any time, and all facilitators are trained to be flexible and adaptive based on the needs of the participants (e.g., pausing content within sessions to let participants share their personal struggles as needed, guiding the conversation to protect all participants from secondary trauma, and making appropriate mental health referrals as needed).

#### Strategies to improve adherence to interventions {11c}

We created a facilitator manual to guide all 12 sessions, including content delivery, skills-building, and discussions. After sessions, we collect monitoring data from facilitators to explore whether all session content from the *Hope Groups* guide was completed. This study relies on a pragmatic design; facilitators are trained to deliver all session content, but we do not additionally intervene to ensure the *Hope Groups* guide is closely followed.

#### Relevant concomitant care permitted or prohibited during the trial {11d}

We have no restrictions on participants receiving other mental health, psychosocial, and parenting support during our study. We collect data on participation in other similar programs and will include this as a covariate in our models if prevalence is high in our sample.

**Table 1** Description of 12-session *Hope Groups* intervention

| Session | Title | Content |
|---|---|---|
| 1 | **Finding stability—our everyday tools** | This session builds skills on strategies for building hope in transition and crisis; we create discussion around the impacts of trauma and equip participants to begin building a personal "toolbox" for responding to crisis. |
| 2 | **Finding stability—our anchoring tools** | This session focuses on common physical, emotional, behavioral, and cognitive reactions to trauma. We build participant skills in developing stabilization techniques, such as deep breathing and progressive muscle relaxation, for coping with stress. |
| 3 | **Talk about it** | This session builds skills in identifying and sharing emotions participants are experiencing related to war-time and other personal crisis events. We build skills in how to talk with children about war and loss. |
| 4 | **Strong families** | This session builds skills in developing and maintaining strong relationships within families, particularly caregiver/child relationships. Skills are built for positive communication within relationships. |
| 5 | **Staying safe together** | This session builds skills and tools for staying safe during crises and war, including being physically together as a family, when possible, and awareness of the risks and signs of human trafficking. We provide a guide for caregivers to learn how to talk with their children about human trafficking in age-appropriate terms, and we equip caregivers to safeguard their children's childhood even amidst crisis. |
| 6 | **Staying safe at home** | This session builds skills to respond to strong emotions (such as anger) in healthy, safe ways—even in stressful moments. We discuss the positive discipline of children. |
| 7 | **Coping with loss** | This session teaches participants about the stages of grief, with an aim to normalize and remove stigma from the concept of grieving. Participants build skills for healthy grieving, finding meaning in their new reality, and helping their children honor whom/what they lost. |
| 8 | **Building hope through understanding guilt and secondary trauma** | This session equips participants to understand "survivor guilt" and "secondary trauma." Facilitators validate the stress of helping others in crisis and help participants build resilience amidst crises. |
| 9 | **Learning together** | This session builds skills for caregivers to support their children's learning, amidst the war crisis, when many children have left their former schools and are attending new schools (even in a foreign language) or are studying at home/online without a traditional education. environment |
| 10 | **Healthy relationships** | This session builds skills for maintaining relationships with loved ones long-distance and making healthy sexual decisions. We clarify the differences between healthy and unhealthy relationships, especially during times of stress and physical separation. |
| 11 | **Staying safe in our relationships** | This session builds skills in saying "No" to unwanted sexual activity. We discuss sexual consent, options for protecting against sexually transmitted infections and unwanted pregnancy, and verbal and physical skills to say "no." We briefly explore how post-traumatic stress. disorder (PTSD) among active military personnel and veterans can lead to vulnerabilities in relationships and safety |
| 12 | **Resilience** | This final session builds skills in exploring emotions and discerning how to continually care for their own mental health, as well as their children's, as they build hope for the future amidst the war crisis. |

### Provisions for post-trial care {30}

Currently, we have no structured plans for continued care after the completion of *Hope Groups*; however, in the pre-post study, we observed some facilitators and participants self-elected to maintain contact after the completion of *Hope Groups*. After the follow-up survey, we will assess the frequency of self-elected continued contact and change in participant outcomes from endline to follow-up, which may inform future adaptations for structured follow-up contact between facilitators and participants.

### Outcomes {12}

Following guidance for research in war settings, researchers made every effort to create a highly sensitive, non-triggering, and brief survey for participants [25]. To achieve this, we adapted validated scales to be relevant to our context, often including relevant subscale items rather than full survey scales. Primary and secondary outcomes are detailed in Table 2; all outcomes are based on participant self-report. Other than Caregiver Mental Health (which uses the standard Patient Health Questionnaire-4 scoring), outcomes are adapted to ask for frequency of occurrence in the past 7 days. All outcomes are collected at baseline, endline, and follow-up, except for Violence Against Women outcomes, which will only be asked at endline and follow-up due to their highly sensitive nature. We also collect data on standard covariates such as age, sex, education, income, and other exploratory outcomes including oblast location at the onset of full-scale invasion, displacement status, military service, facilitator background (lay-trained or mental health professional), and mode of intervention delivery (in-person, virtual, hybrid).

**Table 2** Outcome measures

| Outcome | Specific constructs measured | Sources of survey items |
|---|---|---|
| **Primary outcomes** | | |
| **Caregiver mental health** | **Depression and anxiety** | Patient Health Questionnaire-4 [26] |
| **Violence against children** | **Physical violence**<br>**Emotional violence**<br>**Nonviolent discipline** | International Society for the Prevention of Child Abuse and Neglect Screening Tool Trial Caregiver Version—Physical Abuse and Emotional Abuse subscales [27]; Parenting Young Children—Setting Limits subscale [28] |
| **Positive parenting** | **Monitoring and protecting child**<br>**Supporting child development through play, learning, and reinforcing positive behavior** | Alabama Parenting Questionnaire—Positive Parenting and Parental Involvement subscales [29]; Parenting Young Children [28] |
| **Secondary outcomes** | | |
| **Caregiver well-being** | **Coping with grief, self-care practices, and hopefulness about the future** | Center for Epidemiological Studies Depression [30] |
| **Child well-being** | **Despondency, verbalizing emotions, and internalizing and externalizing behaviors** | Parent Child Communication Scale [31]; Child and Adolescent Behavior Inventory—Internalizing and Externalizing Symptoms subscale [32] |
| **Violence against women** | **Prevention of sexual violence and intimate partner violence** | Adapted from Key Concepts in No Means No Worldwide [33] |

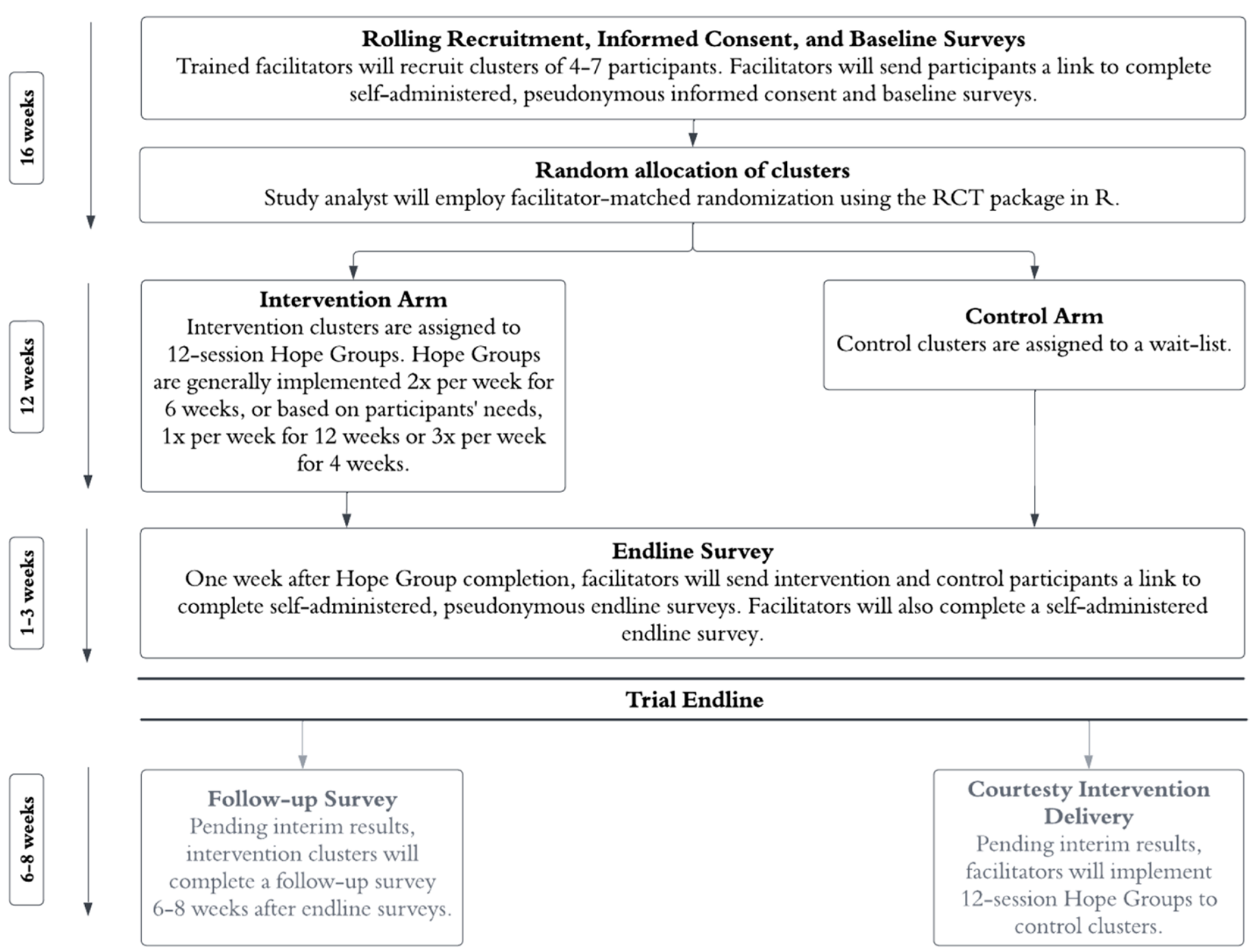


**Fig. 1** *Hope Groups* randomized controlled trial flow chart: procedures and timeline

## Participant timeline {13}

Trial endline will be reached approximately 31 weeks after the RCT's launch (Figs. 1 and 2). Trial endline is 1 week post-intervention. We originally planned to continue the trial through a 6-week follow-up period; however, as missiles and bombings in Ukraine have intensified, Ukrainian partners expressed urgency to deliver the Hope Groups to control clusters immediately after trial endline, rather than delaying control clusters throughout a follow-up period, based on (1) the war context having detrimental impacts on caregiver mental health and violence against children, which results in an urgent need for psychosocial, parenting support; (2) strong results from our pre-post study [21]; and (3) concern that high mobility during the war crisis may result in control participants'

| | STUDY PERIOD | | | | |
|---|---|---|---|---|---|
| | Enrollment | Allocation | Post-allocation | | |
| **TIMEPOINT**** | $-t_1$ | 0 | $t_1$ | $t_2$ | $t_3$ |
| **ENROLLMENT:** | | | | | |
| **Eligibility screen** | X | | | | |
| **Informed consent** | X | | | | |
| **Baseline Survey** | X | | | | |
| **Randomization** | | X | | | |
| **INTERVENTIONS:** | | | | | |
| ***Hope Groups (intervention arm)*** | | | X | | |
| ***Wait-list (control arm)*** | | | X | | |
| **ASSESSMENTS:** | | | | | |
| ***Demographic Information*** | X | | | | |
| ***Primary Outcomes*** | X | | | X | X* |
| ***Secondary Outcomes*** | X | | | X | X* |
| ***Time varying Covariates (e.g., war exposure)*** | X | | | X | X* |

- t1 represents intervention period; t2 represents the endline survey timepoint; and t3 represents the follow-up survey timepoint.

** indicates that the listed activity occurs only among the intervention group.*

**Fig. 2** SPIRIT timeline figure. *t*1 represents the intervention period; *t*2 represents the endline survey timepoint; and *t*3 represents the follow-up survey timepoint. The asterisk (*) indicates that the listed activity occurs only among the intervention group

inability to participate if delayed through a follow-up period. This was recognized and adopted by all study investigators, who designed a plan to conduct an interim analysis after the first 20 clusters (~100 participants) reached endline to provide real-time data-based information to inform Hope Groups delivery to control clusters, with the following guidelines:

1. If we find evidence of a harmful effect across primary outcomes, we will pause all activity to investigate and make adaptations.
2. If we find evidence of a protective effect of *Hope Groups* across primary outcomes, even after incorporation of skeptical priors [34–36], this will be used to support the decision to end the trial after 1-week post-intervention and immediately deliver *Hope Groups* to control clusters (without delaying them through a follow-up period).
3. If we find evidence of a null effect, the trial will continue as planned through endline to enable the sample size to reach the target number. Additionally, we will incorporate enthusiastic priors [34, 35] based on information from the pre-post study [19]; if results remain null, we will still continue the trial until endline, but we will also collaborate with Ukrainian partners to make adaptations to *Hope Groups* prior to delivery to control clusters with the aim of strengthening the intervention. Adapted *Hope Groups* among control clusters would be assessed using a pre-post design.

Furthermore, if we find evidence of a protective effect, study investigators will design a statistical analysis plan to assess the long-term effectiveness of Hope Groups utilizing innovative approaches, as our RCT control group

will have received the intervention and therefore will no longer be an appropriate comparison group. First, we will conduct a follow-up survey only among the intervention arm 6–8 weeks post-endline to assess if outcomes are receding towards baseline levels or maintaining improvement.

Second, to assess the long-term effectiveness of Hope Groups, researchers will aim to conduct 6- and 12-month quasi-experimental follow-ups, pending war circumstances and funding. Building on recent research highlighting the potential for longitudinal matching methods to yield results consistent with long-term RCT results [37], we intend to utilize multiple quasi-experimental methods, including rolling entry matching [38], a longitudinal matching method which improves exchangeability when relying on randomization is not feasible or ethical, to estimate the long-term effects of Hope Groups. Following this design, treated participants from the RCT's intervention group will be matched with untreated participants' baseline surveys upon their enrollment in future *Hope Groups* programming, at the same 6 and 12 months post-RCT timepoints. A separate statistical analysis plan will be registered for any long-term evaluations on OSF.

#### Sample size {14}
This study will enroll $k=90$ clusters of 4–7 participants, totaling approximately $n=450$ participants (assuming an average cluster size of $m=5$). The sample size was determined by power calculations from the pilot study and funding limitations. Based on estimated effect sizes, ICCs, and SDs of the outcome in the population from pilot data, and accounting for attrition in an active war context, we estimated that $k=90$ clusters and $n=450$ participants would be sufficient to detect significant effects with 80% power in all primary and secondary outcomes, except for physical abuse, which we may not be powered to detect an effect in. However, our funding limits increasing the sample size beyond this. Our final sample size may vary based on the average cluster size.

#### Recruitment {15}
There will be a rolling recruitment and enrollment strategy, with recruitment efforts ending when all facilitators have recruited the full number of participants for their groups. *Hope Groups* facilitators will recruit participants through their own networks (e.g., NGOs, shelters for internally displaced persons (IDPs) and/or refugees, FBOs, or churches) to participate in their 12-session *Hope Groups*. This approach to recruitment is based on the guidance of Ukrainian partners and humanitarian research experts [25]. It is especially important in humanitarian contexts for people to know and trust the organizations and people recruiting them for resources, especially in Ukraine, where psychosocial programming is historically uncommon. Therefore, to successfully achieve the target sample size, it is essential for participants to be recruited by someone they know and trust. Because of this programmatic necessity, our study design is matched to facilitator, to ensure all confounders within a facilitator's recruitment network are balanced between study arms.

### Assignment of interventions: allocation
#### Sequence generation {16a}
Cluster randomization matched on the facilitator is employed using the treatment_assign function within the RCT package (v. 1.1.2) in R.

#### Concealment mechanism {16b}
Randomization does not occur until a facilitator has completed recruitment for all clusters. All facilitator-matched clusters are randomized together—which ensures allocation concealment is maintained.

#### Implementation {16c}
Facilitators recruit and enroll participants. ST, who is blind to participant identities, randomizes clusters to treatment or control within each facilitator strata with a 1:1 allocation ratio. Randomization is performed during virtual randomization events with facilitators over Zoom to demystify the research process, increase participation in research, and assure facilitators are aware that assignments are produced randomly and not selected by researchers.

### Assignment of interventions: blinding
#### Who will be blinded {17a}
Blinding for participants is not possible, as the intervention group participates in a *Hope Group*, while the control group will be told they are on a waitlist to receive *Hope Groups* in 2024 (after trial endline). Participants are notified of their allocation status after baseline data collection. Blinding will not be possible for facilitators and research staff after baseline data collection due to their involvement in program implementation. Data analysts will be blinded from condition assignment during all analyses.

#### Procedure for unblinding if needed {17b}
Analysts will not be unblinded during analysis.

### Data collection and management
#### Plans for assessment and collection of outcomes {18a}
To respect participant preferences for privacy and prevent social desirability bias, all participant data is completed through self-administered, pseudonymous online surveys on the Open Data Kit (ODK) using

participant devices. All surveys were piloted with Ukrainians affected by war prior to launch. Basic monitoring data collected from facilitators is also completed through self-administered ODK forms on facilitators' personal devices.

### Plans to promote participant retention and complete follow-up {18b}

To increase participant retention, we offer a small reimbursement equivalent to 5 USD for the completion of the endline and follow-up survey, which equates to approximately 180 Ukrainian hryvnias and would cover the cost of one lunch.

### Data management {19}

We do not have any paper-based consent forms, surveys, or datasets; all data is collected electronically on participant's personal devices using Open Data Kit (ODK) online. Data is stored in ODK, which requires access and a password to enter. Data downloaded from ODK is stored on SharePoint, which is password-protected, and not stored on personal computers. At the end of the study, data will be fully de-identified and made publicly available.

### Confidentiality {27}

At the start of the pre-post study, Ukrainian participants expressed a strong preference to not provide fully identifying information (such as full names). Therefore, we do not collect any identifying information beyond what is sufficient for a unique ID (facilitator name, first initial, last initial, birth month, and birth year). All waves of data collected on participants are linked with this unique ID. All information the participants share within *Hope Groups* sessions is confidential, except if mandated reporting is essential for safety (e.g., abuse of a child or incapacitated adult).

### Plans for collection, laboratory evaluation, and storage of biological specimens for genetic or molecular analysis in this trial/future use {33}

Not applicable. This study will not collect, evaluate, or store any biological specimens.

## Statistical methods

### Statistical methods for primary and secondary outcomes {20a}

Quantitative data will be cleaned and analyzed in RStudio. The primary method of analysis will be a two-level generalized linear mixed-effects model, which will nest person within cluster and control for baseline outcome measures. To account for matching, we include a fixed effect on the multi-categorical variable for the facilitator. We will use an intention-to-treat approach with a binary exposure variable indicating if the participant was randomized to the intervention or control group. We will additionally specify a vector of covariates including baseline score of the outcome and any other important covariates identified as relevant before the conclusion of follow-up data collection and described in the statistical analysis plan. All covariates will be centered at the sample mean. The regression link function will be based on consideration of outcome distributions, and the appropriate link function will be used for each outcome. Where an ideal link function is not available (e.g., limited range variables where linear, Poisson, or ordered logit links are inappropriate), we will use an appropriate bootstrapping method with an identity link. We will use two-tailed tests for all models with statistical significance thresholds of 0.05. Where variables are continuous and approximately normally distributed or where models are bootstrapped, mean differences will be estimated. Where variables are Poisson-distributed, incidence rate ratios (IRRs), which represent the ratio of the incidence rate or frequency of the outcome in one group compared to another group, will be estimated. Where outcomes are binary or ordinal, odds ratios will be estimated. In the event that mixed-effects models are not estimable or do not converge, we will use standard errors clustered at the facilitator group.

Analysis of endline data in the intervention group only will use a three-level model with measurement wave within person within group. To assess the stability of change over time, we will use a "time-differenced" approach with time terms corresponding from baseline to follow-up and from follow-up to endline.

### Interim analysis {21b}

The wartime situation makes it a priority to translate trial findings into practice; for this reason, analysis methods need to incorporate continuous interim analyses for efficacy, and such continuous assessments are seamlessly straightforward within a Bayesian analysis framework [39, 40]. Specifically for the interim analysis, we will use rstanarm (v. 2.32.1) in R to implement our primary and secondary statistical model reported above using a Bayesian approach. We will only assess primary outcomes, which were also included in the pre-post study, and thus we have estimates of the treatment effect and variance to guide a conservative approach to interim assessment of efficacy. The steps are as follows:

1. We will first utilize a weakly informative prior on the treatment effect centered at 0 with a standard deviation of 2.5 (variance of 6.25) to assess if *Hope Groups* demonstrate a significant effect across outcomes indicated above, as measured by quantifying if posterior credible intervals of the treatment effect include the null with a probability of less than 5%.

To conduct interim analyses conservatively, if positive intervention effects are detected, we will incorporate a skeptical prior on the treatment effect, which is centered at 0 with substantially stronger precision than under point (1) above to assert a skeptical belief about the intervention having an effect [35] and assess if the significant effect(s) remain. This approach ensures that evidence needs to be overwhelming for the treatment effect to be called significant in interim analyses. To calculate the skeptical prior, we use the following formula [34, 36]:

$$\text{prior} : f(\theta) \sim \mathcal{N}\left(0, \sigma^2/m_0\right)$$

$$m_0 = (Z_\gamma \sigma/\theta_s)^2$$

where $Z_\gamma$ is the upper quantile of the standard normal (1.645 at $\alpha$= 0.05), $\sigma$ is the standard deviation, and $\theta_s$ is the estimated treatment effect.

2. In our pre-post study [21], the largest standard deviation across primary outcomes was 2.85; to be conservative, we round this to 3.0. The smallest treatment effect across primary outcomes in the pre-post study was 1.13 [21]; to be conservative and assume a 10–15% placebo effect from the pre-post study, we reduce this to 1.0—which also corresponds to the minimum treatment effect Ukrainian partners stated would make the intervention worth implementing. Thus, $m_o$=24.35. Our final skeptical prior is:

$$\text{prior} : \ f(\theta) \ \sim N \ (0, \ 0.37)$$

i.e., a normal distribution with a variance of 0.37 (equivalently a standard deviation of .61).

If any mental health, positive parenting, or violence against children outcomes remain significant after incorporation of the skeptical prior, we will consider there to be sufficient evidence to support providing *Hope Groups* to the control group immediately after trial endline without delaying them through a follow-up period, in light of the war context having detrimental impacts on caregiver mental health, parenting, and violence against children, and especially due to high mobility in populations which may result in participants' inability to participate if delayed through a follow-up period. We select mental health, positive parenting, and/or violence against children outcomes due to their potential for acute impact on both the short-term and long-term health of caregivers and children.

#### Methods for additional analyses (subgroup analyses) {20b}

We will explore differences in the effectiveness of *Hope Groups* among participants who are internally displaced, externally displaced, and living at home, through interacting displacement status with the treatment variable within primary outcome generalized linear mixed-effects models, as well as building stratified models. We will use these same methods to additionally explore differences in the effectiveness of *Hope Groups* among participants who participate virtually or in person and among participants with facilitators who are lay-trained or mental health professionals.

#### Methods in analysis to handle protocol non-adherence and any statistical methods to handle missing data {20c}

If there is significant dropout or non-adherence, we will conduct both per-protocol analysis and instrumental variable analysis for exploration, and we will present these results with ITT results. If any outcome variable missingness is over 10%, we will use multiple imputation for outcome variables where scale scores cannot be rescaled, using a two-level multilevel model without accounting for pair matching.

#### Plans to give access to the full protocol, participant-level data, and statistical code {31c}

This protocol, anonymized data, and R code for monitoring, cleaning, and analysis will all be made publicly available on OSF.

## Oversight and monitoring

#### Composition of the coordinating center and trial steering committee {5d}

The coordinating center at the University of Oxford consists of research, academic, and program staff from both Oxford and World Without Orphans, who meet weekly to monitor and implement the RCT. Additionally, the trial is advised and steered by a group of experts consisting of: Ukrainian psychologists, Ukrainians displaced by the war, statisticians from Imperial College London and Oxford University, and experts in humanitarian contexts and violence against children from the Global Reference Group for Children Affected by Crisis.

#### Composition of the data monitoring committee, its role, and reporting structure {21a}

Given that we do not expect adverse outcomes with this study, we did not compose a separate data monitoring committee.

#### Adverse event reporting and harms {22}

Any adverse events (such as *Hope Groups* content triggering a participant or abuse reported within *Hope Groups*) will be immediately reported by study facilitators to our lead study coordinator, NB, who reports to all investigators. Within 24 h, participants will be referred to

the appropriate resource for support, and necessary mandated reporting will occur. Any adverse events will be reported to ethics committees at Oxford University and the Ukrainian Institute on Public Health Policy.

#### Frequency and plans for auditing trial conduct {23}

Our lead study coordinator, NB, has weekly contact with five coordinators who manage all 30 facilitators to monitor if all trial procedures are carried out according to our protocol.

#### Plans for communicating important protocol amendments to relevant parties (e.g., trial participants, ethical committees) {25}

Any amendments to the protocol will be reported to ethics committees at Oxford University and the Ukrainian Institute on Public Health Policy, as well as reported updates to our OSF trial registration.

#### Dissemination plans {31a}

Our team is highly committed to the broad dissemination of results. We will disseminate results through conference abstracts, peer-reviewed publications, policy briefs, and an online webinar hosted by the Global Reference Group for Children Affected by Crisis. The webinar will be offered to over 100 Ukrainian NGOs, Global Parenting Initiative, 40 World Without Orphans directors with teams serving in crisis settings globally, WHO, UNICEF, UNHCR, The Interagency Task Team for Child Protection in Humanitarian Crises, and the Accelerate Hub at Oxford University.

## Discussion

This study is the first known randomized controlled trial of psychosocial, mental health, and parenting intervention among Ukrainians affected by war, after Russia's full-scale invasion. As Russia's ongoing war in Ukraine has led to the displacement of millions of Ukrainians throughout Europe and beyond, identifying scalable interventions to strengthen and build resilience among Ukrainian caregivers and children is an urgent priority. WHO and Ukraine are prioritizing mental health and psychosocial support during and after the war [41], and if RCT results demonstrate consistent effectiveness as was found in the pre-post study [21], *Hope Groups* could be a scalable solution for psychosocial and mental health support.

Additionally, this RCT pilots innovative approaches to conducting randomized trials in crisis settings, where simultaneous commitment to rigorous research to estimate unbiased causal effects and prioritization of immediate delivery of interventions with the potential to strengthen crisis-affected populations is critical. This RCT utilizes a facilitator-matched design to respect the preferences of facilitators and participants while balancing potential confounders; Bayesian interim analyses to assess the benefit of the intervention in real-time to inform expedited delivery of beneficial interventions to crisis-affected populations; and creative quasi-experimental methods for long-term RCT follow-up. These methods may hold potential to serve as valuable tools for prioritizing prompt delivery of beneficial services to affected populations while conducting rigorous research amidst war and crisis settings.

Crucially, this study uses a pragmatic design to increase external validity in real-world settings. *Hope Groups* are implemented through a sustainable delivery system of both mental health professionals and a wide array of lay-trained NGO, FBO, refugee shelters, and religious organization workers—which could be replicated in other crisis settings. This study has the potential for substantial scientific, programming, and policy benefit—as over two billion individuals and two-thirds of the world's children are living in war-affected countries. Results from this study hold significant potential for adapting and scaling *Hope Groups* to war and crisis-affected populations worldwide, where psychosocial, mental health, and parenting support will be urgently needed. As polycrisis linked to contagion, climate, and conflict escalate and coalesce, the time to identify effective and scalable solutions for restoring and multiplying hope to caregivers and children in crisis is now.

## Trial status

This protocol (version 1.3) was finalized on February 12, 2024. At the time of this submission on February 14, 2024, trial recruitment is ongoing. Recruitment began in November of 2023, and 80 total clusters have been recruited and randomized (89% of our target number of clusters). This fast-paced recruitment—despite facilitators and participants either living in active war settings or being displaced to unfamiliar areas—is indicative of the need and demand for psychosocial, mental health, and parenting programming. We expect to enroll our full sample size in March 2024 and complete endline data collection in May 2024.

#### Abbreviations

| | |
|---|---|
| NGO | Non-governmental organization |
| FBO | Faith-based organization |
| PLH | Parenting for Lifelong Health |
| WHO | World Health Organization |
| RCT | Randomized controlled trial |
| ODK | Open Data Kit |
| CBPR | Community-based participatory research |
| UNHCR | United Nations High Commissioner for Refugees |
| UNICEF | United Nations International Children's Emergency Fund |
| OSF | Open Science Framework |

## Supplementary Information

The online version contains supplementary material available at https://doi.org/10.1186/s13063-024-08233-3.

Supplementary Material 1.


### Acknowledgements

We thank Dr. Sandra McCoy and Dr. Jack Colford from the University of California, Berkeley's Division of Epidemiology, for providing critical consultations on our data collection and monitoring procedures and matched study design.


### Authors' contributions {31b}

ST, SH, and LC designed this RCT to evaluate the effectiveness of *Hope Groups*, and ST drafted the full protocol and this manuscript. SH, NB, OR, and LB developed the *Hope Groups* intervention based on content from Parenting for Lifelong Health, developed by LC, JML, and LS. SH, NB, LC, OR, LB, ST, and LS designed, led, analyzed, and/or advised the pre-post study that preceded this RCT. NB additionally coordinates this RCT and leads the *Hope Groups* program, with expert guidance from OR. JML, GMT, IV, SF, and OR provided methodological, statistical, and coordinating expertise in the designing of this RCT, with OR and SF conceptualizing and advising the interim analysis. JML and PG have provided expert topical guidance for the evaluation of *Hope Groups*.


### Funding {4}

This research study and program are supported by the Global Parenting Initiative (GPI), the Moderna Foundation, and private donations to World Without Orphans. The GPI is funded by The LEGO Foundation, Oak Foundation, the World Childhood Foundation (16191), The Human Safety Net, ELMA Philanthropies, and the UK Research and Innovation Global Challenges Research Fund (ES/S008101/1). SF acknowledges the Engineering and Physical Sciences Research Council (EP/V002910/2).


### Availability of data and materials {29}

All de-identified data and study materials will be made freely available after the study, including the *Hope Groups* guide, ODK surveys, RCT consent forms, and recruiting information sheets.

## Declarations

### Ethics approval and consent to participate {24}

This study has been approved by the University of Oxford (Reference: R90037/RE001) and the Ukrainian Institute on Public Health Policy (Reference #00007612). Self-administered written informed consent is obtained from all participants and facilitators.

### Consent for publication {32}

Participants consented to the sharing of de-identified data and results. Model consent forms will be freely available with other study documents.

### Competing interests {28}

SH and NB participated in the development of the *Hope Groups* intervention, which is based on evidence from Parenting for Lifelong Health, developed by LC, JML, and LS. JML is also the Chief Executive Officer of Parenting for Lifelong Health. World Without Orphans supported the development of Hope Groups and supports the implementation of *Hope Groups* in this study. No profit or financial gain will be made from this intervention.


### Author details

[1]Department of Social Policy and Intervention, University of Oxford, Oxford, UK. [2]Department of Mathematics, Imperial College London, London, UK. [3]World Without Orphans/Ukraine Without Orphans, Kyiv, Ukraine. [4]Department of Computer Science, University of Oxford, Oxford, UK. [5]Children's Mission Ukraine, Kyiv, Ukraine. [6]Centre for Social Science Research, University of Cape Town, Cape Town, South Africa. [7]Parenting for Lifelong Health, Oxford, UK. [8]There Is Hope Krakow, Krakow, Poland. [9]Faculty of Health and Life Sciences, University of Exeter, Exeter, UK. [10]Maestral International, Minneapolis, USA. [11]Department of Psychiatry, University of Cape Town, Cape Town, South Africa. [12]Global Reference Group for Children Affected By Crisis, University of Oxford, Oxford, UK.


Received: 4 March 2024 Accepted: 10 June 2024
Published: 17 July 2024

### References


1. Social Cohesion and Resilience. World Bank https://www.worldbank.org/en/topic/social-cohesion-and-resilience.
2. Østby G, Rustad SA, Arasmith A. Children affected by Armed Conflict, 1990–2021, Conflict Trends, 2. Oslo: PRIO. - Google Search; 2022. https://www.google.com/search?q=%C3%98stby%2C+Gudrun%3B+Siri+Aas+Rustad+%26+Andrew+Arasmith+(2022)+Children+Affected+by+Armed+Conflict%2C+1990%E2%80%932021%2C+Conflict+Trends%2C+2.+Oslo%3A+PRIO.&oq=%C3%98stby%2C+Gudrun%3B+Siri+Aas+Rustad+%26+Andrew+Arasmith+(2022)+Children+Affected+by+Armed+Conflict%2C+1990%E2%80%932021%2C+Conflict+Trends%2C+2.+Oslo%3A+PRIO.&gs_lcrp=EgZjaHJvbWUqBggAEEUYOzIGCAAQRRg70gEHNDE1ajBqN6gCALACAA&sourceid=chrome&ie=UTF-8.
3. 110 million people displaced around the world: get the facts | International Rescue Committee (IRC). 2023. https://www.rescue.org/uk/article/110-million-people-displaced-around-world-get-facts.
4. The longitudinal impact of war exposure on psychopathology in Syrian and Iraqi refugee youth - Liza Marie-Emilie Hinchey, Raya Nashef, Celine Bazzi, Kathleen Gorski, Arash Javanbakht, 2023. https://journals.sagepub.com/doi/10.1177/00207640231177829?url_ver=Z39.88-2003&rfr_id=ori:rid:crossref.org&rfr_dat=cr_pub%20%200pubmed.
5. Barenbaum J, Ruchkin V, Schwab-Stone M. The psychosocial aspects of children exposed to war: practice and policy initiatives. J Child Psychol Psychiatry. 2004;45:41–62.
6. Hillis SD, Mercy JA, Saul JR. The enduring impact of violence against children. Psychol Health Med. 2017;22:393–405.
7. World Population Prospects - Population Division - United Nations. https://population.un.org/wpp/.
8. UNHCR: One year after the Russian invasion, insecurity clouds return intentions of displaced Ukrainians. UNHCR UK https://www.unhcr.org/uk/news/press-releases/unhcr-one-year-after-russian-invasion-insecurity-clouds-return-intentions.
9. Children in War and Conflict. *UNICEF USA* https://www.unicefusa.org/what-unicef-does/emergency-response/conflict.
10. Ukraine war: $100 billion in infrastructure damage, and counting | UN News. 2022. https://news.un.org/en/story/2022/03/1114022.
11. Almost 4.5 million Ukrainians returned home from displacement, need recovery support – IOM. https://ukraine.iom.int/news/almost-45-million-ukrainians-returned-home-displacement-need-recovery-support-iom.
12. Calam R, El-Khani A, Maalouf W. Editorial perspective: how can we help the children of Ukraine and others affected by military conflict? Child Adolesc Ment Health. 2022;27:294–6.
13. Betancourt TS, Khan KT. The mental health of children affected by armed conflict: protective processes and pathways to resilience. Int Rev Psychiatry Abingdon Engl. 2008;20:317–28.
14. Betancourt TS, McBain RK, Newnham EA, Brennan RT. The intergenerational impact of war: longitudinal relationships between caregiver and child mental health in postconflict Sierra Leone. J Child Psychol Psychiatry. 2015;56:1101–7.
15. Miller KE, et al. Supporting Syrian families displaced by armed conflict: a pilot randomized controlled trial of the Caregiver Support Intervention. Child Abuse Negl. 2020;106:104512.
16. Masarik AS, Conger RD. Stress and child development: a review of the Family Stress Model. Curr Opin Psychol. 2017;13:85–90.
17. Bogic M, Njoku A, Priebe S. Long-term mental health of war-refugees: a systematic literature review. BMC Int Health Hum Rights. 2015;15:29.
18. Jeong J, Franchett EE, Ramos de Oliveira CV, Rehmani K, Yousafzai AK. Parenting interventions to promote early child development in the first three years of life: a global systematic review and meta-analysis. PLoS Med. 2021;18:e1003602.
19. Gonçalves Júnior J, et al. The impact of 'the war that drags on' in Ukraine for the health of children and adolescents: old problems in a new conflict? Child Abuse Negl. 2022;128:105602.
20. Minkler M, Blackwell AG, Thompson M, Tamir H. Community-based participatory research: implications for public health funding. Am J Public Health. 2003;93:1210–3.

21. Hillis, S. et al. The effectiveness of Hope Groups, a mental health, parenting support, and violence prevention program for families affected by the war in Ukraine: findings from a pre-post study. 2024. SSRN Scholarly Paper at https://doi.org/10.2139/ssrn.4677018.
22. Center PR. Religious belief and national belonging in Central and Eastern Europe. In: Pew Research Center's Religion & Public Life Project. 2017. https://www.pewresearch.org/religion/2017/05/10/religious-belief-and-national-belonging-in-central-and-eastern-europe/.
23. Campbell MK, Piaggio G, Elbourne DR, Altman DG; for the CONSORT Group. Consort 2010 statement: extension to cluster randomised trials. BMJ. 2012;345:e5661.
24. Placebo effects during the waiting period for psychotherapy in patients with depression | Current Psychology. https://link.springer.com/article/10.1007/s12144-022-04206-4.
25. United Nations High Commission for Refugees (UNHCR). Conducting Rigorous Research in Humanitarian Contexts (n.d.). Accessed at: Policy-brief-Rigorous-Research-in-Humanitarian-Contexts-Final-1.pdf.
26. Kroenke K, Spitzer RL, Williams JBW, Löwe B. An ultra-brief screening scale for anxiety and depression: the PHQ-4. Psychosomatics. 2009;50:613–21.
27. Meinck F, et al. Adaptation and psychometric properties of the ISPCAN Child Abuse Screening Tool for use in trials (ICAST-Trial) among South African adolescents and their primary caregivers. Child Abuse Negl. 2018;82:45–58.
28. McEachern AD, et al. Parenting Young Children (PARYC): validation of a self-report parenting Measure. J Child Fam Stud. 2012;21:498–511.
29. Essau CA, Sasagawa S, Frick PJ. Psychometric properties of the Alabama Parenting Questionnaire. J Child Fam Stud. 2006;15:595–614.
30. Radloff LS. The CES-D Scale: a self-report depression scale for research in the general population. Appl Psychol Meas. 1977;1:385–401.
31. Yumpu.com. Parent-Child Communication, Child Report ... - Fast Track Project. yumpu.com https://www.yumpu.com/en/document/view/36797084/parent-child-communication-child-report-fast-track-project.
32. Cianchetti C, et al. Child and Adolescent Behavior Inventory (CABI): a new instrument for epidemiological studies and pre-clinical evaluation. Clin Pract Epidemiol Ment Health CP EMH. 2013;9:51–61.
33. Works, W. What Works - a rigorous global evidence review of interventions to prevent violence against women and girls, What Works to prevent violence against women and girls global Programme, Pretoria, South Africa. https://www.whatworks.co.za/resources/item/693-a-rigorous-global-evidence-review-of-interventions-to-prevent-violence-against-women-and-girls.
34. Parmar MK, Spiegelhalter DJ, Freedman LS. The CHART trials: Bayesian design and monitoring in practice. CHART Steering Committee. Stat Med. 1994;13:1297–312.
35. Bendtsen M. Avoiding under- and overrecruitment in behavioral intervention trials using Bayesian sequential designs: Tutorial. J Med Internet Res. 2022;24:e40730.
36. Prior distributions. In: Bayesian approaches to clinical trials and health-care evaluation. John Wiley & Sons, Ltd; 2003. p. 139–80. https://doi.org/10.1002/0470092602.ch5.
37. Thomas LE, Yang S, Wojdyla D, Schaubel DE. Matching with time-dependent treatments: a review and look forward. Stat Med. 2020;39:2350–70.
38. Witman A, et al. Comparison group selection in the presence of rolling entry for health services research: rolling entry matching. Health Serv Res. 2019;54:492–501.
39. An overview of the bayesian approach. In: Bayesian approaches to clinical trials and health-care evaluation. John Wiley & Sons, Ltd; 2003. p. 49–120. https://doi.org/10.1002/0470092602.ch3.
40. Randomised controlled trials. In: Bayesian approaches to clinical trials and health-care evaluation. John Wiley & Sons, Ltd; 2003. p. 181–249. https://doi.org/10.1002/0470092602.ch6.
41. Ukrainian prioritized multisectoral mental health and psychosocial support actions during and after the war: operational roadmap [EN/UK] - Ukraine | ReliefWeb. 2022. https://reliefweb.int/report/ukraine/ukrainian-prioritized-multisectoral-mental-health-and-psychosocial-support-actions-during-and-after-war-operational-roadmap-enuk.


## Publisher's Note

Springer Nature remains neutral with regard to jurisdictional claims in published maps and institutional affiliations.
