# Supplementary material for: Effectiveness of the hope groups intervention on mental health, parenting practices, and violence against children among Ukrainian parents and caregivers affected by war and displacement: a cluster randomised controlled trial
Source: Lancet Reg Health Eur. 2026 Aug 25;68:101783. doi: 10.1016/j.lanepe.2026.101783 (PMC13559926; doi:10.1016/j.lanepe.2026.101783)
Supplement: Supplementary file 2 [file mmc4.pdf]

Safeguarding and Monitoring

1. **Trial Monitoring Team**
2. **Child Safeguarding Policy**
3. **Protocol for Incident Reporting**
4. **Form for Incident Reporting**

Trial Monitoring Team

The trial monitoring team consists of coordinators, advisors, the programme director, and co-principal investigators. Roles are detailed as follows:

- The monitoring team includes four Ukrainian coordinators, who will contact all Hope Groups facilitators biweekly throughout the duration of the trial to assess participant well-being and monitor potential adverse events. Adverse events include traumatic responses to programme content, or disclosures during programme delivery requiring mandated reporting, such as suspected child maltreatment, exploitation, or any other violation to the policy. Coordinators will report adverse events to the study team within 24 hours of discovery. Coordinators will meet with the study team biweekly to discuss their meetings with facilitators and findings on participant well-being.
- The monitoring team includes three Ukrainian advisors, linked a non-governmental organisation (NGO) called Nehemiah, who will meet with the study team monthly to provide oversight, guidance, and input throughout the trial. Nehemiah is a Transcarpathian regional leader in child protection, family violence and mental health services, with policy and practice networks nationally, including partners such as UNFPA, UNHCR, Sema of Ukraine, Resilience Centres of Ukraine, the International Labor Organization (ILO), World Without Orphans (WWO), Ukraine Without Orphans (UWO), LAMb International, and many local partners. Their expert team of psychologists, case managers, and child protection professionals supported the development of the trial's safeguarding and reporting policies. Any adverse events will be reported to trial advisors, along with the University of Oxford and Ukrainian Institute on Public Health Policy, within 24 hours. Any modifications to the protocol will be reported to trial advisors, the University of Oxford, and Ukrainian Institute on Public Health Policy. During monthly meetings, the study team will discuss with advisors the trial's progress, participant well-being, and any harms identified. Additionally, interim analysis results will be reviewed in meetings with Ukrainian advisors, including immediately disclosing any harmful effects revealed in the data (e.g., if interim analyses reveal worsening participant outcomes in the intervention arm compared to the control arm), and reviewing the decision to deliver the intervention to wait-list control participants based on interim evidence.
- The monitoring team includes the Programme Director (NB) and Co-Principal Investigators (SH, LC, ST). The Programme Director was

responsible for training coordinators and facilitators on the safeguarding policy and reporting protocol. The Programme Director and Co-Principle Investigators are responsible for reviewing all adverse events from coordinators or facilitators within 24 hours and determining necessary action.

Child Safe-Guarding Policy - Hope Groups

Latest Version Approved: 24th February 2022

INTRODUCTION 2

PREMISE – OUR DIRECTIVES 2

CORE PRINCIPLES 3

RIGHTS OF EVERY CHILD 3

SERVING OUR PARTNERS 5

PREVENTION OF ABUSE 6

CHILD SAFEGUARDING POLICIES 6

ABUSE CONCERNS DEFINED 6

HOPE GROUPS TEAM 10

GUIDELINES FOR SAFEGUARDING 10

CODE OF CONDUCT 11

Acceptable Behavior 11

Unacceptable Behavior 12

REPORTING and RESPONDING 13

ADDRESSING AND REPORTING ABUSE CASES 14

RESPONDING TO REPORTS OF ALLEGED ABUSE 14

ACKNOWLEDGEMENTS 15

APPENDIX 1: STATEMENT OF COMMITMENT – HOPE GROUPS TEAM 16

APPENDIX 2: STATEMENT OF COMMITMENT – HOPE GROUPS PARTNERS 17

INTRODUCTION

PREMISE – OUR DIRECTIVES

Hope Groups, under the direction of World Without Orphans Global, is committed to safeguarding children from abuse and exploitation and believes all children have the right to be protected from harm. The welfare and best interests of the child are paramount for Hope Groups when making decisions regarding policy.

WWO Global/Hope Groups serves a collective group of countries, organizations, NGOs, churches, faith and community leaders, and civil society members who are working to serve orphans and vulnerable children as well as to strengthen families. Therefore, WWO Global accepts responsibility to issue a clear statement of our role and commitment to the safeguarding of all children. All Hope Groups staff and volunteers are required to read and sign the WWO Global Child Safeguarding Policy.

Although WWO Global does not directly work with families or children, we do promote and support those who do. Thus, we expect partner organizations with which WWO Global has partnership agreements to have and enforce a Child Safeguarding Policy. We further strongly encourage any organization or network with which WWO Global partners to have and enforce a Child Safeguarding Policy. This includes partner organizations implementing Hope Groups in their services.

WWO Global believes that all children have the right to be protected from harm without discrimination of any kind, irrespective of the child's parents or legal guardian, race, gender, skin color, sex, language, religion, political or other opinion, nationality, ethnic or social origin, poverty, disability, birth order, or beliefs.

We acknowledge that children can be victims of physical, emotional, social, sexual abuse, neglect, or exploitation. This safeguarding policy is to clarify our role and further identify the expectations of the partners with whom we serve.

We advocate for children to be cared for in the best possible way. We are in agreement with the United Nations Convention on the Rights of the Child (UNCRC), which is committed to improving the lives of children worldwide. The convention has identified 4 Core Principles and 12 Rights for Every Child, which are embodied in the commitment from WWO Global addressed in this document.

CORE PRINCIPLES [1]

Principle 1. Non-Discrimination

One general principle as identified by the committee on the rights of the child is that all children should enjoy their rights and should never be subjected to any discrimination.

Principle 2. Best interests of the child

Article 3:1. "In all actions concerning children whether undertaken by public or private social welfare institution, courts of law, administrative authorities or legislative bodies, the best interest of the child shall be a primary consideration."

Principle 3. The right to survival and development

The principle most directly related to children's economic and social rights is formulated in the right to life article. Article 6:2 and states thus: "State parties shall ensure to the maximum extent possible the survival and development of the child."

Principle 4. The views of the child

Article 12:1 "States parties shall assure to the child who is capable of forming his or her own views the rights to express those views freely in all matters affecting the child, the view of the child being given due weight in accordance with the age and maturity of the child.

RIGHTS OF EVERY CHILD [2]

1. Every child has the right to be born well.

It is the responsibility of the parents to make sure they can provide a safe environment for their unborn child. This includes proper medical attention and care from conception, birth, and throughout childhood years in a newborn services unit or pediatric center.

2. Every child has the right to a wholesome family life.

The child's first learning environment and teachers are their home and family. They are entitled to be a part of a loving family that will instill ethical values and morals in them.

3. Every child has the right to be raised well and become a contributing

[1] United Nations Convention on the Rights of the Child (UNCRC)

[2] Ibid.

member of society. By raising them in a safe and loving environment, parents and guardians can shape the personalities of their young to be useful and contributing members of their respective communities when they grow older.

4. **Every child has the right to basic needs.**

The four basic needs of people outlined in the law are as follows: a balanced diet, adequate clothing, sufficient shelter, and proper healthcare. This also includes any other requirements to lead a healthy and active life.

5. **Every child has the right to access what they need to have a good life.**

This right goes beyond the basic needs and focuses more on the atmosphere of the place they will be raised in. A child's needs must always be attended to so they feel the support of people around them, which in turn will build and strengthen their character in adulthood.

6. **Every child has the right to education.**

In an ideal world, every child should have the means to go to a classroom and have access to books and learning materials that can enrich their intelligence and skills.

7. **Every child has the right to play and enjoy their youth.**

Children have the right to engage in wholesome recreational activities whenever they wish and not be exploited for events that are deemed only for adults to do, i.e., intensive manual labor.

8. **Every child has the right to be protected from danger.**

This includes all hazards that could affect their physical, mental, and emotional states, such as removing them from dangerous living situations, preventing them from getting into accidents, or protecting them from the abuse of adults, to name a few.

9. **Every child has the right to live in a productive environment.**

Safe communities that inspire them to give back when they are older should surround children. This means staying away from bad influences and situations that can cause harm to their health.

10. **Every child has the right to be cared for in the absence of their parent or guardian.**

If the parent or guardian fails to fulfill their role, the State shall assume custody and care for the child, providing them with their fundamental needs for growth and development.

11. **Every child has the right to good governance.**

Children also have a right to be born under the presence of good governance that can inspire them to become helpful and active citizens. This doesn't necessarily mean they have to get involved with politics but rather have an

interest in being involved in political discussions for the betterment of their country.

12. Every child has the right to freedom and peace.
Last but not least, every child is entitled to do whatever they want in their lives, so long as it contributes to the peace and betterment of the communities they are a part of.

SERVING OUR PARTNERS

OUR COMMITMENT

WWO Global/Hope Groups reserves the right to communicate to partners best practices related to the rights of the child to ensure their safety and protection from harm. We view this responsibility as a priority to help others know and understand how their contribution can help improve or hurt the well- being of children and specifically to those who are orphans and/or vulnerable.

OUR STANDARDS

Hope Groups work under the following standards, which are benchmarks of commitment to assess and communicate quality intervention strategies and practices to our global partners.

- Promote and teach practices and tools that work toward protecting children from abuse and maltreatment.
- Communicate clear and evidence-based learning to serve in preventing harm to children's health or development.
- Promote the best in research that leads to ensuring children grow up with the provision of safe and effective care.
- Respond to partners where it becomes necessary in taking action to enable all children and young people to have the best outcomes.
- Communicate to partners the importance of endorsing and following the principles noted in the UNCRC.
- Maintain close contact with partners to support and serve in helping develop and implement a Child Safeguarding Policy for their organization, church, etc.
- Make available (when requested) to all partners samples of Child Safeguarding Policies and offer coaching to develop such policies for partners.
- Promote awareness that those reporting an abuse are to be treated with respect and dignity.

PREVENTION OF ABUSE

CHILD SAFEGUARDING POLICIES

The leadership of Hope Groups commits to being responsive to ensuring all partners are aware of the following:

- Hope Groups facilitators and coordinators will keep Hope Groups leadership informed of Child Safeguarding issues, incidents and concerns and have strategies to develop a unified response when a concern arises.
- Hope Groups facilitators and coordinators will maintain confidentiality, when possible, within the standards and guidelines of reporting responsibilities - to ensure there are open doors for communication to review and address internal concerns.
- Partner organizations of Hope Groups will have an updated Child Safeguarding Policy on hand, communicate this to their teams and make this policy available to WWO Global/Hope Groups if requested.
- Partners will communicate to their staff, volunteers, and children a clear, safe, and accessible way for each and every child to express concerns about their own care and safeguarding.
- Partners will provide clear and specific details of the steps their leadership might need to take should a concern arise and need outside intervention.
- Hope Groups leadership will provide clear and specific direction when needed to address a child safeguarding concern where partner might not be responding.
- Hope Groups leadership will clearly identify steps Hope Groups leadership will need to take should reporting become an issue of concern.

ABUSE CONCERNS DEFINED

Child Abuse

Child abuse and neglect are defined as all forms of action or inaction resulting in harm or risk of harm to children. Six key types of abuse include:

1. **Physical Abuse** – results in actual or potential physical harm from an interaction or lack of interaction, which is reasonably in the control of a parent or person in a position of responsibility, power, or trust. Examples include hitting, poisoning, burning, slapping, violent shaking,

drowning, suffocating, or deliberately making a child ill.

2. **Sexual Abuse** – is when a child is used by an adult or young person (in a physical, visual, verbal, psychological, online, or other way) for that person's sexual gratification. Any sexual involvement with a child, with or without their consent, is still considered abuse.
3. **Emotional Abuse** – is the persistent ill treatment of a child, such as causing a severe and persistent adverse effect on the child's emotional development; or failure in providing a developmentally supportive environment. This abuse may include repeatedly rejecting children, humiliating them, or denying their worth andrights as human beings
4. **Exploitation** – refers to the use of the child in work or other activities for the benefit of others. This includes, but is not limited to, child labor and child prostitution. These activities are to the detriment of the child's physical or mental health, education, moral or social-emotional development.
5. **Neglect** – or negligent treatment is the persistent failure to meet a child's basic needs when options are available, including the failure to provide adequate health care, supervision, clothing, nutrition, housing, as well as their physical, emotional, social, educational, and safety needs. Neglect is likely to result in the serious impairment of the child's health or development.
6. **Spiritual Abuse** – occurs when someone in a position of spiritual power or authority in a faith-based environment misuses their power and the trust placed in them, with the intention of controlling, coercing, manipulating, or dominating a child.

Spectrum of Sexually Abusive Behaviours[3]

There are two main types of child sexual abuse: touching and non-touching. Touching includes touching a child's genitals, making a child touch someone else's genitals, playing sexual games, and/or putting objects or body parts inside the vulva or vagina, in the mouth, or in the anus of a child for sexual pleasure. Non-touching abuse includes showing pornography to a child, exposing a person's genitals to a child, prostituting/trafficking a child, photographing a child in sexual poses, encouraging a child to watch or hear sexual acts either in person or on a video, and/or watching a child undress or use the bathroom.

Sexual activity between an adult and a child may range on a continuum from exhibitionism to intercourse, often progressing through the following spectrum of behavior:

- **Nudity.** The adult parades nude around the house in front of all or some of

[3] Reference: *Family-Centered Child Protective Services (Core 101), © Institute for Human Services, May 2001*

the family members.

- **Disrobing.** The adult disrobes in front of the child, generally when the child and the adult are alone.

- **Genital Exposure.** The adult exposes his or her genitals to the child. Here the perpetrator directs the child's attention to the genitals. This may involve a request that the child touch the parent's genitals.

- **Observation of the child.** The adult surreptitiously or overtly watches the child undress, bathe, excrete, or urinate.

- **Kissing.** The adult kisses the child in a lingering and intimate way ("French kissing.") Even very young children sense the inappropriateness of this behavior and may experience discomfort.

- **Fondling.** The adult fondles the child's breasts, abdomen, genital area, inner thighs, or buttocks. The child may be requested to similarly fondle the adult at his or her request.

- **Masturbation.** The adult masturbates while the child observes; the adult observes the child masturbating; the adult and child observe each other while masturbating themselves; or the adult and child masturbate each other (mutual masturbation).

- **Fellatio.** The adult has the child fellate him, or the adult will fellate the child. This type of oral-genital contact requires the child to take a male perpetrator's penis into his/her mouth, or the adult to take the male child's penis into his/her mouth.

- **Cunnilingus.** This type of oral-genital contact requires the child to place mouth and tongue on the vulva or in the vaginal area of the adult female, or the adult will place his or her mouth on the vulva or the vaginal area of the female child.

- **"Dry Intercourse."** This slang term describes an adult who rubs his penis against the child's genital-rectal area, inner thighs, or buttocks.

- **Digital (finger) penetration of the anus or rectal opening.** This involves penetration of the anus or rectal opening by a finger, or inanimate objects such as crayons or pencils.

- **Penile penetration of the vagina.** This involves penetration of the vagina by a male perpetrator's penis (intercourse).

- **Penile penetration of the anus.** This involves penetration of the anus by a male perpetrator's penis (intercourse).

- **Sexual Exploitation.** The typical definition of child sexual exploitation is the act of coercing, luring, or engaging a child under the age of 18 into a sexual act and involvement in the sex trade or pornography, with or without the child's consent, in exchange for money, drugs, shelter, food, protection or other necessities.

- **Online sexual abuse and child pornography** - Online sexual abuse is a relatively modern trend, in which perpetrators utilize modern forms of technology such as live stream web cameras, cell phones, or social media to coerce targeted victims into inappropriate and sometimes illegal sex acts. Children are often the targets of online sexual predators who will often bully, emotionally manipulate, blackmail, or befriend willing communicators on the web in order to obtain their desires. Online sexual abuse may vary from personal interactions between a victim and offender to a process in which children are sexually exploited for the perpetrators' profit. Online abuse may be linked to child pornography, defined as any visual depiction of sexual images or sexually explicit conduct involving a minor (persons less than 18 years old). Images of child pornography are referred to as child sexual abuse images. Any production, distribution, importation, reception, or possession of any image of child pornography is prohibited and should be reported immediately.

Other Specific Considerations that Constitute Child Abuse [4]

- **Traditional Harmful Practices** – are cultural practices which may put the child at risk of harm. Examples are Child Marriage, where the child is under the legal age of consent and Female Genital Mutilation.
- **Bullying** – takes the form of physical intimidation; verbal intimidation, which includes racist and sexist remarks; or emotional intimidation such as isolating or excluding a child whereby they experience deliberate hostility.
- **Witchcraft/Ritualistic Abuse** – occurs within some communities that believe strongly that children may be possessed by evil spirits. Children may be abused through ritualistic practices and ceremonies, in the form of beating, maiming, rape, and even as sacrifices.
- **Historical Abuse** – is any type of abuse that may have occurred several years before it is disclosed.

[4] Adapted from Child Safeguarding Policy. Christian Broadcasting Network (CBN), 2021

HOPE GROUPS TEAM

GUIDELINES FOR SAFEGUARDING[5]

Hope Groups Program Managers, Local Coordinators, and Group Facilitators, whether paid or volunteers, must comply with the terms of this policy and are required to sign the Statement of Commitment (See Appendix 1).

It is the responsibility of Hope Groups to ensure all their global team personnel, including paid or unpaid staff, volunteers, freelancers, consultants, and visitors are aware of this child protection policy and agree to abide by and uphold the principles. They will be required to sign the Statement of Commitment (Appendix 1) indicating they have received the policy, read it, understood it and that they agree to abide by it.

If found by the organization or project to be a risk to children, or if that risk is confirmed from another source, the individual will not be allowed to work again in the Hope Groups project.

Hope Groups seeks to have representatives and partners who respect and value children and are committed to safeguarding them from harm; therefore, Hope Groups staff, volunteers, and partners are required to abide by the Hope Groups Child Safeguarding Policy.

Partner Organizations implementing their own Hope Groups program and/or representing Hope Groups should have their own Child Safeguarding Policy, which is being actively used and implemented by all of their own paid or unpaid staff, volunteers, freelancers, consultants, and visitors. It must meet the standards of the WWO Global/Hope Groups Child Safeguarding Policy and provide specific policies and procedures for how to prevent abuse of children in their care as well as how they will respond to any abuse cases and report if appropriate.

If Partner Organizations do not have their own child protection policy, or if it is inadequate, WWO Global's/Hope Groups will make this policy available to them as a guide to assist them in putting a suitable policy and procedures in place to safeguard children. Hope Groups leadership will also be available to answer any questions they may have about WWO Global's/Hope Groups Child Safeguarding Policy throughout the process of developing their own child safeguarding policy and procedures. All Partner Organizations implementing their own Hope Groups program must complete and sign the Statement of Commitment (Appendix 2).

---

[5] Ibid.

CODE OF CONDUCT[6]

The Code of Conduct for Hope Groups describes acceptable standards of behavior and promotes good practices so children can experience a safe, positive, and encouraging environment. It sets a clear benchmark for acceptable conduct expected from all Hope Groups coordinators and facilitators, whether paid or volunteer, to minimize opportunities for the abuse of children, but also help to prevent unfounded allegations. Hope Groups partner organizations should ensure that all their paid and unpaid staff, volunteers, freelancers, consultants, and visitors are held to these standards.

Acceptable Behavior

- Treat all children fairly and with respect and dignity, regardless of race, religion, social background, gender, skin color, disabilities, ethnicity, nationality, or beliefs.

- Uphold the integrity of Hope Groups by ensuring that personal and professional conduct is, and is seen to be, of the highest standard.

- Prevent, oppose and combat all exploitation and abuse of children.

- Refrain from any involvement in criminal or unethical activities, activities that contravene human rights, or activities that compromise the image and interests of Hope Groups.

- Comply with all international and local legal requirements with regard to children, including the protection of personally identifiable information (e.g., images, names, addresses, criminal background, medical records, biometrics, and religious affiliation).

- Ensure that the risks of being alone with children are minimized by having at least two adults supervising, and always being visible to others when with children.

- Be committed to creating a culture of openness and mutual accountability where child protection issues can be openly discussed and abusive behavior must be challenged.

[6] Ibid.

- Be aware of the power balance between an adult and child and avoid taking any advantage this may provide.

- Be aware that for visitors, their presence with children will often be temporary and they should avoid creating bonds with children which encourage emotional or psychological dependence.

- Be aware that they may work with children who, because of the circumstances and abuses they have experienced, may use a relationship to obtain "special attention." The adult is always considered responsible even if a child demonstrates sexually stylized behavior.

- Listen to children and encourage them to raise their concerns about staff or others safely.

- Take steps to help train and inform children of what is acceptable and unacceptable behavior.

- Use positive, non-violent methods to manage children's behavior.
- Ensure physical contact is appropriate at all times and not an invasion of the child's privacy.

- Raise any concerns of inappropriate behavior immediately.

Unacceptable Behavior

Hope Groups team members must **not**:

- Develop physical or sexual relationships with children.

- Spend time alone with a child away from others, behind closed doors, or in a secluded area.

- Behave physically in a manner which is inappropriate or sexually provocative. Think of the bathing suit rule. An adult should never touch a child where the child's bathing suit would be covering him/her. Adults should take careful consideration to not hug children in a close (body to body) lingering manner.

- Take a child to their home, including staying overnight at their home unsupervised, or visit a child at their home where they may

be alone with that child.

- Sleep in the same room or bed as a child with whom they are working.
- Engage in sexual activity with a child regardless of the national legally accepted age of consent. (Mistaken belief regarding the age of the child is not a defense).
- Be intoxicated or under the influence of alcohol or drugs prior to or while assuming responsibility for any child.
- Hit or otherwise physically assault or physically abuse children, even where this may be culturally acceptable.
- Act in ways that may be abusive or may place a child at risk of abuse.
- Use language that will mentally or emotionally harm a child.
- Condone, or participate in behavior with children which is illegal, unsafe, or abusive.
- Exploit children for their labor (e.g. domestic servants) or for sexual purposes.
- Take a child alone in a vehicle unless necessary and with parental/guardian consent.
- Conduct or be a part of harmful traditional practices, spiritual or ritualistic abuse.
- Act in any way that intends to embarrass, shame, humiliate or degrade a child.
- Stay silent, cover up, or enable any known or suspected safeguarding incident or breach of the Child Safeguarding Policy by personnel.

REPORTING and RESPONDING

WWO Global/Hope Groups will address any suspected incidents of suspected child abuse or exploitation according to the guidelines below. At all times, the best interests of the child are of paramount consideration.

ADDRESSING AND REPORTING ABUSE CASES

In all cases, if a child discloses an alleged abuse case, or an adult has suspicion of abuse, the adult must:

- Listen to the child, react calmly, take them seriously, and reassure them they have done the right thing by disclosing.
- Avoid promising the child total confidentiality as it is in their best interests that key trusted people also know who can help.
- Ensure the safety of the child.
- Avoid gossip and making assumptions.
- Avoid informing or confronting the alleged perpetrator(s).
- Record in detail precisely what the allegations are. Avoid leading questions. Focus on what was said, observed, who was present and what happened. Avoid speculation and interpretation.
- Report the alleged abuse to appropriate legal, medical, and/or child protection authorities

RESPONDING TO REPORTS OF ALLEGED ABUSE

Allegations of abuse within the Hope Groups program will be reviewed within 24 hours of being noted. The Hope Groups Facilitator will report in writing the suspected child abuse to their local Hope Groups Coordinator, who will send that report to the overall Program Manager (*a form is provided on which to document this report)*. In addition, any Hope Groups Facilitator or Coordinator who suspects child abuse will report that allegation to an independent party in the country of origin (i.e. police, child protective hotline, or other recognized local authority on child abuse) to investigate and determine what the next steps should be. In every case, the independent national party will be asked in writing by the Hope Groups Facilitator and/or Coordinator to report allegations of child sexual abuse to the appropriate national legal authorities.

Hope Groups expects all Partner Organizations to follow the guidelines of their Child Safeguarding Policy for their organization to ensure the safety and well-being of the child, and are solely responsible to handle any report of alleged abuse of a child in accordance with their own Child Safeguarding Policy, and local laws of its jurisdiction.

Partner Organizations of Hope Groups are responsible to conduct investigations of suspected abuse and report to Hope Groups final results

and recommendations coming from the investigation. Hope Groups will then act accordingly to the policy herein and the results of the independent investigation of the partner.

Hope Groups shall have no obligation or responsibility to assist any Partner Organization in its investigation of any such alleged abuse of a child, nor shall they have any obligation or responsibility to a Partner Organization for any costs, expenses, damages, fines, penalties, or liabilities that a Partner Organization may incur in connection with any such alleged abuse of a child.

ACKNOWLEDGEMENTS

WWO Global/Hope Groups is most appreciative of the generous permission from Christian Broadcasting Network (CBN) for allowing WWO Global to adapt and model this Child Safeguarding Policy off their extensive work to create the same for their organization. We are grateful for all the additional references we found and were able to adapt from. The material herein represents some of the best we have found across the globe.

We encourage each of you to use what you find useful here as well.

APPENDIX 1: STATEMENT OF COMMITMENT – HOPE GROUPS TEAM

STATEMENT OF COMMITMENT

to WWO Global's/Hope Groups Child Safeguarding Policy

I, ____ (name), have received, read, and understood the WWO Global Child/Hope Groups Safeguarding Policy. I agree to abide by the policy and principles therein and commit to protecting all children in our care from physical, sexual and emotional abuse and affirming their worth as being created in the image of God.

(Print your full name) (Date)

______________________________ _______________

(Signature)

____

Note: This Statement of Commitment is to be signed by all staff (paid or unpaid), volunteers, and contractors, who are in any way involved with projects or programs involving children and who are considered part of the Hope Groups Team.

APPENDIX 2: STATEMENT OF COMMITMENT – HOPE GROUPS PARTNERS

STATEMENT OF COMMITMENT

as a Hope Groups Partner Organization

I,____(name), representing

____________________________________(organization) have received, read, and understood the WWO Global/Hope Groups Child Safeguarding Policy. I hereby:

____Confirm that ________________________________(organization) has a Child Safeguarding Policy in place which is actively used and implemented by all our own paid or paid staff, volunteers, freelancers, consultants, and visitors, and is committed to protecting all children in our care from physical, sexual and emotional abuse and affirming their worth as being created in the image of God. A copy of this policy is attached along with my signed Statement of Commitment.

____Confirm that ________________________________(organization) does not have a Child Safeguarding Policy in place, or that the policy in place is inadequate, and will use the WWO Global/Hope Groups Child Safeguarding Policy as a guide to put a suitable policy and procedures in place to safeguard the children in our care and protect them from physical, sexual and emotional abuse and affirm their worth as being created in the image of God. A copy of this policy will be provided immediately upon completion.

(Print your full name) (Date)

______________________________ ________________

(Signature)

____

Note: This Statement of Commitment is to be signed by all Partner Organizations implementing their own Hope Groups Program.

Please send the completed original or a scanned copy to Hope Groups Leadership Team, at hopegroups@worldwithoutorphans.org.

Protocol of Action for Incident Reporting: Training for Facilitators and Coordinators When Cases of Violence are Detected

This protocol is guided by:
Law of Ukraine No. 2229-VIII (07.12.2017) "On Prevention and Combating Domestic Violence"
The Law of Ukraine "On the Protection of Childhood"
PROCEDURE for consideration of appeals and reports about child abuse or the threat of it

1. Reporting of violence against a child

The Hope Groups Child Safeguarding Policy clearly defines forms of child abuse. All Hope Groups personnel are required to read and sign the Safeguarding Policy.

1.1 If you become aware of child abuse, violence or attempted violence against a child, immediately (within one day) notify the internal affairs authorities (police), and children's affairs service.

1.2 If you witness child abuse, child abuse, or attempted child abuse:

- make sure you are safe;
- try to stop the act of violence against the child as soon as possible;
- call the internal affairs authorities (police);
- call the children's services;
- call for medical help (even if the child has no external signs of damage);
- stay with the child until the arrival of the police, children's services, medical assistance;
- be sure to report to the police.

1.3 In addition to the police and the children's service, complaints about child abuse or the threat of child abuse may be submitted to any entity specified in Clause 2 of Section I of the Procedure for Handling Appeals and Reports of Child Abuse or Threats of Child Abuse." Subjects are children's affairs service, child welfare institutions, centers of social services for children, families and youth, internal affairs bodies, educational institutions, health care institutions.

1.4 Children are victims of domestic violence even when they only witness it. In such cases, the police should be called and children's services should be contacted.

1.5 Submit the reporting form to your Hope Groups coordinator within 24 hours. The coordinator must send the completed form to the Program Director.

2. Reporting violence against an adult incapacitated person.

2.1. If you become aware of abuse/violence or attempted violence against an adult incapacitated person, you should report within 24 hours:

- inform the internal affairs authorities (police); the consent of the victim is not required (in accordance with clause 7 of article 4 of the Law of Ukraine "On Prevention and Counteraction of Domestic Violence")

2.2. If you are a witness of abuse / violence against an adult incapacitated person or an attempt to commit violence:

- make sure you are safe;
- try to stop the act of violence as soon as possible;
- call the internal affairs authorities (police);
- call for medical help (even if the victim has no external signs of damage);
- stay with the injured person until the arrival of the police and medical assistance;
- be sure to report to the police.

2.3. In addition to the police, complaints about ill-treatment or a case of violence against an adult incapacitated person can be submitted to guardianship authorities. The guardianship authorities are based on local districts, [districts in the cities of Kyiv and Sevastopol state administrations, executive bodies of city, district in cities, village, village councils (Article 56 of the Civil Code of Ukraine)].

2.4. Submit the reporting form to your Hope Groups coordinator within 24 hours. The coordinator must send the completed form to the Program Director.

3. Reporting violence against an adult with legal capacity

3.1 If you become aware of domestic violence (you were informed about the act(s) of violence by the victim or third parties), if possible:

- provide the injured person with information about the need to contact the police;
- provide the victim with information about the existence of free assistance services for victims of violence (psychological, social, legal);
- provide the affected person with information about the existence of shelters for affected persons;
- provide the affected person with the necessary contacts (phone numbers of hotlines, state and non-state assistance services).

3.2 If you are a direct witness of violence:
If you witness violence (you hear screams, sounds of beatings, other sounds that may indicate violence is being committed or you see violence being committed):

- make sure you are safe;
- call the authorized authorities - the police (when calling, state that this is a case of domestic violence);
- call an ambulance (when calling, state that this is a case of domestic violence);
- if possible, stay with the injured person until the police arrive.

Message mechanism:

All messages are to be made by telephone to the relevant services. These services must record calls in special journals provided for by their internal procedures. All referrals between subjects are carried out according to the forms determined by their internal procedure of conducting affairs by telephone and e-mail.

Hope Groups provides a reporting form for documenting any cases of violence that a facilitator or coordinator reports to the authorities. This form is NOT a substitute for notifying the police, children's services, and/or a hotline for preventing and combating violence. The purpose of this form is only for internal records. Any Hope Group personnel who become aware of violence or abuse should first notify the appropriate authorities and then complete the Hope Groups report and submit it to their coordinator. All completed forms are then submitted to the Project Assistant, Olga Ruina. She will notify the Principal Program Coordinator, Nicole Baldonado.

Contacts

Ukraine:

To call the police in Ukraine, the number is the same for the entire country, free of charge from all phones - 102.

Children's service numbers vary depending on the locality.

National hotline for preventing and combating violence, free of charge from all phones - 15-47

Poland:

Police: 112 (24 hours, emergency)

Report a suspicion of violence against a minor by calling the free 24-hour number of the Polish National Referral Service for Victims of Domestic Violence „Niebieska Linia" phone 800 120 002.

Other helpful resources:

Are You Experiencing Violence? https://bezprzemocy.krakow.pl/EN/are-you-experiencing-violence/

Blue Line: https://www.niebieskalinia.pl/en

Cyprus:

Police: 199 / 112

Police Safeguarding Sub-Directorate: 22808442; domviol.childabuse@police.gov.cy

Helpline 1440 is staffed by psychologists and social workers, trained in using communication techniques over the phone. The 24-hour helpline is free of charge and can help to provide:

– Counseling support for domestic and/or intimate partner violence issues, and/or gender-based violence issues, concerning you or other people.
– Information on other services related to domestic violence and/or intimate partner violence, and/or gender-based violence issues.
– Information about your rights and options in order to find the best possible solution for you.

- Website: https://domviolence.org.cy/en/1440-2/

Germany:

Police: 110 (usually only German speaking)

Child Protection Hotline: +49 30 61 00 66, operating 24/7 in multiple languages

Youth Welfare Office. Find the office in your federal state here: https://www.jugendaemter.com/jugendaemter-in-deutschland/

If you do not speak German, call the help centre of "Gewalt gegen Frauen" ("Violence against women") organisation instead. Their staff are available around the clock and speak many languages, including English, Arabic, French, Farsi/Dari, Kurdish (Kurmanchi) and Turkish. You can reach their team at 08000 116 016. They can offer anonymous and cost-free advice and can help locate a women's shelter in the area.

There are some drop-in centres responsible for investigating and certifying victims' injuries anonymously and free of charge. Ask the staff at the Opfervereins WEISSER RING to recommend you a contact point in your area. The staff of this association are available every day from 7:00 to 10:00 at 116 006. They mainly speak German and English and can offer you advice cost-free and anonymously.

Other helpful resources:

Berlin Emergency Child Protection Service: https://www.berlin.de/notdienst-kinderschutz/

Handbook Germany - Youth Welfare Office: https://handbookgermany.de/en/youth-welfare-office

Spain:

Police: 091

National Hotline for Domestic Violence: 016 (free of charge; 24 hours/day). You can speak to professionals with guaranteed confidentiality in 53 different languages. You can also email 016-online@igualdad.gob.es or WhatsApp 600 000 016.

Free Child Protection Helpline Infància Respon: 116 111 (Department of the Directorate General of Child and Adolescent Services)

Other helpful resources:

Emergency services: 112

Free legal advice and information: 061

Web of support and prevention resources in cases of gender violence (WRAP), Government Delegation against Gender Violence - https://wrap.igualdad.gob.es/recursos-vdg/search/SearchForm.action

France:

Police: 112 from a mobile phone, 17 from a landline

- It's also possible to make a report to the police online: https://www.service-public.fr/cmi

National Hotline for Violence against Women: 3919 (Anonymous and free, accessible from a landline and a mobile in metropolitan France and in the overseas departments. Hotline operators listen and provide information, and make appropriate referrals to local support and support systems. 3919 is not an emergency number.

National Helpline for Children in Danger: 119

Other helpful resources:

French government site on support for survivors of sexual violence: https://arretonslesviolences.gouv.fr/

Hope Groups Incident Reporting Form

Facilitators are to use this form on suspicion of child abuse; domestic violence; gender - based violence.

If you, as a Hope Groups facilitator, suspect child abuse, domestic violence , or gender - based violence, report it to the appropriate authority in your area within 24 hours. Contact information for local authorities are provided on the following page.

After you have notified the authorities, complete this incident report for Hope Groups' internal records. If you have any questions, contact your programme coordinator.

Your surname, first name, paternal name : ____
Your phone number : ____
Name of your Hope Group coordinator : ____
Phone number of your Hope Group coordinator : ____________________________________

Notification date (when you learned about the incident):	Date of the incident (in the words of the victim):	Did the person themself report the incident?<br><br>Yes No
Gender of the victim:	Age of the victim:	Disability:<br><br>Yes No
Family composition:	Residency status:<br>IDP<br>Local<br>Not specified	Place of residence:

Details of the incident<br>(Narrative of the incident/Description of the incident - concisely state the details of the incident in the words of the participant)<br><br>____<br>____<br>____<br>____<br>____

____
____
Describe how you learned about the incident - where, when, from whom
____<br>____<br>____<br>____
Information about the suspected perpetrator

Number of perpetrators:	Gender of the perpetrator:
Age of the perpetrator:	Occupation of the perpetrator:
Relationship between the perpetrator and the victim:	
○ Intimate partner / former partner<br>○ Main guardian<br>○ Relative other than husband/wife or guardian/custodian<br>○ Manager / employer<br>○ Teacher / school employee<br>○ Service provider	○ Cohabitant<br>○ Classmate<br>○ Family friend / neighbor<br>○ Another IDP / returnee<br>○ Another member of the community who lives on a permanent basis<br>○ Unknown

Information about your report		
Date of your report :	The authority to which it was reported :	To what phone number was it reported :

Result of the report :	If you reported suspected child abuse , did you notify the parent / guardian ?<br>Yes No

Your signature :

____

Please send this completed form to your Hope Groups Coordinator.
