# Supplementary material for: Effectiveness of the hope groups intervention on mental health, parenting practices, and violence against children among Ukrainian parents and caregivers affected by war and displacement: a cluster randomised controlled trial
Source: Lancet Reg Health Eur. 2026 Aug 25;68:101783. doi: 10.1016/j.lanepe.2026.101783 (PMC13559926; doi:10.1016/j.lanepe.2026.101783)
Supplement: Supplementary file 3 [file mmc5.pdf]

Hope Groups: A Cluster Randomized Controlled Trial of Psychosocial and Parenting Support Groups for Ukrainian Caregivers Affected by War

Statistical Analysis Plan

April 23, 2024

1. Administrative Information

Title of Trial	Hope Groups: A Cluster Randomized Controlled Trial of Psychosocial and Parenting Support Groups for Ukrainian Caregivers Affected by War
Trial registration	Open Science Framework, November 9, 2023. Registration DOI: https://doi.org/10.17605/OSF.IO/UVJ67
SAP Version	*This Statistical Analysis Plan (SAP) was written on April 5, 2024; Version 2.0.*

1.1 Roles and responsibilities

This SAP was produced by members of the trial team as described in the table:

Name	Affiliation(s)	Role
Sydney Tucker, MPH	University of Oxford, Imperial College London	Co-Investigator and Analyst
Susan Hillis, PhD	Global Reference Group for Children Affected by Crisis	Principal Investigator
Lucie Cluver, DPhil	University of Oxford	Principal Investigator
G.J. Melendez-Torres, DPhil	University of Exeter	Statistician and Trial Advisor
Oliver Ratmann, PhD	Imperial College London	Statistician and Trial Advisor
Seth Flaxman, PhD	University of Oxford	Statistician and Trial Advisor
Jamie Lachman, DPhil	University of Oxford	Trial Advisor

1.2 SAP revisions

SAP revisions				
SAP revision number	Date of revision	Timing of SAP revision in relation to analysis	Details of revision	Justification for revision

2. Signatures

Name	Signature	Date	Role
Sydney Tucker, MPH	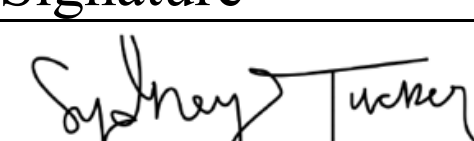	01.05.2024	Co-Investigator and Analyst
Susan Hillis, PhD	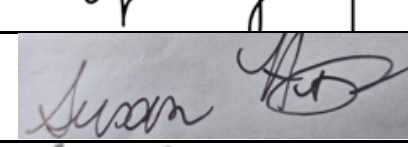	09.05.2024	Principal Investigator
Lucie Cluver, DPhil	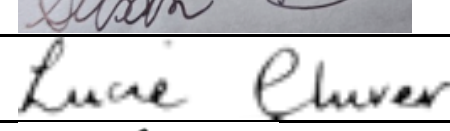	09.05.2024	Principal Investigator
G.J. Melendez-Torres, DPhil	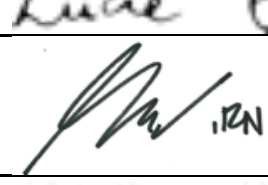	01.05.2024	Statistician and Trial Advisor
Oliver Ratmann, PhD	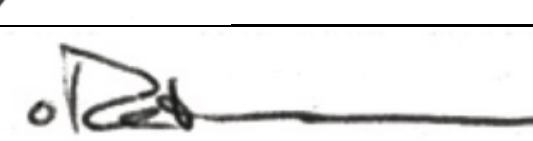	01.05.2024	Statistician and Trial Advisor

Seth Flaxman, PhD		01.05.2024	Statistician and Trial Advisor
Jamie Lachman, DPhil		02.05.2024	Trial Advisor

3. Introduction

3.1 Background

Over two billion people today live in countries affected by conflict, fragility, or violence.[1] More than two-thirds of the world's children lived in a conflict-affected country in 2021, and over one in six children lived within 50 kilometers of conflict zones.[2] There are more people displaced than ever before in history. [3] Specifically in Ukraine, within the first year of Russia's full-scale invasion in 2022, 13 million Ukrainians were displaced. Armed conflict and forced migration lead to both short-term and long-term ramifications on health and well-being, including mental health distress, post-traumatic stress disorder, complex grief, violence, and exploitation.[4,5] Children are among the most vulnerable.[6]

Therefore, within the first year after Russia's full-scale invasion, Ukrainians displaced by the war, World Without Orphans, Parenting for Lifelong Health (PLH), Oxford University, Children's Mission, Ukraine Without Orphans, and VIVA partnered to design a psychosocial, mental health, and parenting support group to strengthen caregivers and children affected by the war called *Hope Groups.* Results from a pre-post study in 2023 showed statistically significant improvements on caregiver mental health, parenting practices, violence against children, and child well-being at both midline and endline.[7] Thus, as a next step after promising pre-post results, we designed a randomized controlled trial (RCT) to evaluate the causal effect of 12-session *Hope Groups* of 5-7 Ukrainian parents and caregivers, led by Ukrainian peer-facilitators, compared to wait-list controls.

3.2 Trial Objectives

This RCT's primary objective is to evaluate the causal effect of 12-session *Hope Groups* among Ukrainian parents and caregivers affected by war, compared to a waitlist control group. Primary outcomes include caregiver mental health, violence against children, and positive parenting. Secondary outcomes include prevention of violence against women and additional caregiver and child overall well-being measures. We hypothesize *Hope Groups* will lead to improvements across all outcomes from baseline to endline (one-week post intervention), and we hypothesize improvements will be sustained within the intervention group at follow-up (6-8 weeks post intervention).

4. Study methods

4.1 Trial design

This is a pragmatic, facilitator-matched, cluster RCT with a parallel design and 1:1 allocation ratio to evaluate the effectiveness of *Hope Groups* compared to a wait-list control group, among 90 clusters of 4-7 Ukrainian parents and caregivers internally displaced, externally displaced, and living in war zones. The unit of randomization is each *Hope Group* of 4-7 participants.

4.2 Randomization

Cluster randomization matched on facilitator is employed using the treatment_assign function within the RCT package (v. 1.1.2) in R. Prior to study launch, facilitators elected to recruit and facilitate two or four Hope Groups. Randomization occurs after a facilitator completes recruitment for all of their clusters; after completion, facilitator-matched clusters are randomized

simultaneously using the treatment_assign function – which ensures allocation concealment is maintained.

4.3 Sample size

This study will enroll k=90 clusters of 4-7 participants, totaling approximately n=450 participants (assuming an average cluster size of m=5). The sample size was determined by power calculations from the pilot study and funding limitations. Based on estimated effect sizes, ICCs, and SDs of the outcome in the population from pilot data, and accounting for attrition in an active war context, we estimated that k=90 clusters and n=450 participants would be sufficient to detect significant effects with 80% power in all primary and secondary outcomes, except for physical abuse, which we may not be powered to detect an effect in. However, our funding limits increasing the sample size beyond this. Our final sample size may vary depending on actual cluster sizes compared to the assumed average above.

4.4 Framework

Statistical analyses will be undertaken within a superiority hypothesis testing framework.

4.5 Timing of outcome assessments

Outcomes will be assessed at baseline prior to randomization (T0) and approximately 1-week post-intervention (T1). Our initial Open Science Framework preregistration stated that researchers tentatively planned to conduct a follow-up evaluation (T2) 4-6-weeks after T1, but the final decision about this follow-up survey would be based on the state of circumstances surrounding the war in Ukraine and the level of urgency for psychosocial, parenting support among caregivers.

In January 2024, as missiles and bombs had continued in Ukraine for almost two years, causing mass devastation and compounding trauma, Ukrainian partners expressed urgency to deliver the *Hope Groups* to control clusters immediately after T1 rather than waiting through a follow-up period (T2), based on: 1) the war context having detrimental impacts on caregiver mental health (e.g., reports of increasing suicide among non-participant caregivers living in communities served by the program) and violence against children, which results in an urgent need for psychosocial, parenting support; 2) strong results from our pre-post study[7]; 3) concerns that high mobility during the war crisis may result in control participants' inability to participate if delayed, even through a short follow-up period. This was recognized and adopted by all study investigators, who agreed that efficacy of the intervention would evaluated at T1, rather than continuing the trial to T2. We still evaluate efficacy at T2 only within the intervention arm (see Section 7.3.2 'Primary analyses methods').

4.6 Statistical interim analyses

To enable individuals in conflict settings to benefit from demonstratable effective psychosocial interventions as rapidly as possible, we include real-time interim analysis methodology into this analysis plan. We use a Bayesian framework among the first 20 clusters (~100 participants) to reach T1 with the following guidelines:

1. If we find evidence of a harmful effect across primary outcomes, we will pause all activity to investigate and make adaptions.
2. If we find evidence of a protective effect of *Hope Groups* across primary outcomes, even after incorporation of skeptical priors[10,11, 12], this will be used to support the decision to deliver *Hope Groups* to control clusters after T1, rather than delaying them through a follow-up period timepoint (T2).
3. If we find evidence of a null effect, we will incorporate an enthusiastic prior[10,11, 12] based on information from the pre-post study[7]; if results remain null, we will still continue the trial until T1 to enable the sample size to reach the target number, but we will collaborate with Ukrainian partners to make adaptations to *Hope Groups* prior to delivery to control clusters, aiming to strengthen the intervention.

4.7 Stopping Guidance

Interim analysis results will not be used to consider stopping the trial prior to T1. As facilitators are logistically unable to deliver *Hope Groups* to intervention and control clusters simultaneously, they must first finish delivering *Hope Groups* to intervention clusters prior to continuing to deliver the intervention to control clusters – which makes delivery of the intervention to control clusters prior to T1 impossible. Rather, the purpose of the interim analysis is to explore the efficacy of *Hope Groups* to support delivering the intervention to control clusters after T1, as rapidly as possible as statistical evidence accrues.

4.8 Timing of final analysis

Final primary analysis will be conducted following completion of data collection across all clusters after T1, which we expect to be in April 2024. Primary analysis will be conducted using a frequentist framework, as originally specified in our preregistration.

5. Statistical Principles

5.1 Confidence intervals and p-values

All reported confidence intervals and Bayesian credible intervals will be at the 95% level. Any hypothesis testing will be undertaken at the level of statistical significance corresponding to $\alpha=0.05$ with two-tailed tests.

5.2 Intervention adherence and protocol deviations

Protocol deviations, if they occur, are likely to relate to changing needs in a war context – such as relocation of participants and/or facilitators or needing to alter the frequency of Hope Groups' sessions due to changes in circumstances. We have aimed to design this intervention and study to allow for all of these changes (e.g., groups can transition between virtual, in-person, and hybrid format and can meet 1x, 2x, or 3x per week depending on participant needs). However, if there are further changes that need to be made, we will update these changes both on our Open Science Framework preregistration and create an updated SAP version.

5.3 Analysis populations

Analysis is completed at the individual participant level, with random effects on their Hope Group ID to account for clustering in all models. Our primary analysis will be intention-to-treat

(ITT). If there is significant non-adherence, we will conduct both per-protocol analysis and instrumental variable analysis for exploration, and we will present these results with ITT results.

6. Trial population

6.1 Eligibility

Participant inclusion criteria are as follows: Participant 1) is Ukrainian, or another nationality and affected by the war in Ukraine; 2) understands the Ukrainian language 3) is aged 18 or older; and 4) is a parent or caregiver for one or more children ages 0-17 years old. Participant exclusion criteria: 1) Participants are not eligible if they have already participated in a *Hope Group*.

Facilitator inclusion criteria are as follows: Facilitator 1) is Ukrainian, or another nationality and affected by the war in Ukraine; 2) speaks and understands Ukrainian; 3) is 18 or older; 4) is connected to an NGO, refugee shelter, FBO, church, or other network of Ukrainians; 5) has had previous experience leading small group discussions; 6) self-defines as emotionally stable with capacity to support others; 7) is willing to participate in *Hope Groups* trainings; 8) and is willing to recruit and facilitate two or four *Hope Groups* through their network, with the understanding that their groups will be randomized to intervention or control with a 1:1 allocation ratio. There are no inclusion or exclusion criteria for facilitators' backgrounds; some facilitators are mental health professionals, while others have no previous mental health training.

6.2 Recruitment

There will be a rolling recruitment and enrollment strategy, with recruitment efforts ending when all facilitators have recruited the full number of participants for their groups. *Hope Groups* facilitators will recruit participants through their own networks (e.g., NGOs, shelters for internally displaced persons (IDPs) and/or refugees, FBOs, or churches) to participate in their 12-session *Hope Groups*. This approach to recruitment is based on the guidance of Ukrainian partners and humanitarian research experts.[24] It is especially important in humanitarian contexts for people to know and trust the organizations and people recruiting them for resources, especially in Ukraine, where psychosocial programming is historically uncommon. Therefore, to successfully achieve the target sample size, it is essential for participants to be recruited by someone they know and trust. Because of this programmatic necessity, our study design is matched on facilitator, to ensure all confounders within a facilitator's recruitment network are balanced between study arms.

Data to be reported in the CONSORT diagram are as follows:

- Recruited number of participants and clusters
- Cluster randomization to each arm
- Any participant(s) or cluster(s) that drop out after randomization

6.3 Withdrawal

Participants are told at the time of informed consent that they may withdraw at any time during the study, until after T2, when unique identifiers will be removed and replaced with a string of numbers.

6.4 Baseline characteristics

The following participant baseline characteristics are known:

- Age
- Gender
- Marital status
- Cohabitating with partner
- Education
- Income
- Displacement status (both current and any time since Russia's full-scale invasion)
- Country of current residence
- Location at the time of Russia's fullscale invasion (measured by country and Oblast, regions in Ukraine)
- Military service (both participant and their partner)
- Receipt of resources since Russia's full-scale invasion
- Number of children
- Relationship to children (parent or caregiver)

7. Statistical analysis

7.1 Primary outcomes

Following guidance for research in war settings, researchers made every effort to create a highly sensitive, non-triggering, and brief survey for participants.[24] To achieve this, we adapted validated scales to be relevant to our context, often including relevant subscale items rather than full survey scales. Primary outcomes are detailed in Table 1; all outcomes are based on participant self-report. All outcomes are collected at baseline (T0), endline (T1), and follow-up (T2).

Table 1. Primary Outcomes

Outcome	Measures	Source measure was adapted from	No. of items	Included in Interim Analysis	Range
**Caregiver Mental Health**	**Depression and Anxiety**	Patient Health Questionnaire-4	4	No	0-12**
	**Depression**	CES-D Depression Screening Tool	1	Yes	0-7*
**Violence Against Children**	**Physical Violence; Emotional Violence**	International Society for the Prevention of Child Abuse and Neglect Screening Tool – Trial (ICAST-T): Items from Physical Abuse and Emotional Abuse subscales	2	Yes	0-7*
	**Nonviolent Discipline**	Parenting Young Children: Setting Limits Subscale	1	Yes	0-7*
**Parenting**	**Reinforcing Positive Behavior; Supporting Child Development through Play**	Alabama Parenting Questionnaire (APQ): Positive Parenting subscale	2	Yes	0-7*
	**Supporting Child Development Through Learning; Meaningful Conversations with Child**	Alabama Parenting Questionnaire (APQ): Parental Involvement subscale	2	Yes	0-7*
	**Monitoring Child; Protecting Child**	Alabama Parenting Questionnaire: Poor Supervision	2	Yes	0-7*

* Survey questions asked for frequency of occurrence in number of days in the past week. When multiple items are used, responses are averaged together to result in a range still between 0-7.
** Survey questions are on the following ordinal scale to indicate occurrence in the past week: Not at all, Several days, More than half the days, Nearly every day

7.2 Secondary outcomes

Consistent with our approach for primary outcomes, we also adapted validated scales to be relevant to our context for our secondary outcomes, often including relevant subscale items rather than full survey scales. Secondary outcomes are detailed in Table 2; all outcomes are based on participant self-report. All outcomes are collected at baseline (T0), endline (T1), and follow-up (T2), except for Violence Against Women outcomes, which will only be asked at endline (T1) and follow-up (T2) based on guidance from Ukrainian partners, due to their highly sensitive nature. In addition to the Secondary Outcomes items in this table, we will also assess

'Violence Against Children' and 'Parenting' measures from the above Primary Outcomes table individually as secondary outcomes.

Table 2. Secondary Outcomes

Outcome	Measures	Source measure was adapted from	No. of items	Range
**Caregiver Resilience and Well-being**	**Coping with Grief; Hopefulness about the future**	Based on the Connor-Davidson Resilience Scale 2;	2	0-7*
	**Self-care Practices**	Center for Epidemiological Studies Depression	1	0-7*
**Child Well-being**	**Internalizing Behavior; Externalizing Behavior; Depression**	Child and Adolescent Behavior Inventory - Internalizing Symptoms, Externalizing Symptoms, Depression Symptoms	3	0-7*
**Prevention of Violence Against Women**	**Prevention of Intimate Partner Violence and Sexual Violence**	Adapted from Key Concepts in No Means No Worldwide	2	0-8**
	**Healthy Relationship Building**	Created by team for Ukrainian context	1	0-7*

* Survey questions asked for frequency of occurrence in number of days in the past week. When multiple items are used, responses are averaged together to result in a range still between 0-7.
** Survey questions are on the following ordinal scale to indicate confidence: Not at all confident, Slightly confident, Somewhat confident, Fairly confident, Completely confident.

7.3 Descriptive Statistics

Before undertaking the primary analysis, we will examine the mean, median, range, and distribution of all variables at baseline and endline (T1). We will prepare a table of summary statistics including proportions for categorical variables and mean (including standard deviation) and median (including interquartile range) for all continuous variables at baseline. We will also examine patterns of missingness for all outcomes at item- and scale- level. Histograms and summary statistics will confirm the choice of regression model. (See '7.4.2 Primary Analysis Methods' below for more details)

7.4 Analysis methods

In both the interim analysis and the final analysis, the primary method of analysis will be a two-level generalized linear mixed-effects model, which will nest person within cluster, and control for baseline outcome measures. To account for matching, we include a fixed effect on the multi-categorical variable for facilitator. We will use an intention-to-treat approach with a binary exposure variable indicating if the participant was randomized to the intervention or control group.

7.4.1 Interim analysis methods

Specifically for the interim analysis, we will use rstanarm (v. 2.32.1) in R to implement our primary and secondary statistical model reported above using a Bayesian approach. The wartime situation makes it a priority to translate trial findings into practice; for this reason, analysis methods will incorporate continuous interim analyses for efficacy, and such continuous assessments are seamlessly straightforward within a Bayesian analysis framework.[8,9] Steps will be as follows:

1. We will first utilize a weakly informative prior on the treatment effect centered at 0 with a standard deviation of 2.5 (variance of 6.25) to assess if *Hope Groups* demonstrate a significant effect across outcomes indicated above, as measured by quantifying if posterior credible intervals of the treatment effect include the null with a probability of less than 5%.

2. To conduct interim analyses conservatively, if any positive intervention effects are detected, we will incorporate a skeptical prior on the treatment effect, which is centered at 0 with substantially stronger precision than under point (1) above to assert a skeptical belief about the intervention having an effect[10], and assess if the significant effect(s) remain. This approach ensures that evidence needs to be overwhelming for the treatment effect to be called significant in interim analyses. To calculate the skeptical prior, we use the following formula:[11,12]

   $$prior: f(\theta) \sim \mathcal{N}(0, \sigma^2/m_0)$$
   $$m_0 = (Z_\gamma \sigma/\theta_s)^2$$

   where Z_γ is the upper quantile of the standard normal (1.645 at α = .05), σ is the standard deviation, and θ_s is the estimated treatment effect.

   In our pre-post study[7], the largest standard deviation across primary outcomes was 2.85; to be conservative, we set σ=3.0. The smallest treatment effect across primary outcomes in the pre-post study was 1.13[7]; to be conservative and assume a 10-15% placebo effect from the pre-post study, we reduced this θs=1.0 – which also corresponds to the minimum treatment effect Ukrainian partners stated would make the intervention worth implementing. Thus, m_0 = 24.35. Our final skeptical prior is:

   $$prior: \ f(\theta) \sim \mathcal{N}\ (0, 0.37)$$

   i.e. a normal distribution with a variance of 0.37 (equivalently a standard deviation of .61).

   If any mental health, parenting, or violence against children outcomes remain significant after incorporation of the skeptical prior, we consider there to be sufficient evidence to support providing *Hope Groups* to the control group immediately after trial endline (T1) without delaying them through a follow-up period (T2), in light of the war context having detrimental impacts on caregiver mental health, safety, and violence against children,

especially due to high mobility in populations which may result in participants' inability to participate if delayed through a follow-up period. We selected mental health, parenting, and/or violence against children outcomes due to their potential for acute impact on both the short-term and long-term health of caregivers and children.

3. If harmful intervention effects are detected in the weakly informative interim analysis, we will pause trial activity to investigate and make adaptations.

4. If results are null in the weakly informative interim analysis, we will also incorporate an enthusiastic prior[10,11, 12] based on information from the pre-post study[7], centered on 1 to assert an enthusiastic belief about the intervention's effect remaining consistent with pre-post results, with a standard deviation of 3. If results remain null, the trial will continue through T1 to enable the sample size to reach the target amount, but we will collaborate with Ukrainian partners to make adaptations to *Hope Groups* prior to delivery to control clusters, and adapted *Hope Groups* will be assessed using a pre-post design within the control clusters.

7.4.2 Primary analysis methods

Primary analyses will be undertaken using a frequentist approach, as originally pre-specified on our OSF preregistration. The primary method of analysis will be a two-level generalized linear mixed-effects model, which will nest person within cluster and control for baseline outcome measures; baseline measures will be centered at the sample mean. To account for matching, we include a fixed effect on the multi-categorical variable for facilitator. We will use an intention-to-treat approach with a binary exposure variable indicating if the participant was randomized to the intervention or control group. We will additionally include the covariate of displacement status at baseline in all models, as this was shown to be a key predictor in outcome levels in the pre-post study, with a set referent group of living at-home. The regression link function will be based on consideration of outcome distributions at baseline, and the appropriate link function will be used for each outcome: Where variables are continuous and an identity link function appears appropriate, mean differences will be estimated. Where an ideal link function is not available (e.g., limited range variables where linear, or ordered logit links are inappropriate), we will use wild bootstrapping[14] with an identity link, and mean differences will be estimated. We will use two-tailed tests for all models with statistical significance thresholds of 0.05; where a bootstrap method is used, percentile-based 95% confidence intervals will be reported. In the event that mixed-effects models are not estimable or do not converge, we will revert from a mixed-effects model to a 'flat file' model with standard errors clustered at the level of each Hope Group.

Additionally, we will perform our primary analysis using a Bayesian approach, as used for the interim analysis, for exploratory purposes of assessing if results remain consistent.

Lastly, we will investigate efficacy at T2 in the intervention group, as compared to efficacy at T1 in the intervention group. We recognize that the RCT only compares efficacy between trial arms at T1; efficacy at T2 is assessed using an observational design (due to the control group receiving the intervention after T1, see section 4.5 for full details). The analysis of follow-up (T2) data within the intervention group only will use a three-level model with measurement wave within person within group. To assess stability of change over time, we will use a 'time-differenced' approach with time terms corresponding from baseline to follow-up (T2) and from endline (T1) to follow-up (T2).

7.4.3 Exploratory analysis methods

We will explore differences in the effectiveness of *Hope Groups* by variables pertaining to both participant characteristics and intervention delivery.

Participant Characteristics:

1. Displacement status: Three-level categorical variable measured at an individual participant level (Internally displaced; Externally displaced; and Living at home)
2. Shelter status: Binary variable measured at individual participant level (Living in shelter; Not living in shelter)
3. Education level: Categorical variable measured at an individual participant level
4. Child disability status: Binary variable indicating whether or not a parent/caregiver has a child with a disability, measured at an individual participant level
5. Baseline-level of outcome: Ordinal or continuous variable using baseline outcome measures (as displayed in tables above), at an individual participant level.
6. Proximity to active war: Three-level categorical variable measured at the individual level indicating war activity (Frequent attacks; infrequent attacks; no attacks)
7. Number of children: Continuous variable measured at an individual participant level

To gain insight into whether there are unique subgroups of participants who experience greater benefits from Hope Groups, we will assess alterations in Hope Groups' effectiveness by each of these participant-level exploratory variables. We will interact the exploratory variable with the treatment variable using our existing primary generalized linear mixed-effects models.

Intervention Delivery:

1. Participation format (in-person, virtual, hybrid): Three-level categorical variable measured at both the individual level and cluster level
2. Facilitator background (lay-trained or mental health professionals): Binary variable measured at a cluster level
3. Frequency of delivery (once, twice, or three times per week): Categorical variable measured at a cluster level
4. Facilitator network: Categorical variable measured at a cluster level indicating if groups were recruited through friend/family networks, NGO/shelter networks, or church/FBO networks

To gain insight into whether there are specific delivery circumstances that yield greater benefits from Hope Groups, we will assess alterations in Hope Groups' effectiveness by each of these delivery-based exploratory variables. We will construct 5 separate models to assess differences in

the treatment effect by: participation format categorized at the individual level, participation format categorized at the cluster level, facilitator background, frequency of delivery, and facilitator network. We will use test of equality coefficients to establish differences in effects of the intervention.

Specifically for the follow-up analysis among the intervention arm, if we see outcomes worsening from endline (T1) to follow-up (T2), we will explore stratifying the data by participants who did and did not report worsening circumstances due to the war since their Hope Group completed and see if results differ. Additionally, we will explore stratifying the data by level contact participants have engaged in with their facilitator and fellow-participants since completion of their Hope Group (mid-high contact vs low-no contact), to gain insight on if continued contact may be important for sustained outcomes.

Lastly, we will examine whether cluster size impacted intervention effectiveness, using an approach consistent with Kahan et. al. (2023) to assess the presence of informative cluster size.[13]

7.4.4 Missingness

If any outcome variable missingness is over 10%, we will use multiple imputation for outcome variables where scale scores cannot be rescaled, using a two-level multilevel model without accounting for pair matching. Additionally, if any cluster has a combination of missingness that would result in the dropping of a cluster, we will use an impute-then-boot strategy to impute missing values within that cluster to prevent exclusion of a cluster, regardless of whether the total missingness exceeds 10%.

7.4.5 Cost effectiveness analysis methods

We will additionally explore the cost-effectiveness of Hope Groups. This analysis will take into account facilitator income and reimbursement amounts (travel expenses), time spent with participants both in sessions and outside of sessions, and the time and income of coordinators supporting facilitators. A separate statistical analysis plan will be written and posted to OSF.

7.5 Statistical Software

All data cleaning and analysis will be completed in R.

7.6 Harms

Fatal and life-threatening events (otherwise known as serious adverse events (AE)) experienced by participants while participating in the study are unlikely to be caused by participation in this trial. Nonetheless, any adverse events reported during the duration of this study will be reported to ethical review boards who approved this study.

7.7 Quality assurance

As a minimum, derivation of each of the primary outcomes will be undertaken twice, with primary analyses also performed twice. Dependent on capacity, derivation of key secondary outcomes will also be undertaken twice, independently where possible.

References

1. Social Cohesion and Resilience. *World Bank* https://www.worldbank.org/en/topic/social-cohesion-and-resilience.
2. Østby, Gudrun; Siri Aas Rustad & Andrew Arasmith (2022) Children Affected by Armed Conflict, 1990–2021, Conflict Trends, 2. Oslo: PRIO. - Google Search. https://www.google.com/search?q=%C3%98stby%2C+Gudrun%3B+Siri+Aas+Rustad+%26+Andrew+Arasmith+(2022)+Children+Affected+by+Armed+Conflict%2C+1990%E2%80%932021%2C+Conflict+Trends%2C+2.+Oslo%3A+PRIO.&oq=%C3%98stby%2C+Gudrun%3B+Siri+Aas+Rustad+%26+Andrew+Arasmith+(2022)+Children+Affected+by+Armed+Conflict%2C+1990%E2%80%932021%2C+Conflict+Trends%2C+2.+Oslo%3A+PRIO.&gs_lcrp=EgZjaHJvbWUqBggAEEUYOzIGCAAQRRg70gEHNDE1ajBqN6gCALACAA&sourceid=chrome&ie=UTF-8.
3. 110 million people displaced around the world: get the facts | International Rescue Committee (IRC). https://www.rescue.org/uk/article/110-million-people-displaced-around-world-get-facts (2023).
4. The longitudinal impact of war exposure on psychopathology in Syrian and Iraqi refugee youth - Liza Marie-Emilie Hinchey, Raya Nashef, Celine Bazzi, Kathleen Gorski, Arash Javanbakht, 2023. https://journals.sagepub.com/doi/10.1177/00207640231177829?url_ver=Z39.88-2003&rfr_id=ori:rid:crossref.org&rfr_dat=cr_pub%20%200pubmed.
5. Barenbaum, J., Ruchkin, V. & Schwab-Stone, M. The psychosocial aspects of children exposed to war: practice and policy initiatives. *J. Child Psychol. Psychiatry* **45**, 41–62 (2004).

6. Hillis, S. D., Mercy, J. A. & Saul, J. R. The enduring impact of violence against children. *Psychol. Health Med.* **22**, 393–405 (2017).

7. Hillis, S. *et al.* The Effectiveness of Hope Groups, a Mental Health, Parenting Support, and Violence Prevention Program for Families Affected by the War in Ukraine: Findings from a Pre-Post Study. SSRN Scholarly Paper at https://doi.org/10.2139/ssrn.4677018 (2024).

8. An Overview of the Bayesian Approach. in *Bayesian Approaches to Clinical Trials and Health-Care Evaluation* 49–120 (John Wiley & Sons, Ltd, 2003). doi:10.1002/0470092602.ch3.

9. Randomised Controlled Trials. in *Bayesian Approaches to Clinical Trials and Health-Care Evaluation* 181–249 (John Wiley & Sons, Ltd, 2003). doi:10.1002/0470092602.ch6.

10. Bendtsen, M. Avoiding Under- and Overrecruitment in Behavioral Intervention Trials Using Bayesian Sequential Designs: Tutorial. *J. Med. Internet Res.* **24**, e40730 (2022).

11. Parmar, M. K., Spiegelhalter, D. J. & Freedman, L. S. The CHART trials: Bayesian design and monitoring in practice. CHART Steering Committee. *Stat. Med.* **13**, 1297–1312 (1994).

12. Prior Distributions. in *Bayesian Approaches to Clinical Trials and Health-Care Evaluation* 139–180 (John Wiley & Sons, Ltd, 2003). doi:10.1002/0470092602.ch5.

13. Kahan BC, Li F, Blette B, Jairath V, Copas A, Harhay M. Informative cluster size in cluster-randomized trials: A case study from the TRIGGER trial. Clinical Trials. 2023;20(6):661-669. doi:10.1177/17407745231186094

14. Modugno, L., & Giannerini, S. (2015). The Wild Bootstrap for Multilevel Models. *Communications in Statistics - Theory and Methods*, *44*(22), 4812–4825. https://doi.org/10.1080/03610926.2013.802807
