# Supplementary material for: Effectiveness of the hope groups intervention on mental health, parenting practices, and violence against children among Ukrainian parents and caregivers affected by war and displacement: a cluster randomised controlled trial
Source: Lancet Reg Health Eur. 2026 Aug 25;68:101783. doi: 10.1016/j.lanepe.2026.101783 (PMC13559926; doi:10.1016/j.lanepe.2026.101783)
Supplement: Supplementary file 4 [file mmc1.docx]

**Supplementary Materials**

**Supplementary Table 1. Description of 12-Session *Hope Groups* Intervention**

| **Session** | **Title** | **Content** |
| --- | --- | --- |
| 1 | **Finding Stability - Our Everyday Tools** | This session builds skills on strategies for building hope in transition and crisis; we create discussion around the impacts of trauma and equip participants to begin building a personal “toolbox” for responding to crisis. |
| 2 | **Finding Stability - Our Anchoring Tools** | This session focuses on common physical, emotional, behavioural, and cognitive reactions to trauma among caregivers and children. We build participant skills in developing stabilisation techniques, such as deep breathing and progressive muscle relaxation, for coping with stress. |
| 3 | **Talk About It** | This session builds skills in identifying and sharing emotions participants are experiencing related to war, displacement, and crisis. Parents also build skills in how to talk with children about war and loss to increase positive parent-child communication, strengthen parent-child relationships, support the well-being of children, and ultimately aim to prevent short- and long- term child behavioural issues. |
| 4 | **Strong Families** | This session builds skills on developing and maintaining strong relationships within families, particularly caregiver/child relationships. Skills are built for positive communication within relationships and equipping parents to use positive parenting practices, such as positive praise, fun parent-child activities (e.g., child-led play), and daily routines to reinforce positive behaviour, support child development, and strengthen parent-child relationships, even during war, displacement, and crisis. |
| 5 | **Staying Safe Together** | This session builds skills and tools for staying safe during crises and war, including being physically together as a family, when possible, and awareness of the risks and signs of sexual perpetrators and human trafficking. Participants practice learning to talk with their children about abuse and trafficking in age-appropriate terms and respond empathetically if children disclose experiencing abuse. Participants are also equipped with parenting skills such as supervision and monitoring to safeguard their children’s childhoods amidst crisis. |
| 6 | **Staying Safe at Home** | This session builds parental emotional regulation skills to respond to strong emotions, such as anger, in healthy, safe ways even in stressful moments. Participants are equipped to use positive parenting practices (e.g., reinforcing positive behaviours) and nonviolent discipline (e.g., time out, removing privileges, talking with children about why what they did was wrong), with the aim of reducing harsh discipline and violence against children. |
| 7 | **Coping with Loss** | This session teaches participants about the stages of grief, with an aim to normalise and remove stigma from the concept of grieving. Participants build skills for coping with trauma, healthy grieving, and finding meaning in their new reality. Participants are also equipped to discuss these topics with their children (e.g., child-appropriate activities to help their children honour whom/what they lost). Participants are taught that children who are grieving may often misbehave, as to increase parental awareness and empathy regarding common child behaviours. Parents/caregivers are equipped with skills to provide comfort and support to children coping with loss and strengthen parent-child relationships. |
| 8 | **Building Hope through Understanding Guilt and Secondary Trauma** | This session equips participants to understand ‘survivor guilt’ and ‘secondary trauma’. Facilitators validate the stress of war, displacement, and crisis. Participants learn that children, too, may experience these reactions and are equipped with positive parenting skills and child-friendly activities to support children’s well-being. |
| 9 | **Learning Together** | This session builds positive parenting skills for caregivers to support their children’s learning, amidst war, displacement, and crisis, when many children have left their former schools and are attending new schools or are studying at home/online without a traditional education environment. Participants are equipped to support children’s learning through available resources, age-appropriate household and family responsibilities, child-led play, and creating realistic routines in crisis. |
| 10 | **Healthy Relationships** | This session builds skills for maintaining relationships with loved ones long-distance (e.g., making time for enjoyable connection even amidst war, displacement, and crisis) and healthy sexual decisions. Differences between healthy and unhealthy relationships are clarified, especially during times of stress and physical separation. Participants learn how to discuss these topics appropriately with older children and strengthen children to recognise and respond to pressure from peers and maintain personal boundaries in relationships. |
| 11 | **Staying Safe in our Relationships** | This session builds skills in saying ‘No’ to unwanted sexual activity. We discuss sexual consent, coercion, verbal and physical skills to say ‘no’, and options for protecting against sexually transmitted infections and unwanted pregnancy. We briefly explore how post-traumatic stress disorder (PTSD) among active military personnel and veterans can impact behaviour and relationships. Participants learn how to discuss these topics appropriately with older children and strengthen children to recognise and respond to pressure from peers and maintain personal boundaries in relationships. |
| 12 | **Resilience** | This final session builds skills in exploring emotions and discerning how to continually care for their own mental health, as well as their children’s, as they build hope for the future amidst the war, displacement, and crisis. Participants are equipped with 1) activities to teach children how to express their emotions, including difficult emotions and 2) skills to respond to children’s emotions with empathy, providing a supportive presence to listen to, normalise, and value the emotions experienced by children amidst war, displacement, and crisis. |

**Supplementary Figure 1. Hope Groups Theory of Change**

**Hope Groups Theory of Change**, first published in our pre-post paper^16^, shows that the intervention aims to strengthen caregiver mental health, parenting skills, and child protection skills, all of which may be adversely affected by war exposures and displacement stressors. By strengthening caregiver mental health, parenting and child protection skills, the conceptual model shows that key aims are to reduce violence against children, and to improve both children’s and caregivers’ wellbeing.

**Supplementary Table 2. Primary Outcomes**

| **Outcome** | **Measures** | **Source measure was adapted from** | **No. of items** | **Range** | **Intraclass correlation coefficient** |
| --- | --- | --- | --- | --- | --- |
| **Caregiver Mental Health** | **Depression and Anxiety** | Patient Health Questionnaire-4 | 4 | 0-12** | 0·085 |
| **Violence Against Children** | **Physical Violence;**  **Emotional Violence** | International Society for the Prevention of Child Abuse and Neglect Screening Tool – Trial (ICAST-T): Physical Abuse and Emotional Abuse subscales | 2 | 0-7 days* | 0·068 |
| **Parenting** | **Positive Parenting, Parental Involvement, and Parental Monitoring** | Alabama Parenting Questionnaire (APQ) | 6 | 0-7 days* | 0·134 |

## ** Survey questions asked for frequency of occurrence in number of days in the past week. When multiple items are used, responses are averaged together to result in a range still between 0-7.*

*** Survey questions are on the following ordinal scale to indicate occurrence in the past week: Not at all, Several days, More than half the days, Nearly every day.*

**Supplementary Table 3. Secondary Outcomes**

| **Outcome** | **Measures** | **Source measure was adapted from** | **No. of items** | **Range** |
| --- | --- | --- | --- | --- |
| **Caregiver Hopefulness about the Future and Coping with Grief** | **Hopefulness about the future; Coping with Grief.**  **Self-care Practices** | Question on hopefulness about the future is from Centre for Epidemiological Studies Depression. Question on coping with grief is measured via utilisation of healthy practices to mourn losses and move forward, which is adapted from the idea of resiliently “bouncing back” from Connor-Davidson Resilience Scale 2.  Centre for Epidemiological Studies Depression | 2  1 | 0-7 days*  0-7 days* |
| **Child Behavioural Issues** | **Internalising and Externalising Behaviour** | Child and Adolescent Behaviour Inventory - Internalising Symptoms, Externalising Symptoms, Depression Symptoms | 3 | 0-7 days* |
| **Prevention of Violence Against Women** | **Prevention of Intimate Partner Violence and Sexual Violence**    **Healthy Relationship Building** | Adapted from Key Concepts in No Means No Worldwide  Created by team for Ukrainian context | 2  1 | 0-8**  0-7 days* |
| **Parenting (Individually by Subscale)** | **Positive Parenting: Reinforcing Positive Behaviour; Supporting Child Development through Play  Parental Involvement: Supporting Child Development Through Learning; Meaningful Conversations with Child about their Worries and Emotions   Parental Monitoring: Monitoring and Protecting Child** | Alabama Parenting Questionnaire (APQ): Positive Parenting subscale    Alabama Parenting Questionnaire (APQ): Parental Involvement subscale    Alabama Parenting Questionnaire (APQ): Poor Supervision | 2       2        2 | 0-7 days*       0-7 days*        0-7 days* |
|  | **Nonviolent Discipline** | Parenting Young Children: Setting Limits Subscale | 1 | 0-7 days* |

## ** Survey questions asked for frequency of occurrence in number of days in the past week. When multiple items are used, responses are averaged together to result in a range still between 0-7.*

*** Survey questions are on the following ordinal scale to indicate confidence: Not at all confident, Slightly confident, Somewhat confident, Fairly confident, Completely confident.*

**Supplementary Table 4: Bayesian Interim Analysis Intention-to-Treat Results, Estimating Absolute Treatment Effects and 95% Credible Intervals**

|  | **Outcome** | **Median (95% Credible Interval)** |
| --- | --- | --- |
| **Bayesian Interim Analysis: Neutral Priors^1^** | | |
|  | **Caregiver Depression and Anxiety**^†^ | -2·31 (-2·88, -1·68) ^† *^ |
|  | **Violence Against Children**^†^ | -0·59 (-0·86, -0·32)^*^ |
|  | **Parenting**^†^ | 1·91 (1·31, 2·47)^*^ |
| **Bayesian Interim Analysis: Sceptical Priors^2^** | | |
|  | **Caregiver Depression and Anxiety ^†^** | -1·76 (-2·39, -0·81) **^†*^** |
|  | **Violence Against Children**^†^ | -0·56 (-0·82, -0·30)^*^ |
|  | **Parenting**^†^ | 1·50 (0·70, 2·07)^*^ |

**^†^ The caregiver depression and anxiety measure is not the same measure used in the primary trial analysis. Thus, this result should not be compared with Table 2 in the manuscript.** *As explained in our registered statistical analysis plan, in the primary trial analysis, we used an improved full validated scale for caregiver mental health and anxiety, which had not been collected in the pre-post study. For the interim analysis, we used the shortened measure for caregiver mental health which was used in our pre-post study, as the estimation of sceptical priors is improved with reliable past data for estimates.*

**^*^Indicates the credible interval does not include the null value of 0.**

**Supplementary Table 5. Table of Safety**

|  | **Number of Events** | | | **Risk Difference (95% CI) *(intervention arm versus wait-list control arm)*** |
| --- | --- | --- | --- | --- |
|  | Intervention Participants During Intervention (N) | Control Participants While on Waitlist (N) | Control Participants After Trial When Receiving the Intervention (N) |  |
| Harms, Adverse Events, or Semi Adverse Events from the intervention | 0 | 0 | 0 | 0 (-0.015, 0.015) |
| Mandated reporting (eg, disclosure of child abuse) | 0 | 0 | 0 | 0 (-0.015, 0.015) |
| Deaths during trial | 0 | 0 | 0 | 0 (-0.015, 0.015) |

**Note: Waitlist control participants were monitored both while on the waitlist and after the trial concluded, when they received the intervention.**

**Supplementary Table 6. Intention-to-Treat Treatment Effects of Hope Groups Among Ukrainians Affected by War at Trial Endline: After the 12-Session Intervention (2023-2024)**

| **Type** | **Outcome** | **Intervention** | | **Control** | | **N** | **Absolute Treatment Effect**  β *(95% CI)* | **Percent Change %** *(95% CI)* | **P-**  **value** | **d** |
| --- | --- | --- | --- | --- | --- | --- | --- | --- | --- | --- |
|  |  | Base-  line  Median (25%, 75%) | End-  line  Median (25%, 75%) | Base-  line  Median (25%, 75%) | End-  line  Median (25%, 75%) |  |  |  |  |  |
| **Primary** |  |  | |  | |  |  |  |  |  |
|  | **Caregiver Depression and Anxiety** | 6·0  (4·0, 8·0) | 2·0 (0·0, 4·0) | 5·0 (4·0, 8·0) | 4·0 (3·0, 8·0) | 496 | -2·85 (-3·49, -2·22)^*^ | -57·4% (-65·1%, -48·5%) | <·0001 | -0·87 |
|  | **Violence Against Children** | 1·0 (0·5, 2·0) | 0·5 (0·0, 0·5) | 1·0 (0·5, 2·0) | 0·5 (0·5, 1·5) | 491 | -0·5 (-0·64, -0·36)^†^ | -52·6% (-64·7%, -36·5%) | <·0001 | -0·43 |
|  | **Parenting Practices** | 3.83 (2.33, 5.33) | 6.17 (5.50, 6.83) | 3.83 (2.33, 5.50) | 4.50 (3.17, 5.83) | 459 | 1·32 (0·92, 1·71) | 33·07% (24%, 44%) | <·0001 | 0·57 |
| **Secon-**  **dary** |  |  | |  | |  |  |  |  |  |
|  | **Caregiver Well-being** |  | |  | |  |  |  |  |  |
|  | Hopefulness about the Future and Coping with Grief | 2·00 (1·00, 3·50) | 5·50 (4·00, 6·50) | 2·00 (1·00, 3·50) | 3·00 (1·62, 4·00) | 490 | 1·82 (1·38, 2·25)^†^ | 65·43% (46·9%, 87·34%) | <·0001 | 0·97 |
|  | Caregiver Self Care | 2·00 (0·00, 3·00) | 6·00 (4·00, 7·00) | 1·00 (0·00, 3·00) | 3·00 (1·00, 4·00) | 489 | 2.21 (1·71, 2·71)^†^ | 84·69% (60·3%, 114·35%) | <·0001 | 1·04 |
|  | **Child Well-being** |  | |  | |  |  |  |  |  |
|  | Child Behavioural Issues: Internali-  sing and  Externali-  sing behaviours | 2·00 (1·00, 3·33) | 0·67 (0·00, 1·67) | 2·00 (1·00, 3·33) | 2·00 (1·00, 3·33) | 478 | -1·11 (-1·43, -0·79)^†^ | -56·1% (-64·5%, -46·0%) | <·0001 | -0·66 |
| *(Female only)* | **Prevention of Violence Against Women** | NA | 6·00 (5·00, 8·00) | NA | 3·00 (2·00, 6·00) | 477 | 2·26 (1·7, 2·82)^*^ | 68·05% (47·9%, 93·14%) | <·0001 | 0·93 |
| *(Married or partnered only)* | **Healthy Relation-**  **ship Building** | 2·00 (0·00, 4·00) | 6·00 (5·00, 7·00) | 2·00 (1·00, 5·00) | 3·00 (2·00, 5·00) | 322 | 2·11 (1·59, 2·62)^†^ | 64·71% (40·4%, 96·21%) | <·0001 | 0·83 |
|  | **Parenting (Subscales De- composed)** |  |  |  |  |  |  |  |  |  |
|  | Nonviolent Discipline | 3·00 (1·00, 6·00) | 6·00 (5·00, 7·00) | 3·00 (1·00, 6·00) | 3·00 (2·00, 5·00) | 493 | 1·45 (1·02, 1·88)^†^ | 43·33% (28·6%,  60·3%) | <·0001 | 0·6 |
|  | Positive Parenting | 3·25 (2·00, 5·50) | 6·00 (5·00, 7·00) | 3·50 (2·00, 5·50) | 4·00 (3·00, 6·50) | 487 | 1·2 (0·79, 1·61)^†^ | 31·77% (20·9%, 43·85%) | <·0001 | 0·55 |
|  | Parental Monitoring | 5·00 (2·75, 7·00) | 7·00 (6·50, 7·00) | 5·50 (3·00, 7·00) | 5·50 (3·50, 7·00) | 483 | 1·18 (0·74, 1·62)^†^ | 25·42% (15·7%, 36·46%) | <·0001 | 0·49 |
|  | Parental Involve-  ment | 3·00 (2·00, 4·50) | 6·00 (4·50, 7·00) | 3·00 (1·50, 4·00) | 3·50 (2·50, 4·50) | 486 | 1·6 (1·21, 1·97)^†^ | 45·65% (32·1%, 60·49%) | <·0001 | 0·87 |

^*^ *Treatment effect indicates change on Likert frequency scale (e.g., ‘not at all’ to ‘nearly every day’)*

^†^ *Treatment effect indicates change of outcome measured via days of occurrence within the past week (0-7)*

**Supplementary Table 7. Demographic Characteristics Among Ukrainian Participants Randomised to the Intervention Arm, Stratified by Programme Completion**

|  | **Did Not Complete All 12 Sessions (N=67)** | **Completed All 12 Sessions (N=188)** | **Overall (N=255)** | **p-value** |
| --- | --- | --- | --- | --- |
| **Sex** |  |  |  | 0.798 |
| Female | 59 (88.1%) | 170 (90.4%) | 229 (89.8%) |  |
| Male | 8 (11.9%) | 17 (9.0%) | 25 (9.8%) |  |
| Missing | 0 (0%) | 1 (0.5%) | 1 (0.4%) |  |
| **Age** |  |  |  | 0.401 |
| 20 - 29 | 7 (10.4%) | 17 (9.0%) | 24 (9.4%) |  |
| 30 -39 | 28 (41.8%) | 81 (43.1%) | 109 (42.7%) |  |
| 40 - 49 | 17 (25.4%) | 70 (37.2%) | 87 (34.1%) |  |
| 50 - 59 | 7 (10.4%) | 14 (7.4%) | 21 (8.2%) |  |
| Over 60 | 5 (7.5%) | 6 (3.2%) | 11 (4.3%) |  |
| Under 20 | 2 (3.0%) | 0 (0%) | 2 (0.8%) |  |
| Missing | 1 (1.5%) | 0 (0%) | 1 (0.4%) |  |
| **Marital Status** |  |  |  | 0.446 |
| Divorced | 10 (14.9%) | 22 (11.7%) | 32 (12.5%) |  |
| Married | 47 (70.1%) | 113 (60.1%) | 160 (62.7%) |  |
| Single | 2 (3.0%) | 27 (14.4%) | 29 (11.4%) |  |
| Unmarried with Partner | 4 (6.0%) | 18 (9.6%) | 22 (8.6%) |  |
| Widowed | 4 (6.0%) | 8 (4.3%) | 12 (4.7%) |  |
| **Education** |  |  |  | 0.553 |
| Primary School | 11 (16.4%) | 15 (8.0%) | 26 (10.2%) |  |
| Technical Degree | 17 (25.4%) | 60 (31.9%) | 77 (30.2%) |  |
| Bachelors Degree | 8 (11.9%) | 34 (18.1%) | 42 (16.5%) |  |
| Graduate Degree | 13 (19.4%) | 42 (22.3%) | 55 (21.6%) |  |
| Specialist | 18 (26.9%) | 36 (19.1%) | 54 (21.2%) |  |
| Missing | 0 (0%) | 1 (0.5%) | 1 (0.4%) |  |
| **Income** |  |  |  | 0.966 |
| Low Income | 36 (53.7%) | 108 (57.4%) | 144 (56.5%) |  |
| Middle Income | 29 (43.3%) | 72 (38.3%) | 101 (39.6%) |  |
| High Income | 2 (3.0%) | 5 (2.7%) | 7 (2.7%) |  |
| Missing | 0 (0%) | 3 (1.6%) | 3 (1.2%) |  |
| **Displacement Status** |  |  |  | 0.0154 |
| Internally Displaced | 14 (20.9%) | 51 (27.1%) | 65 (25.5%) |  |
| Living At-Home in Ukraine | 41 (61.2%) | 70 (37.2%) | 111 (43.5%) |  |
| Externally Displaced | 12 (17.9%) | 67 (35.6%) | 79 (31.0%) |  |
| **Shelter Status** |  |  |  | <0.001 |
| Living in Shelter | 4 (6.0%) | 58 (30.9%) | 62 (24.3%) |  |
| Not Living in Shelter (but displaced) | 22 (32.8%) | 45 (23.9%) | 67 (26.3%) |  |
| Not Displaced | 41 (61.2%) | 85 (45.2%) | 126 (49.4%) |  |
| **Number of Children** |  |  |  | 0.919 |
| Mean (SD) | 1.91 (1.29) | 1.98 (1.15) | 1.96 (1.18) |  |
| Median [Min, Max] | 2.00 [1.00, 10.0] | 2.00 [1.00, 6.00] | 2.00 [1.00, 10.0] |  |

**Supplementary Table 8. Participant Outcome Missingness Stratified by Trial Arm**

|  | **Control** | **Intervention** | **Overall** |
| --- | --- | --- | --- |
|  | **(N=255)** | **(N=255)** | **(N=510)** |
| **Caregiver Mental Health** |  |  |  |
| No Missingness | 249 (97.6%) | 248 (97.3%) | 497 (97.5%) |
| Missing Baseline | 3 (1.2%) | 2 (0.8%) | 5 (1.0%) |
| Missing Endline | 3 (1.2%) | 5 (2.0%) | 8 (1.6%) |
| **Parenting Practices** |  |  |  |
| No Missingness | 232 (91.0%) | 228 (89.4%) | 460 (90.2%) |
| Missing Baseline | 13 (5.1%) | 11 (4.3%) | 24 (4.7%) |
| Missing Both | 1 (0.4%) | 0 (0%) | 1 (0.2%) |
| Missing Endline | 9 (3.5%) | 16 (6.3%) | 25 (4.9%) |
| **Violence Against Children** |  |  |  |
| No Missingness | 247 (96.9%) | 245 (96.1%) | 492 (96.5%) |
| Missing Baseline | 4 (1.6%) | 4 (1.6%) | 8 (1.6%) |
| Missing Endline | 4 (1.6%) | 6 (2.4%) | 10 (2.0%) |
| **Displacement Status** |  |  |  |
| No Missingness | 254 (99.6%) | 255 (100%) | 509 (99.8%) |
| Missing | 1 (0.4%) | 0 (0%) | 1 (0.2%) |
| **Hopefulness about the Future and Coping with Grief** |  |  |  |
| No Missingness | 245 (96.1%) | 245 (96.1%) | 490 (96.1%) |
| Missing Baseline | 5 (2.0%) | 5 (2.0%) | 10 (2.0%) |
| Missing Endline | 4 (1.6%) | 5 (2.0%) | 9 (1.8%) |
| Missing Both | 1 (0.4%) | 0 (0%) | 1 (0.2%) |
| **Self Care** |  |  |  |
| No Missingness | 244 (95.7%) | 245 (96.1%) | 489 (95.9%) |
| Missing Baseline | 3 (1.2%) | 2 (0.8%) | 5 (1.0%) |
| Missing Endline | 8 (3.1%) | 8 (3.1%) | 16 (3.1%) |
| Missing Both | 0 (0%) | 0 (0%) | 0 (0%) |
| **Child Behavioural Issues** |  |  |  |
| No Missingness | 240 (94.1%) | 238 (93.3%) | 478 (93.7%) |
| Missing Baseline | 7 (2.7%) | 4 (1.6%) | 11 (2.2%) |
| Missing Endline | 6 (2.4%) | 12 (4.7%) | 18 (3.5%) |
| Missing Both | 2 (0.8%) | 1 (0.4%) | 3 (0.6%) |
| **Prevention of Violence Against Women *(asked at endline only)*** |  |  |  |
| No Missingness at Endline | 235 (92.2%) | 242 (94.9%) | 477 (93.8%) |
| Missingness at Endline | 20 (3.9%) | 13 (2.5%) | 33 (6.4%) |
| **Healthy Relationship Building *(asked to partnered participants)*** |  |  |  |
| No Missingness | 156 (94.5% | 166 (91.2%) | 322 (92.8%) |
| Missing Baseline | 1 (0.6%) | 2 (1.1%) | 3 (0.9%) |
| Missing Endline | 7 (4.2%) | 14 (7.7%) | 21 (6.1%) |
| Missing Both | 1 (0.6%) | 0 (0%) | 1 (0.3%) |
| **Nonviolent Discipline** |  |  |  |
| No Missingness | 246 (96.5%) | 247 (96.9%) | 493 (96.7%) |
| Missing Baseline | 3 (1.2%) | 2 (0.8%) | 5 (1.0%) |
| Missing Endline | 6 (2.4%) | 6 (2.4%) | 12 (2.4%) |
| Missing Both | 0 (0%) | 0 (0%) | 0 (0%) |
| **Positive Parenting** |  |  |  |
| No Missingness | 245 (96.1%) | 242 (94.9%) | 487 (95.5%) |
| Missing Baseline | 5 (2.0%) | 3 (1.2%) | 8 (1.6%) |
| Missing Endline | 5 (2.0%) | 10 (3.9%) | 15 (2.9%) |
| Missing Both | 0 (0%) | 0 (0%) | 0 (0%) |
| **Parental Monitoring** |  |  |  |
| No Missingness | 243 (95.3%) | 240 (94.1%) | 483 (94.7%) |
| Missing Baseline | 7 (2.7%) | 6 (2.4%) | 13 (2.5%) |
| Missing Endline | 4 (1.6%) | 9 (3.5%) | 13 (2.5%) |
| Missing Both | 1 (0.4%) | 0 (0%) | 1 (0.2%) |
| **Parental Involvement** |  |  |  |
| No Missingness | 242 (94.9%) | 244 (95.7%) | 486 (95.3%) |
| Missing Baseline | 5 (2.0%) | 2 (0.8%) | 7 (1.4%) |
| Missing Endline | 8 (3.1%) | 9 (3.5%) | 17 (3.3%) |
| Missing Both | 0 (0%) | 0 (0%) | 0 (0%) |

**Supplementary Table 9. Participant Outcome Missingness Stratified by Displacement Status**

|  | **Internally Displaced** | **Living in War-Affected Ukraine** | **Externally Displaced** | **Overall** |
| --- | --- | --- | --- | --- |
|  | **(N=138)** | **(N=221)** | **(N=150)** | **(N=509)** |
| **Caregiver Mental Health** |  |  |  |  |
| No Missingness | 135 (97.8%) | 216 (97.7%) | 145 (96.7%) | 496 (97.4%) |
| Missing Baseline | 1 (0.7%) | 3 (1.4%) | 1 (0.7%) | 5 (1.0%) |
| Missing Endline | 2 (1.4%) | 2 (0.9%) | 4 (2.7%) | 8 (1.6%) |
| **Parenting Practices** |  |  |  |  |
| No Missingness | 128 (92.8%) | 191 (86.4%) | 140 (93.3%) | 459 (90.2%) |
| Missing Baseline | 4 (2.9%) | 16 (7.2%) | 4 (2.7%) | 24 (4.7%) |
| Missing Endline | 6 (4.3%) | 13 (5.9%) | 6 (4.0%) | 25 (4.9%) |
| Missing Both | 0 (0%) | 1 (0.5%) | 0 (0%) | 1 (0.2%) |
| **Violence Against Children** |  |  |  |  |
| No Missingness | 135 (97.8%) | 211 (95.5%) | 145 (96.7%) | 491 (96.5%) |
| Missing Baseline | 1 (0.7%) | 6 (2.7%) | 1 (0.7%) | 8 (1.6%) |
| Missing Endline | 2 (1.4%) | 4 (1.8%) | 4 (2.7%) | 10 (2.0%) |
| **Hopefulness about the Future and Coping with Grief** |  |  |  |  |
| No Missingness | 134 (97.1%) | 211 (95.5%) | 144 (96.0%) | 489 (96.1%) |
| Missing Baseline | 2 (1.4%) | 6 (2.7%) | 2 (1.3%) | 10 (2.0%) |
| Missing Endline | 2 (1.4%) | 3 (1.4%) | 4 (2.7%) | 9 (1.8%) |
| Missing Both | 0 (0%) | 1 (0.5%) | 0 (0%) | 1 (0.2%) |
| **Self Care** |  |  |  |  |
| No Missingness | 134 (97.1%) | 210 (95.0%) | 144 (96.0%) | 488 (95.9%) |
| Missing Baseline | 1 (0.7%) | 3 (1.4%) | 1 (0.7%) | 5 (1.0%) |
| Missing Endline | 3 (2.2%) | 8 (3.6%) | 5 (3.3%) | 16 (3.1%) |
| Missing Both | 0 (0%) | 0 (0%) | 0 (0%) | 0 (0%) |
| **Child Behavioural Issues** |  |  |  |  |
| No Missingness | 131 (94.9%) | 203 (91.9%) | 143 (95.3%) | 477 (93.7%) |
| Missing Baseline | 3 (2.2%) | 8 (3.6%) | 0 (0%) | 11 (2.2%) |
| Missing Endline | 4 (2.9%) | 8 (3.6%) | 6 (4.0%) | 18 (3.5%) |
| Missing Both | 0 (0%) | 2 (0.9%) | 1 (0.7%) | 3 (0.6%) |
| **Prevention of Violence Against Women *(asked at endline only)*** |  |  |  |  |
| No Missingness at Endline | 133 (96.4%) | 198 (89.6%) | 146 (97.3%) | 477 (93.7%) |
| Missingness at Endline | 5 (3.6%) | 23 (10.4%) | 4 (2.7%) | 32 (6.3%) |
| **Healthy Relationship Building *(asked to partnered participants)*** |  |  |  |  |
| No Missingness | 69 (88.5%) | 135 (91.8%) | 118 (96.7%) | 322 (92.8%) |
| Missing Baseline | 1 (1.3%) | 1 (0.7%) | 1 (0.8%) | 3 (0.9%) |
| Missing Endline | 8 (10.3%) | 10 (6.8%) | 3 (2.5%) | 21 (6.1%) |
| Missing Both | 0 (0%) | 1 (0.7%) | 0 (0%) | 1 (0.3%) |
| **Nonviolent Discipline** |  |  |  |  |
| No Missingness | 135 (97.8%) | 211 (95.5%) | 146 (97.3%) | 492 (96.7%) |
| Missing Baseline | 0 (0%) | 5 (2.3%) | 0 (0%) | 5 (1.0%) |
| Missing Endline | 3 (2.2%) | 5 (2.3%) | 4 (2.7%) | 12 (2.4%) |
| Missing Both | 0 (0%) | 0 (0%) | 0 (0%) | 0 (0%) |
| **Positive Parenting** |  |  |  |  |
| No Missingness | 130 (94.2%) | 212 (95.9%) | 144 (96.0%) | 486 (95.5%) |
| Missing Baseline | 2 (1.4%) | 5 (2.3%) | 1 (0.7%) | 8 (1.6%) |
| Missing Endline | 6 (4.3%) | 4 (1.8%) | 5 (3.3%) | 15 (2.9%) |
| Missing Both | 0 (0%) | 0 (0%) | 0 (0%) | 0 (0%) |
| **Parental Monitoring** |  |  |  |  |
| No Missingness | 135 (97.8%) | 204 (92.3%) | 143 (95.3%) | 482 (94.7%) |
| Missing Baseline | 1 (0.7%) | 9 (4.1%) | 3 (2.0%) | 13 (2.6%) |
| Missing Endline | 2 (1.4%) | 7 (3.2%) | 4 (2.7%) | 13 (2.6%) |
| Missing Both | 0 (0%) | 1 (0.5%) | 0 (0%) | 1 (0.2%) |
| **Parental Involvement** |  |  |  |  |
| No Missingness | 135 (97.8%) | 205 (92.8%) | 145 (96.7%) | 485 (95.3%) |
| Missing Baseline | 1 (0.7%) | 6 (2.7%) | 0 (0%) | 7 (1.4%) |
| Missing Endline | 2 (1.4%) | 10 (4.5%) | 5 (3.3%) | 17 (3.3%) |
| Missing Both | 0 (0%) | 0 (0%) | 0 (0%) | 0 (0%) |
|  |  |  |  |  |
