# Supplementary material for: Effectiveness of the hope groups intervention on mental health, parenting practices, and violence against children among Ukrainian parents and caregivers affected by war and displacement: a cluster randomised controlled trial
Source: Lancet Reg Health Eur. 2026 Aug 25;68:101783. doi: 10.1016/j.lanepe.2026.101783 (PMC13559926; doi:10.1016/j.lanepe.2026.101783)
Supplement: Supplementary file 5 [file mmc2.docx]

**Supplementary Statistical Appendix**

1.1 Interim Analysis
1.2 Minor Revisions to Protocol and Statistical Analysis Plan

**1.1 Interim Analysis**

Our interim statistical analysis plan (<https://osf.io/2tb3g/overview>) provides full details on our procedures. In this supplementary appendix, we provide a summary of overarching guidelines and procedural steps taken to conduct the analysis.

**The overarching guidelines driving our interim analysis were as follows:**

1. If we find evidence of a harmful effect across primary outcomes, we will pause all activity to investigate and make adaptions.
2. If we find evidence of a protective effect of *Hope Groups* across primary outcomes, even after incorporation of sceptical priors^1,2,3^, this will be used to support the decision to conclude the trial after endline (1-week post-intervention completion), rather than continuing the trial throughout a long-term follow-up timepoint (which would require delaying intervention delivery to wait-list control clusters for an extended time period).
3. If we find evidence of a null effect, the trial will continue as planned to enable the sample size to reach the target number, and we will conduct another analysis with the full sample at trial endline. Thus, the interim analyses will not be used to consider stopping the trial early due to futility.

**The procedural steps taken were as follows:**

For both the interim analysis and the final analysis, the primary method of analysis was a two-level generalized linear mixed-effects model, which nested person within cluster, and controlled for baseline outcome measures. We included a fixed effect on the multi-categorical variable for facilitator. We used an intention-to-treat approach with a binary exposure variable indicating if the participant was randomized to the intervention or control group. Specifically for the interim analysis, we used rstanarm (v. 2.32.1) in R to implement our primary and secondary statistical model reported above using a Bayesian approach.

Steps were as follows:

1. We first utilised a noninformative informative prior on the treatment effect centred at 0 with a standard deviation of 2.5 (variance of 6.25) to assess if *Hope Groups* demonstrate a significant effect across outcomes indicated above, as measured by quantifying if posterior credible intervals of the treatment effect include the null with a probability of less than 5%.
2. To conduct interim analyses conservatively, when positive intervention effects were detected, we incorporated a sceptical prior on the treatment effect, which was centred at 0 with substantially stronger precision than under point (1) above to assert a sceptical belief about the intervention having an effect^1,2,3^, and assess if the significant effect(s) remain. This approach ensures that evidence needs to be overwhelming for the treatment effect to be called significant in interim analyses. To calculate the sceptical prior, we use the following formula: ^2,3^

$$prior: f\left( \theta\right)\mathcal{\sim N}\left( 0,\sigma^{2}/m_{0} \right)$$

$m_{0}= {(Z}_{\gamma}\sigma/\theta_{s})$^2^

where $Z_{\gamma}$ is the upper quantile of the standard normal (1.645 at $\alpha$= .05), $\sigma$ is the standard deviation, and $\theta_{s}$ is the estimated treatment effect.

In our pre-post study^4^, the largest standard deviation across primary outcomes was 2.85; to be conservative, we set $\sigma$=3.0. The smallest treatment effect across primary outcomes in the pre-post study was 1.13^4^; to be conservative and assume a 10-15% placebo effect from the pre-post study, we reduced this $\theta s$=1.0 – which also corresponds to the minimum treatment effect Ukrainian partners stated would make the intervention worth implementing. Thus, $m_{0}$ = 24.35423. Our final sceptical prior is:

*prior:* $f\left( \theta\right)\mathcal{\sim N}$ (0, $0.37$)

i.e. a normal distribution with a variance of 0.37 (equivalently a standard deviation of .61).

If any primary outcomes remained significant after incorporation of the sceptical prior, we considered there to be sufficient evidence to support providing *Hope Groups* to the control group immediately after trial endline without delaying them through a follow-up period, in light of the war context having detrimental impacts on caregiver mental health, safety, and violence against children, especially due to high mobility in populations which may result in control participants’ inability to participate if delayed through a follow-up period. We selected mental health, parenting, and/or violence against children outcomes due to their potential for acute impact on both the short-term and long-term health of caregivers and children.

**Assessing Harm**

After the interim analysis, study lead (ST) reviewed the interim results with two statistical advisors (OR and SF) to agree the data suggested no harm *(defined as no worsening outcomes among intervention participants compared to control participants)*. This review was to conduct a data-driven assessment of the safety of the intervention before continuing implementation to additional intervention participants and the wait-list control arm. No evidence of harm was detected; only evidence of beneficial impacts of the intervention across primary outcomes. (Results shown in Supplementary Materials Table 4)

**1.2 Minor Revisions to Protocol and Statistical Analysis Plan**

Our statistical analysis plans, both the interim analysis and the primary analysis, were registered within our registration on Open Science Framework prior to any analysis: <https://osf.io/2tb3g/overview>.

No major revisions were made to the statistical analysis plans after registration.

The only minor updates were as follows: 1) We decided to compile the six parenting items from Alabama Parenting Questionnaire into one summary Parenting Practices primary outcome. This decision was recommended by coauthor JL to be consistent with the field of parenting research, which most often analyses a combined score, rather than analysing subscales individually. All individual subscales (including positive parenting, parental involvement, parental monitoring, and nonviolent discipline) are still presented separately as secondary outcomes. 2) Prior to accessing the data, we determined to move nonviolent discipline into the secondary outcomes. While nonviolent discipline is an important measure for understanding violence against children, it could not be appropriately combined and analysed with the most relevant measures of emotional violence and physical violence (as it is a positive behaviour rather than a negative behavioural). Thus, rather than having four primary outcome models and further increasing the potential for Type I errors amidst our primary outcome analyses, we moved nonviolent discipline to a secondary outcome. This decision was made prior to analysis.

This was a pragmatic trial occurring in an active war setting. The trial was conceptualised in June 2023, obtained ethics approval in November 2023, and launched in November 2023. Data collection completed in July 2024, and analysis completed in November 2024.

References

1. Bendtsen, M. Avoiding Under- and Overrecruitment in Behavioral Intervention Trials Using Bayesian Sequential Designs: Tutorial. *J. Med. Internet Res.* **24**, e40730 (2022).
2. Parmar, M. K., Spiegelhalter, D. J. & Freedman, L. S. The CHART trials: Bayesian design and monitoring in practice. CHART Steering Committee. *Stat. Med.* **13**, 1297–1312 (1994).
3. Prior Distributions. in *Bayesian Approaches to Clinical Trials and Health-Care Evaluation* 139–180 (John Wiley & Sons, Ltd, 2003). doi:10.1002/0470092602.ch5.
4. Hillis, S. *et al.* The Effectiveness of Hope Groups, a Mental Health, Parenting Support, and Violence Prevention Program for Families Affected by the War in Ukraine: Findings from a Pre-Post Study. SSRN Scholarly Paper at https://doi.org/10.2139/ssrn.4677018 (2024).
