# Supplementary material for: Effectiveness of the hope groups intervention on mental health, parenting practices, and violence against children among Ukrainian parents and caregivers affected by war and displacement: a cluster randomised controlled trial
Source: Lancet Reg Health Eur. 2026 Aug 25;68:101783. doi: 10.1016/j.lanepe.2026.101783 (PMC13559926; doi:10.1016/j.lanepe.2026.101783)
Supplement: Supplementary file 6 [file mmc6.docx]

**Dear Lancet Regional Health,**

We request to include a collaborator group in our publication to honour the crucial contributions of all 30 Ukrainian coordinators and facilitators who implemented this intervention and collected survey data *amidst active war*.

We have provided all information below, including: a collaborator group name (“Hope Groups Implementation Team”) and a table with the first and last name of all individuals within the group, so that they may be indexed. We would like for this collaborator group to be listed with our author list.

**Collaborator Group Name:** Hope Groups Implementation Team

| **First Name** | **Last Name** |
| --- | --- |
| Alina | Ivanina |
| Oksana | Boichenko |
| Dariia | Halych |
| Yuliia | Kravchuk |
| Olena | Romanenko |
| Yana | Derzhak |
| Natalia | Lyagul |
| Natalia | Stupak |
| Svitlana | Yehorova |
| Iryna | Tokarchuk |
| Maryna | Masiuk |
| Anna | Yavorska |
| Antonina | Havrish |
| Liubov | Tishchenko |
| Marianna | Bondarenko |
| Nataliia | Lytvynenko |
| Oleg | Lyagul |
| Olena | Kharchenko |
| Tatiana | Yastrebova |
| Oksana | Kleymyonova |
| Olena | Krutii |
| Hanna | Dvoryanova |
| Iryna | Makarieieva |
| Liudmyla | Antoniuk |
| Zinaida | Antikalo |
| Yulia | Shevchenko - Szela |
| Oleksandr | Derkach |
| Svitlana | Derkach |
| Victoria | Tur |
| Mykola | Mohilevskiy |
