# Supplementary material for: Effectiveness of the hope groups intervention on mental health, parenting practices, and violence against children among Ukrainian parents and caregivers affected by war and displacement: a cluster randomised controlled trial
Source: Lancet Reg Health Eur. 2026 Aug 25;68:101783. doi: 10.1016/j.lanepe.2026.101783 (PMC13559926; doi:10.1016/j.lanepe.2026.101783)
Supplement: Supplementary file 7 [file mmc7.docx]

***Editorial Note:***

*This translation in Ukrainian was submitted by the authors and we reproduce it as supplied. It has not been peer reviewed. Our editorial processes have only been applied to the original abstract in English, which should serve as reference for this manuscript.*

**Короткий зміст**

**Передумови**

Майже дві третини дітей світу живуть у країнах, що постраждали від конфліктів, включаючи Україну, де дві третини дітей були переміщені після вторгнення росії у 2022 році. Щоб задовольнити нагальну потребу в ефективних підходах до зміцнення батьків та захисту дітей під час війни, була розроблена групова програма втручання у сфері психічного здоров’я, виховання дітей та запобігання насильству «Групи Надії» та використане кластерне рандомізоване контрольоване дослідження (cRCT) для оцінки її ефективності.

**Методи**

Було проведено двогрупове паралельне кластерне рандомізоване контрольоване дослідження (cRCT), що включало K=90 кластерів та N=510 батьків/опікунів, які є або внутрішньо переміщені особи, або біженці, або перебувають вдома в Україні в умовах воєнного стану. Первинні показники, отримані на початку дослідження та після завершення 12-сесійного втручання (кінцеве опитування), включали: психічне здоров'я опікунів, практики батьківства та рівень насильства щодо дітей. Кластери були випадковим чином розподілені на експериментальну групу, де здійснювалося втручання за програмою «Груп Надії» (K=45; N=255) та контрольну групу зі списку очікування (K=45; N=255). Проміжні підсумки були проведені після того, як перші 20 кластерів досягли кінцевого опитування, передбаченого дослідженням. Дослідження було завчасно зареєстровано в Open Science Framework (https://osf.io/uvj67), а згодом зареєстровано на clinicaltrials.gov відповідно до рекомендацій ICJME (NCT07470333).

**Результати**

Після завершення 12-сесійного втручання, програма «Груп Надії» продемонструвала середній та значний вплив на всі вимірювані первинні показники порівняно з контрольною групою. Опікуни в експериментальній групі вказали на 57,4% нижчий рівень батьківської депресії та тривоги (β=-2·85; 95% CI=-3·49,-2·22; d=-·86); на 52,7% нижчий рівень насильства щодо дітей (β=-·5; 95% CI=-·64,-·36; d=-·43); на 33% підвищення практик батьківства (β=1·32, 95% CI=0·92,1·71, d=·57) порівняно з контрольною групою. Рівень реагування був високим: 98·6%. Не було жодних побічних ефектів чи шкоди.

**Інтерпретація**

Результати показують, що втручання за програмою «Груп Надії» покращує психічне здоров'я опікунів, зміцнює практики батьківства та зменшує насильство щодо дітей в умовах воєнного стану та переміщення.
